# Chemical Probe for Imaging of Polo-like Kinase 4 and Centrioles

**DOI:** 10.1021/jacsau.3c00271

**Published:** 2023-08-04

**Authors:** Aleksandar Salim, Philipp Werther, Georgios N. Hatzopoulos, Luc Reymond, Richard Wombacher, Pierre Gönczy, Kai Johnsson

**Affiliations:** † Department of Chemical Biology, Max Planck Institute for Medical Research, Jahnstrasse 29, Heidelberg 69120, Germany; ‡ Institute of Chemical Sciences and Engineering (ISIC), École Polytechnique Fédérale de Lausanne (EPFL), Lausanne 1015, Switzerland; § Institute of Pharmacy and Molecular Biotechnology, Heidelberg University, Im Neuenheimer Feld 364, Heidelberg 69120, Germany; ∥ Swiss Institute for Experimental Cancer Research (ISREC), School of Life Sciences, Swiss Federal Institute of Technology Lausanne (EPFL), Lausanne CH-1015, Switzerland

**Keywords:** protein labeling, bio-orthogonal chemistry, fluorogenic fluorophores, Plk4, centriole imaging

## Abstract

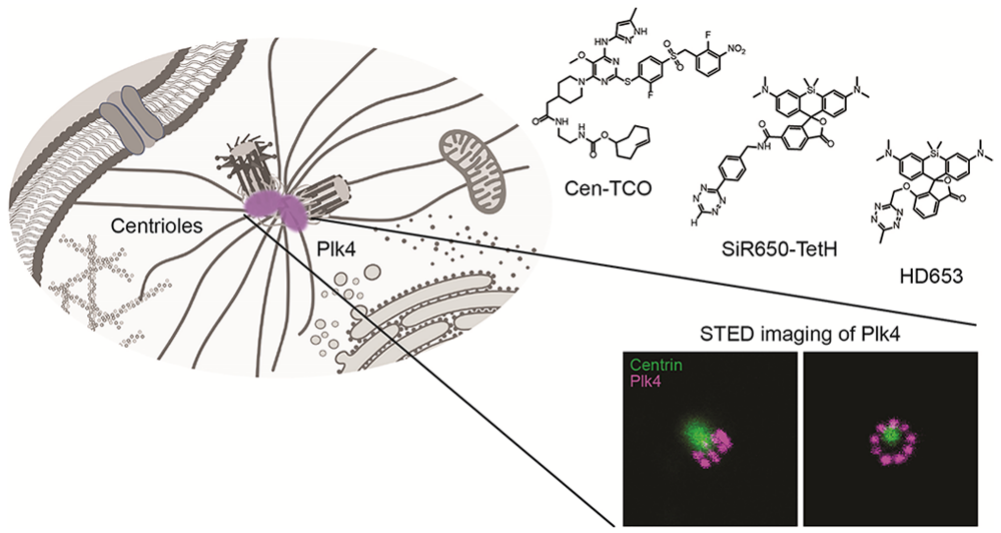

Polo-like kinase (Plk4) is a serine/threonine-protein kinase that is essential for biogenesis of the centriole organelle and is enriched at centrioles. Herein, we introduce Cen-TCO, a chemical probe based on the Plk4 inhibitor centrinone, to image Plk4 and centrioles in live or fixed cultured human cells. Specifically, we established a bio-orthogonal two-step labeling system that enables the Cen-TCO-mediated imaging of Plk4 by STED super-resolution microscopy. Such direct labeling of Plk4 results in an increased resolution in STED imaging compared with using anti-Plk4 antibodies, underlining the importance of direct labeling strategies for super-resolution microscopy. We anticipate that Cen-TCO will become an important tool for investigating the biology of Plk4 and of centrioles.

## Introduction

**Figure 1:**
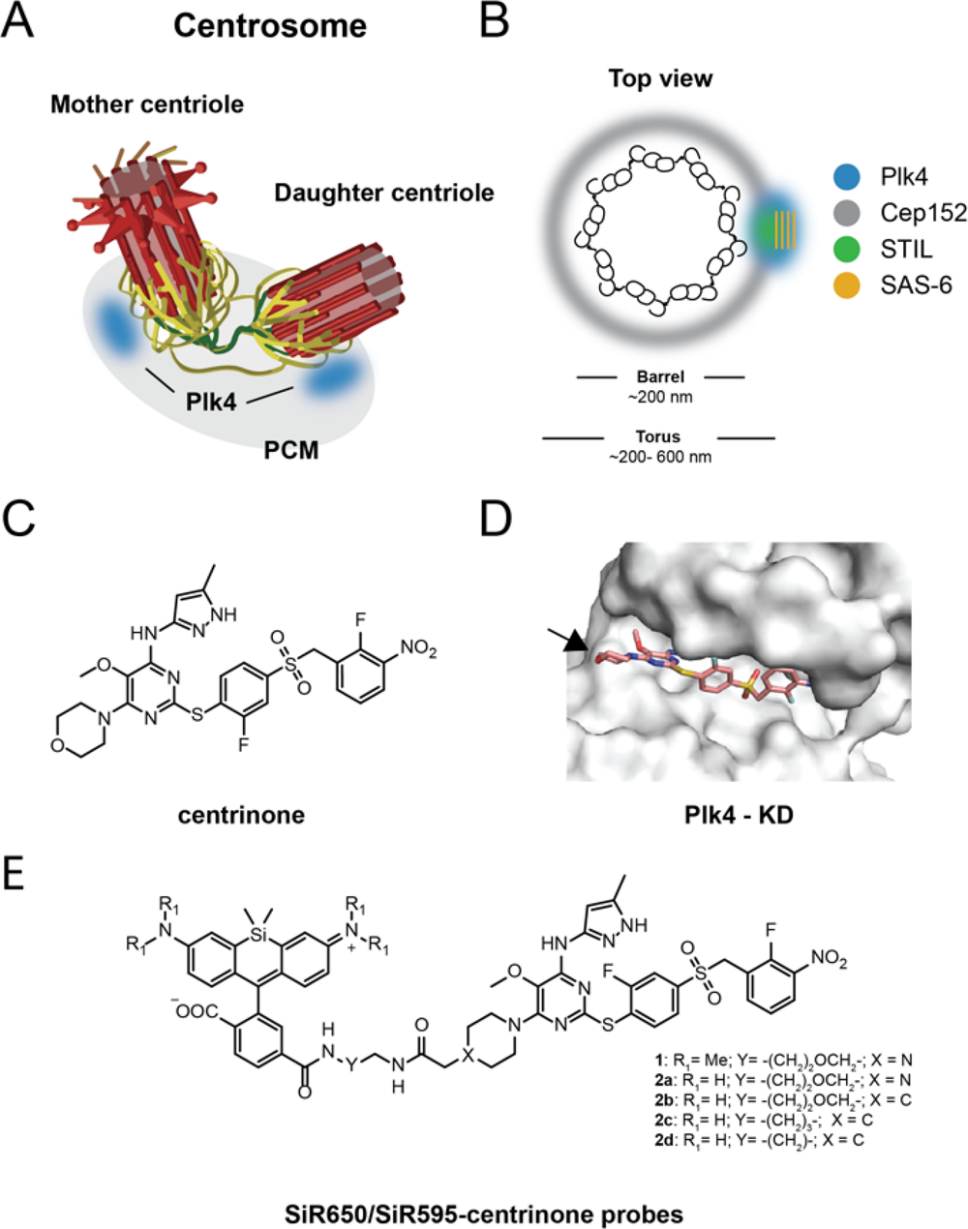
Initial probe design based on centrinone for labeling Plk4. (A) Schematic representation of the centrosome (adapted from ref (5)). (B) Top view of the centriolar proximal part with localizations of relevant proteins in this study. (C) Structure of centrinone. (D) Crystal structure of centrinone bound to the Plk4 kinase domain (PDB: 4yur) zoomed in the Plk4 active site. Arrow indicates the morpholine ring. (E) Structures of SiR595/SiR-centrinone probes.

Centrioles are microtubule-based structures with numerous critical functions, including in cell proliferation, division, and signaling.^1−3^ Together with their surrounding protein matrix, termed the pericentriolar material (PCM), a pair of centrioles forms the centrosome of many animal cells (Figure 1A).^1−3^ Centrosomes act as microtubule-organizing centers (MTOC) and thus play a crucial role in the formation of the microtubule-based bipolar mitotic spindle. Centriole numbers need to be under tight control for proper cell division. Centriole duplication occurs once per cell cycle and begins at the G1/S transition with the formation of a procentriole orthogonal to each pre-existing centriole.

A critical protein for the onset of centriole biogenesis in human cells is polo-like kinase 4 (Plk4), a serine/threonine protein kinase that is enriched through most of the cell cycle around the proximal region of centrioles, on a torus that harbors notably the coiled-coil protein Cep152 (Figure 1B).^4,5^ Loss of Plk4 or inhibition of its kinase activity by the selective inhibitor centrinone results in failure of procentriole formation.^6−8^ Conversely, Plk4 overexpression leads to centriole overamplification.^10,11^ Normally, Plk4 levels are tightly regulated to ensure the formation of one and only one procentriole per pre-existing centriole per cell cycle. Depending on the cell type, there are ∼1200 to ∼5000 copies of Plk4, but only a fraction of those reside at the centrosome.^9^

Plk4 levels are self-regulated notably by autophosphorylation of its kinase domain, which primes it for degradation via the E3 ubiquitin ligase SCF^β-TrCP^ (also known as F-box/WD repeat-containing protein 1A).^12−15^ Due to such tight regulation, Plk4 levels at centrioles are very low (49 ± 11 copies per centrosome in KE 37 cells), rendering its detection challenging.^9^

During part of the G1 phase of the cell cycle, Plk4 exhibits a ring-like distribution, which when observed with super-resolution microscopy, seems to be comprised of discrete foci with a 6-fold rotational symmetry.^16^ At the onset of procentriole formation, Plk4 phosphorylates SCL-interrupting locus protein (STIL), which leads to the formation of a so-called STIL-Plk4 module that localizes as a single focus on the torus, around the proximal end of the pre-existing centriole (Figure 1B).^12,17,18^ Therefore, PLK4 transitions from a ring pattern to a single focus together with STIL. Thereafter, HsSAS-6, the main building block of the centriolar cartwheel that templates the 9-fold symmetry of the entire organelle, is thought to be then recruited to this single focus.^12,17,18^ In this manner, the Plk4-STIL-HsSAS-6 complex serves as an assembly site for procentriole formation.^19,20^ Although the above interpretation is compatible with most of the experimental evidence, several aspects of Plk4 biology remain elusive. A fluorescent probe that would allow one to label Plk4 with a high spatiotemporal resolution would be a powerful tool to further elucidate steps at the onset of centriole biogenesis.

Herein, we report novel small-molecule-based probes targeting Plk4. An initial approach based on one-step labeling allowed us to mark overexpressed Plk4 in live cells and reveal its 9-fold symmetrical arrangement around the proximal part of the pre-existing centriole by STED microscopy. A second approach based on a two-step labeling strategy enabled us to generate super-resolved images of endogenous Plk4 at different cell cycle stages, including in fixed cells. Overall, the newly developed probes enable the monitoring of Plk4 and centrioles in live or fixed cells with unmatched resolution.

## Results and Discussion

### Design and Characterization of SiR595-Centrinone Probe

We chose the selective and potent Plk4 inhibitor centrinone as a starting point to develop probes targeting the protein’s kinase domain (Figure 1C).^8^ By analyzing the reported crystal structure of the Plk4 kinase domain bound to centrinone,^8^ we identified that the most suitable position to functionalize centrinone is via its solvent-exposed morpholine ring (Figure 1D, arrow). We therefore designed several centrinone-based probes in which a fluorogenic fluorophore is conjugated at this position (Figure 1E). To optimize the fluorogenic response and hydrophobicity of the probes, we varied the linker type and length, conjugation handle, and fluorophore (Figure 1E). Following the synthetic route depicted in Scheme S1, we obtained a set of fluorescent centrinone probes (**1**, **2a-d**) (Table 1). The first generation of probes, SiR650-PEG-N-Cen (**1**) and SiR595-PEG-N-Cen (**2a**), when added to live human cells in culture, exhibited high unspecific labeling, most likely due to accumulation in endolysosomal compartments (Figure S1A, B). To reduce unspecific labeling and increase permeability of the probes, we replaced SiR650 with silicon-rhodamine595 (SiR595),^21^ a more fluorogenic dye that exists predominantly in the closed spirolactone form (Figure S2).^22^

**Table 1:** Characterization of Centrinone-Based Chemical Probes

Probe name	*F*_PIk4_/*F*_BSA_^a^	*K*_d_ (nM)
**1** SiR650-PEG-N-Cen	4 ± 1	48 ± 6
**2a** SiR595-PEG-N-Cen	10 ± 1	26 ± 3
**2b** SiR595-PEG-C-Cen	23 ± 1	14 ± 4
**2c** SiR595–C4–C-Cen	14 ± 2	23 ± 6
**2d** SiR595–C2–C-Cen	36 ± 1	38 ± 5
**3** Cen-TCO	N.A.	8 ± 1
centrinone	N.A.	0.9 ± 0.2

a Results represent the mean of *F*_(+Plk4KD)_/*F*_(+BSA)_ and standard deviation (*N* = 3).

**Figure 2:**
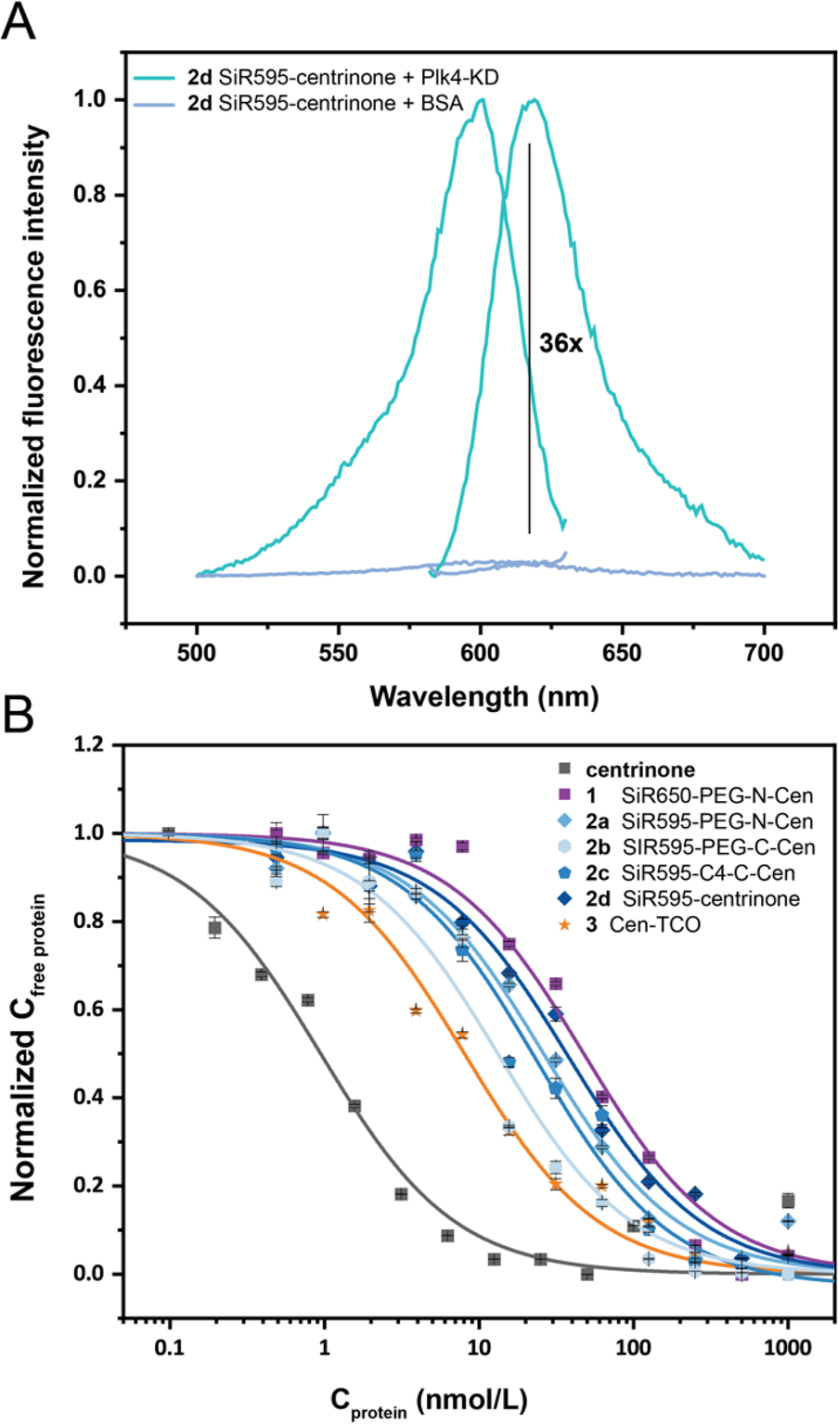
Fluorogenicity and binding affinity of developed dye-centrinone probes. (A) Excitation and emission spectra of SiR595-centrinone (**2d**) with Plk4-KD (20 μM) or bovine serum albumin (BSA, 2 mg/mL). (B) Competition binding assays of Alexa488-PEG-N-centrinone (**27**) (1 nM) and Plk4-KD (5 nM) titrated with different concentrations of centrinone, Cen-TCO (**3**) and dye-centrinone probes.

**Figure 3:**
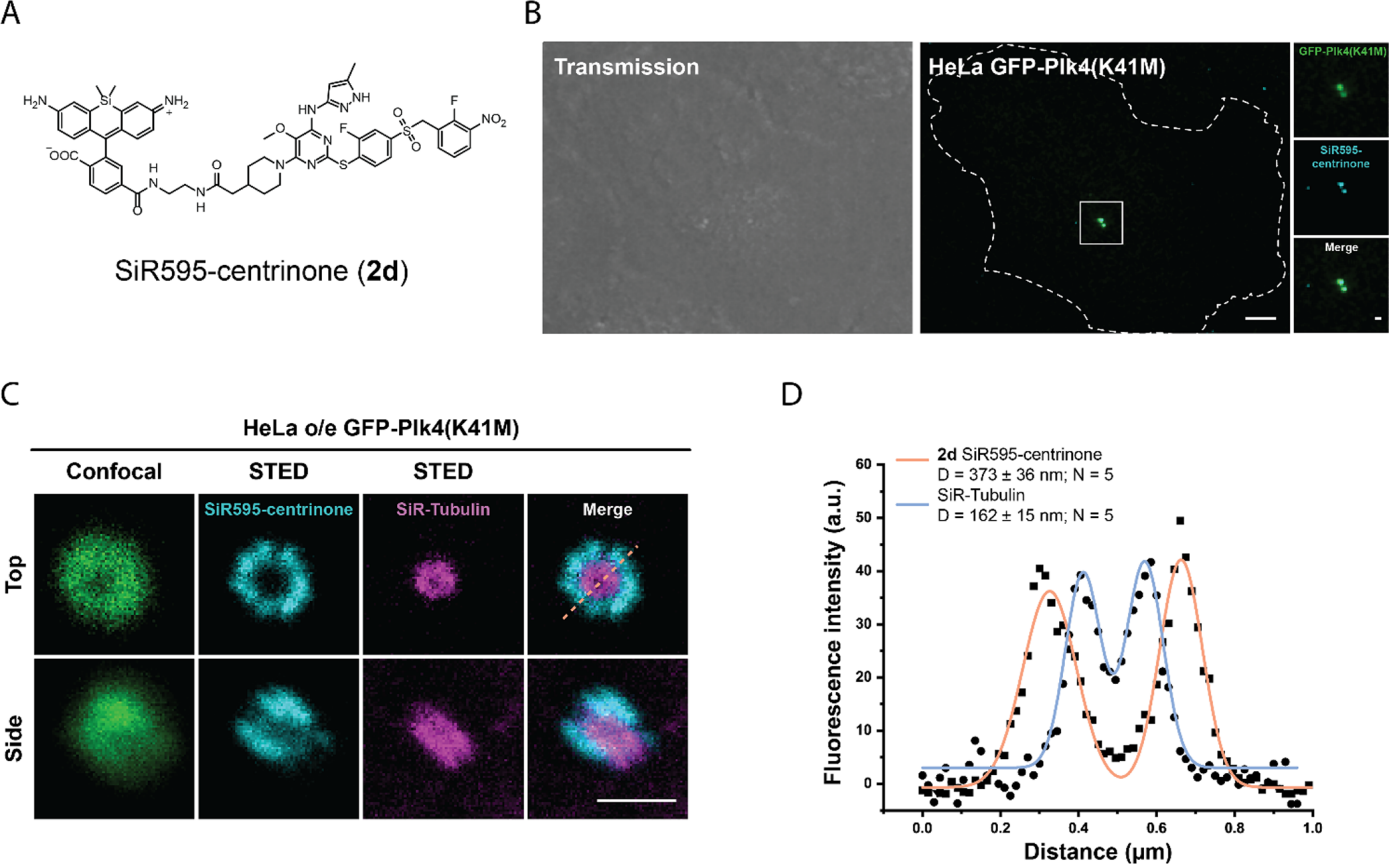
Live-cell imaging of overexpressed Plk4. (A) Structure of SiR595-centrinone (**2d**). (B) Live cell confocal image of HeLa cells transfected with GFP-Plk4(K41M) and labeled with SiR595-centrinone (**2d**). Scale bar: 5 μm. (C) Live cell confocal dual color STED images of centrioles in HeLa cells overexpressing GFP-PLK4 (K41M) labeled with SiR595-centrinone (500 nM) and SiR650-Tubulin (1 μM). Cells were not washed prior to imaging. Merge represents overlap of SiR595 and SiR650 channels. Scale bar: 500 nm. (D) Fluorescence intensity profile of the orange line in panel C for SiR595 and SiR650 channel, as well as mean diameter of the GFP-Plk4 (K41M) signal ± s.d. Represented STED images are not deconvoluted.

Moreover, we substituted the basic acetyl-piperazine nitrogen in compounds (**1** and **2a**) with an acetyl-piperidine, to avoid probe protonation and accumulation in endolysosomal compartments (Figure S1A, B).^23^ This yielded a second generation of centrinone-based probes (compounds **2b**–**d**). These probes were characterized first *in vitro* by measuring their fluorogenicity in the presence of the 6xHis-tagged Plk4 kinase domain (KD) (Table 1). SiR595-centrinone (**2d**) exhibited the highest fluorogenic response with a 36-fold (±1) increase in fluorescence upon binding (Figure 2A, Table 1). These data suggest that shorter linkers lead to a higher fluorogenic response of the probes. Next, we measured the binding affinities of the probes to the Plk4 kinase domain via a competition binding assay based on fluorescence polarization. As shown in Figure 2B, all probes exhibited binding affinities in the low nanomolar range, which is 15- to 50-fold lower than that of unmodified centrinone. Due to the high fluorogenicity of SiR595-centrinone (**2d**) and its binding affinity of 38 ± 5 nM (Figure 2B, Table 1), we focused on this probe for further live cell experiments (Figure 3).

### Live-Cell Imaging of Overexpressed Plk4 with SiR595-Centrinone

We first evaluated the performance of SiR595-centrinone (**2d**) in live HeLa cells but failed to observe specific labeling of endogenous Plk4. This could reflect the very low expression level of Plk4 and/or insufficient affinity of the probe. To circumvent this limitation, we transfected HeLa cells with a GFP-tagged kinase-dead variant of Plk4, GFP-Plk4(K41M); this constructs interferes with Plk4 degradation, resulting in elevated levels of the kinase at centrioles, but without causing centriole overduplication owing to the lack of kinase activity.^10^ HeLa cells transfected with GFP-Plk4(K41M) were incubated with 500 nM SiR595-centrinone (**2d**) for 1 h and then imaged by confocal microscopy, without washing of the probe. As shown in Figure 3B and Figure S3A, we could successfully colocalize the SiR595 signal with the GFP signal of Plk4(K41M), with minimal unspecific signal. Moreover, we found that SiR595-centrinone labels overexpressed Plk4-GFP, the active form of Plk4 (Figure S3B, C). Furthermore, in the same experimental setup, we tested whether SiR595-centrinone (**2d**) is suitable for live-cell STED imaging, which would allow us to better resolve the Plk4 distribution at centrioles. To this end, we labeled HeLa cells transiently transfected with GFP-Plk4(K41M) line with 500 nM SiR595-centrinone (**2d**) and 1 μM SiR650-Tubulin.^24^ STED imaging revealed a 9-fold symmetric distribution of the SiR595 signal around the proximal part of the pre-existing centriole (Figure 3C). The SiR595 signal surrounds the SiR650 signal coming from the labeling of centriolar microtubules (Figure 3C, top). The diameter of the circular SiR595 signal was 373 ± 36 nm, while that of the SiR650-Tubulin signal was 162 ± 14 nm (Figure 3D, *N* = 5). This suggests that accumulated Plk4 is loaded on the torus, likely following interaction with its molecular partner Cep152,^25^ and arranges itself in a 9-fold symmetrical pattern.

Having established that SiR595-centrinone (**2d**) can successfully label overexpressed Plk4, we wanted to investigate whether it also marks endogenous Plk4. To assess the degree of Plk4 binding by the probe, we used the cellular phenotypes caused by either SiR595-centrinone (**2d**) or centrinone as a proxy for efficient binding. As previously mentioned, Plk4 inhibition by centrinone prevents procentriole formation, resulting in daughters cells with a single centriole each.^26^ In contrast, partial Plk4 inhibition leads to increased levels of centriolar Plk4 due to partial interference with protein degradation mediated by autophosphorylation, overall resulting in centriole overamplification.^8^ Therefore, both under-duplication and overamplification of centrioles can be used as a proxy for SiR595-centrinone binding to endogenous Plk4. We incubated HeLa cells expressing the centriolar marker Centrin1-GFP with either SiR595-centrinone (**2d**) (2 μM, to ensure an excess of the compound), centrinone (250 nM), or DMSO for 24 h, followed by scoring of the number of Centrin1-GFP foci (Figure S7A). We observed that SiR595-centrinone (**2d**) did not affect centriole numbers, further indicating that SiR595-centrinone (**2d**) does not bind efficiently to endogenous Plk4. This is most likely due to its reduced binding affinity, as the K_D_ is 42-fold higher than that of unmodified Centrinone (Table 1), as a result of steric hindrance by the SiR595 derivatization. We conclude that the failure of SiR595-centrinone (**2d**) to detect endogenous Plk4 is due to the low binding affinity of the probe in combination with low protein levels. Following the observation that derivatization of centrinone with fluorophores leads to a significant loss of binding affinity, we decided to develop a two-step, bio-orthogonal labeling strategy. In the first step, Plk4 is labeled with a centrinone derivative containing the strained alkene trans-cyclooct-2-ene (TCO), which should be sterically less bulky than the fluorophore and possess higher cell permeability. In the second step, the Plk4-bound molecule is specifically reacted with an appropriate fluorogenic tetrazine dye.^27−33^ This strategy was applied previously successfully to label various protein targets in live and fixed cells,^30,32,34−38^ and we reasoned that a smaller and potentially more permeable Cen-TCO (**3**) derivative would likewise enable more efficient labeling of endogenous Plk4.

### Development of Two-Step Labeling System for Plk4 Imaging

**Figure 4:**
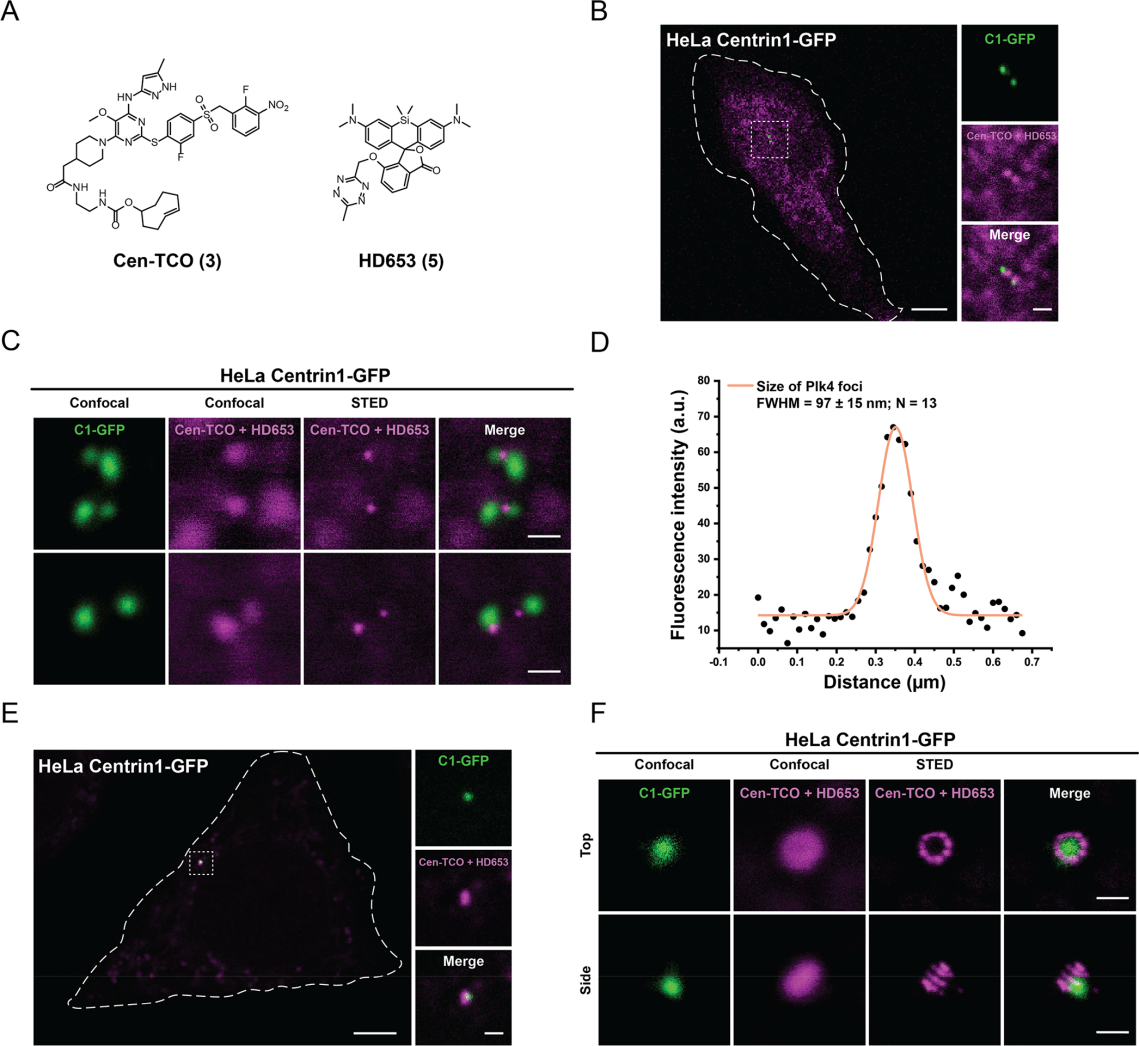
Live-cell imaging of endogenous Plk4 with the Cen-TCO/HD653 labeling system. (A) Structures of Cen-TCO (**3**) and HD653 (**5**). (B, C) Live cell confocal (B) and STED (C) single plane images of HeLa cells expressing the centriolar marker Centrin1-GFP and labeled with Cen-TCO (**3**) (500 nM) for 1 h, followed by 15 min incubation with HD653 (**5**) (500 nM). Scale bars: 5 μm (B); insert 1 μm; 500 nm (C). (D) Fluorescence intensity profile of the orange line in panel C. Mean fwhm of the Plk4 foci intensity labeled with Cen-TCO/HD653 ± s.d.; *N* number of foci measured on images obtained in multiple experiments. (E, F) Live cell confocal (E) and STED (F) images of HeLa cells expressing Centrin1-GFP to mark centrioles and labeled with Cen-TCO (**3**) (500 nM) for 24 h followed by 15 min incubation with HD653 (**5**) (500 nM). Cells were imaged after a brief wash with warm culture media. Merge represents overlap of confocal GFP signal and STED image of SiR650 channel. Scale bar: (E) 5 μm, 1 μm, (F) 500 nm. STED images represented are not deconvoluted.

**Figure 5:**
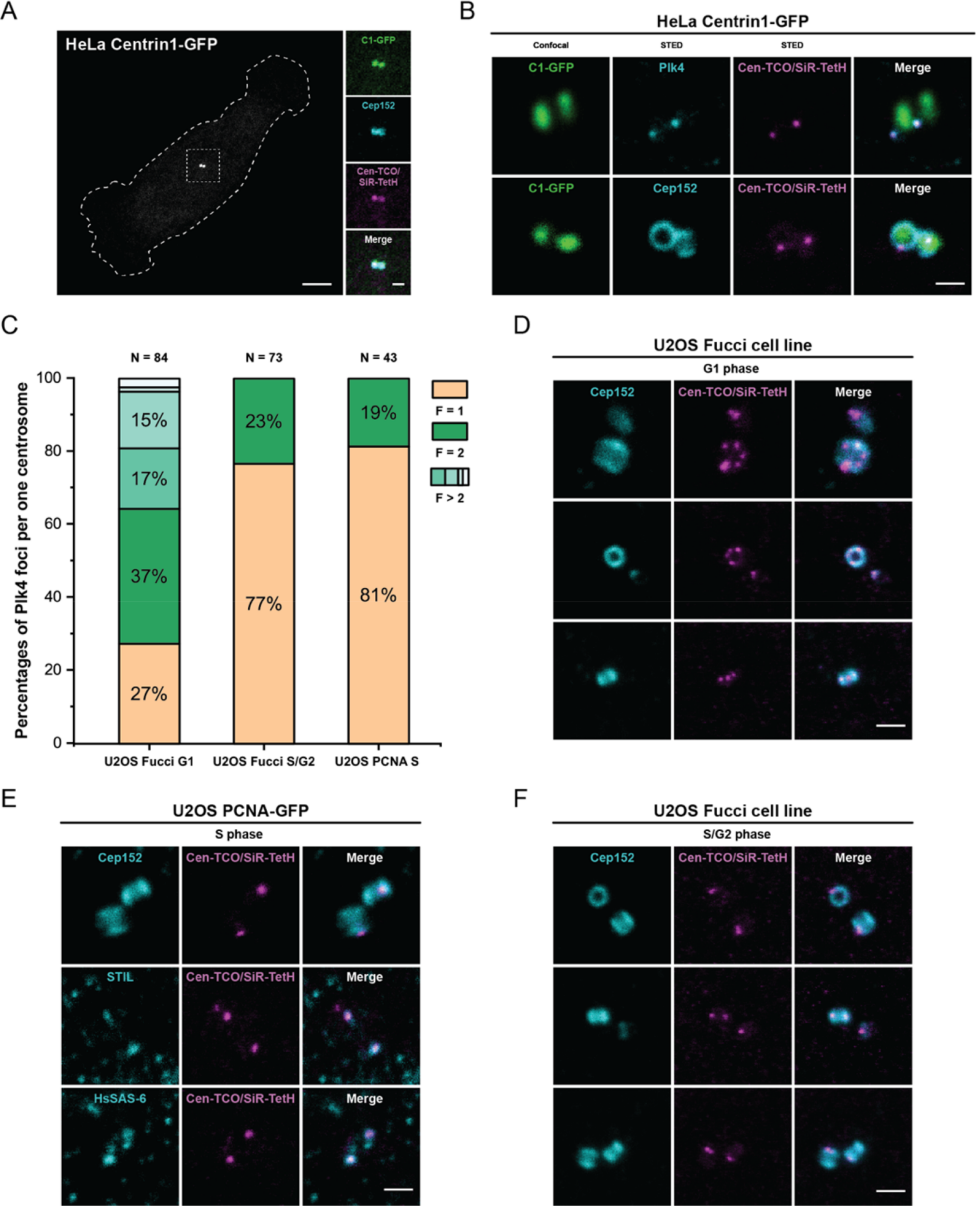
Confocal & STED imaging of Plk4 labeled with CenTCO/SiR650-TetH labeling system in different cell lines and cell cycle stages. (A) Confocal images of HeLa cells expressing Centrin1-GFP as a centriolar marker. Cells were stained with Cen-TCO (**3**) (500 nM) for 1 h and then fixed with 2% PFA and immunostained with Cep152 antibodies together with SiR650-TetH (**4a**) (200 nM). Scale bars 5 μm; inset 1 μm. (B) Confocal & STED images of HeLa cells expressing Centrin1-GFP labeled with Cen-TCO (**3**) (500 nM) for 1 h, then fixed with 2% PFA and stained with SiR650-TetH (**4a**) (200 nM) and antibodies against Plk4 (upper row) or Cep152 (lower row). Scale bar: 500 nm. (C) Scoring of Plk4 foci numbers per centriole at different cell cycle stages of Fucci & PCNA U2OS cell lines and corresponding representative STED images (C–F). Note here that data indicates that the amount of two Plk4 foci are mildly increased (∼10% to ∼20% of measured samples) in comparison to the data obtained in similar conditions in HeLa Centrin1-GFP cell line (Figure S7D; 2 h Cen-TCO (**3**)). We speculate that this might be due to higher basal levels of Plk4 in U2OS cells.^9^ (D) STED imaging of U2OS cells expressing mTurquoise-Cdt1 as G1 cell cycle phase marker (not shown) labeled with CenTCO/SiR650-TetH and anti-Cep152 antibodies. Scale bar: 500 nm; (E) STED imaging of U2OS cells expressing Clover-Geminin as S/G2 phase cell cycle marker (not shown) labeled with Cen-TCO (**3**) and SiR650-TetH (**4a**) and anti-Cep152 antibodies. Scale bar: 500 nm. (F) STED imaging of U2OS cells expressing PCNA-GFP as S phase cell cycle phase marker (not shown) labeled with CenTCO (**3**) and SiR650-TetH (**4a**) and upper panels: anti-Cep152 antibodies, middle panels: anti-STIL antibodies, and lower panels: anti-HsSAS-6 antibodies. Scale bar: 500 nm. STED images shown are not deconvoluted.

We conjugated *trans*-cyclooctene to the previously obtained compound (**4**) to obtain Cen-TCO (**3**) using the synthetic route outlined in Scheme S2. The resulting Cen-TCO (**3**) reacted with an appropriate tetrazine, such as SiR650-TetH (**4a**) (Figure S4A). The reaction was followed by LC-MS, and the data confirmed quantitative conversion of Cen-TCO (**3**) to the appropriate cycloaddition product Cen-Click-SiR650 (Figure S4A, D). The binding affinity of Cen-TCO (**3**) to the Plk4 kinase domain was measured *in vitro* to be of 8 ± 1 nM, hence 4-fold higher than that of SiR595-centrinone (**2d**) (Table 1) but ∼9-fold lower than that of centrinone. To assess the phenotype caused by Cen-TCO (**3**), we incubated HeLa cells expressing Centrin1-GFP with different concentrations of Cen-TCO (**3**) for 24 h, followed by scoring of Centrin1-GFP foci numbers (Figure S5B). We found that incubation with a concentration of Cen-TCO (**3**) ≥ 500 nM caused the expected centriole under-duplication phenotype, to a similar level as centrinone at concentrations ≥250 nM (Figure S4A, B). This indicates that Cen-TCO (**3**) binds to endogenous Plk4, validating the first step of the bio-orthogonal labeling strategy. Next, we tested Cen-TCO (**3**) in combination with different tetrazine partners to determine the optimal combination for our bio-orthogonal strategy. We used four different tetrazines to visualize overexpressed GFP-Plk4(K41M) in HeLa cells following 1 h of incubation with Cen-TCO (500 nM) (Figure S6). These experiments indicated that both SiR650-TetH (**4a**) and HD653 (**5**) exhibit the centriolar signal, while HD653 exhibits the lowest unspecific signal (Figure S6). Furthermore, the high fluorogenicity of HD653 makes it particularly well suited for live cell imaging.^33^ Therefore, the combination of Cen-TCO (**3**) and HD653 (**5**) was further used for live cell imaging of endogenous Plk4 (Figure 4A). HeLa cells expressing Centrin1-GFP were incubated with 500 nM Cen-TCO (**3**) for 1 h, followed by a 15 min incubation with 500 nM HD653 (**5**) and imaged with a confocal microscope without prior washing. Despite the high unspecific background signal, a clear focus could be detected at centrioles (Figure 4B). Performing an additional washing step prior to imaging could not further reduce the background signal. STED microscopy significantly increased image quality of labeled endogenous Plk4, as the weaker unspecific signal was suppressed by the 775 nm STED laser (Figure 4C). Furthermore, STED imaging allowed us to measure the fwhm of the Plk4 focus to be 97 ± 16 nm (N = 9) (Figure 4D). Next, we asked whether the click product Cen-Click-SiR650 also labels Plk4 when incubated with HeLa cells expressing Centrin1-GFP as a centriolar marker (Figure S5C). However, STED imaging did not show successful labeling of Plk4 by Cen-Click-SiR650 (Figure S5C), suggesting that the two-step approach is essential for the successful labeling of Plk4. As previously mentioned, inhibition of Plk4 by centrinone results in Plk4 accumulation around the proximal part of pre-existing centrioles and prevents procentriole formation.^8,39^ To image such Plk4 accumulations, we incubated HeLa cells expressing Centrin1-GFP with 500 nM Cen-TCO for 24 h, followed by a 15 min incubation with 500 nM HD653 (**5**). Live-cell confocal imaging revealed foci of labeled Plk4 colocalizing with Centrin1-GFP, with practically no background signal (Figure 4E). Furthermore, STED imaging revealed a 9-fold symmetry arrangement of such labeled Plk4 (Figure 4F and Figure S7). The fwhm of the circular signal in this case was 368 ± 26 (N = 6) (Figure S7B), which corresponds to the size measured with overexpressed GFP-Plk4(K41M) (see Figure 3D), and approximately that of the torus region surrounding the centriole.^40^ Another benefit of our bio-orthogonal strategy is that it could be compatible with fixation protocols. This would enable super-resolution localization of Plk4 in relation to common centriolar proteins marked via immunostaining. In order to explore the feasibility of this approach, we used HeLa cells expressing Centrin1-GFP incubated with 500 nM Cen-TCO (**3**) for 1 h followed by fixation with 2% paraformaldehyde (PFA). Cells were then permeabilized and labeled with primary antibodies against the torus protein Cep152, together with either HD653 (**5**) (200 nM) or the commercially available SiR650-TetH (**4a**) (200 nM), followed by incubation with secondary antibodies. Confocal imaging demonstrated the colocalization of the Cep152 signal with the Plk4 signal in both cases (Figure 5A). STED imaging of Plk4 labeled with Cen-TCO/SiR650-TetH or PLk4 antibodies shows colocalization of the two signals (Figure 5B, upper panel) and concurring localization on the torus.^39,41^ The specificity of the labeling in these experiments was confirmed by competition experiments with underivatized centrinone (Figure S8A). Similar to centrinone, Cen-TCO inhibits Plk4 upon binding, which is expected to affect centriolar level which consequently alters its distribution. To assess this and test whether early time points after Cen-TCO addition may be immune to this problem, HeLa Centrin1-GFP cells were incubated with Cen-TCO (**3**) and fixed at different time points thereafter, after which the number of centriolar Plk4 foci per cell was quantified (Figure S8B–F). After 1 h of incubation with Cen-TCO (**3**), no perturbation of Plk4 foci numbers was observed (Figure S8B). Up to 4 h of incubation, only minor perturbations in Plk4 foci numbers were observed (Figure S8B–E). By contrast, after 4 h, a significant increase in Plk4 foci numbers around centrioles was observed (Figure S8B, F). We conclude that brief labeling with 500 nM Cen-TCO for less than 4 h does not affect Plk4 levels and localization. However, longer incubations trap Plk4 in an inhibited state, leading to an accumulation at the centriole. We used the Cen-TCO/SiR650-TetH brief labeling strategy to examine Plk4 distribution at different stages of the cell cycle in detail. Plk4 protein levels increase during the G1 phase of the cell cycle, with the protein being present as a ring around the proximal part of the pre-existing centriole.^17,41,42^ Approximately at the G1 to S phase transition, Plk4 gradually focuses into a single spot together with STIL, thus generating a sole recruitment site for HsSAS-6.^17,41^ During S phase, these three proteins appear to colocalize and form the basis of the procentriole, which is observed as a single focus by STED imaging.^43^ To analyze potential changes in Plk4 localization between G1 and S phases, we used a Fucci U2OS cell line expressing the G1 marker mTurqoise-Cdt1 and the S/G2 marker Clover-Geminin (Figure S9A).^44^ Plk4 was labeled with Cen-TCO/SiR650-TetH (500 nM Cen-TCO for 2 h followed by 200 nM TetH) and cells costained with anti-Cep152 antibodies, followed by analysis with STED microscopy (Figure 5C). This revealed that in >70% of G1 cells, two or more Plk4 foci localize on the Cep152 torus (Figure 5C, D). Also, our results indicate that only 3% of G1 cells harbored 6 or more foci (Figure 5C, D), indicating that the 9-fold symmetrical distribution observed upon overexpression of Plk4 likely represents binding to the lower affinity sites populated by the endogenous protein. Interestingly, the distribution of foci numbers suggests that the localization of Plk4 at different locations on the torus is transient, with the focusing to a single spot starting already in G1. This is very evident in some cells, in which a single high intensity focus is observed (Figure 5D, upper panel). In line with the current model of transition from a broad to a tight localization on the torus, costaining cells in S phase with STIL and HsSAS-6 confirms that the Plk4 focus is present at the base of the procentriole (Figure 5E), while ∼80% of S/G2 cells had a single Plk4 focus on the Cep152 torus (Figure 5C–F; Figure S9A, B).^17,41^

**Figure 6:**
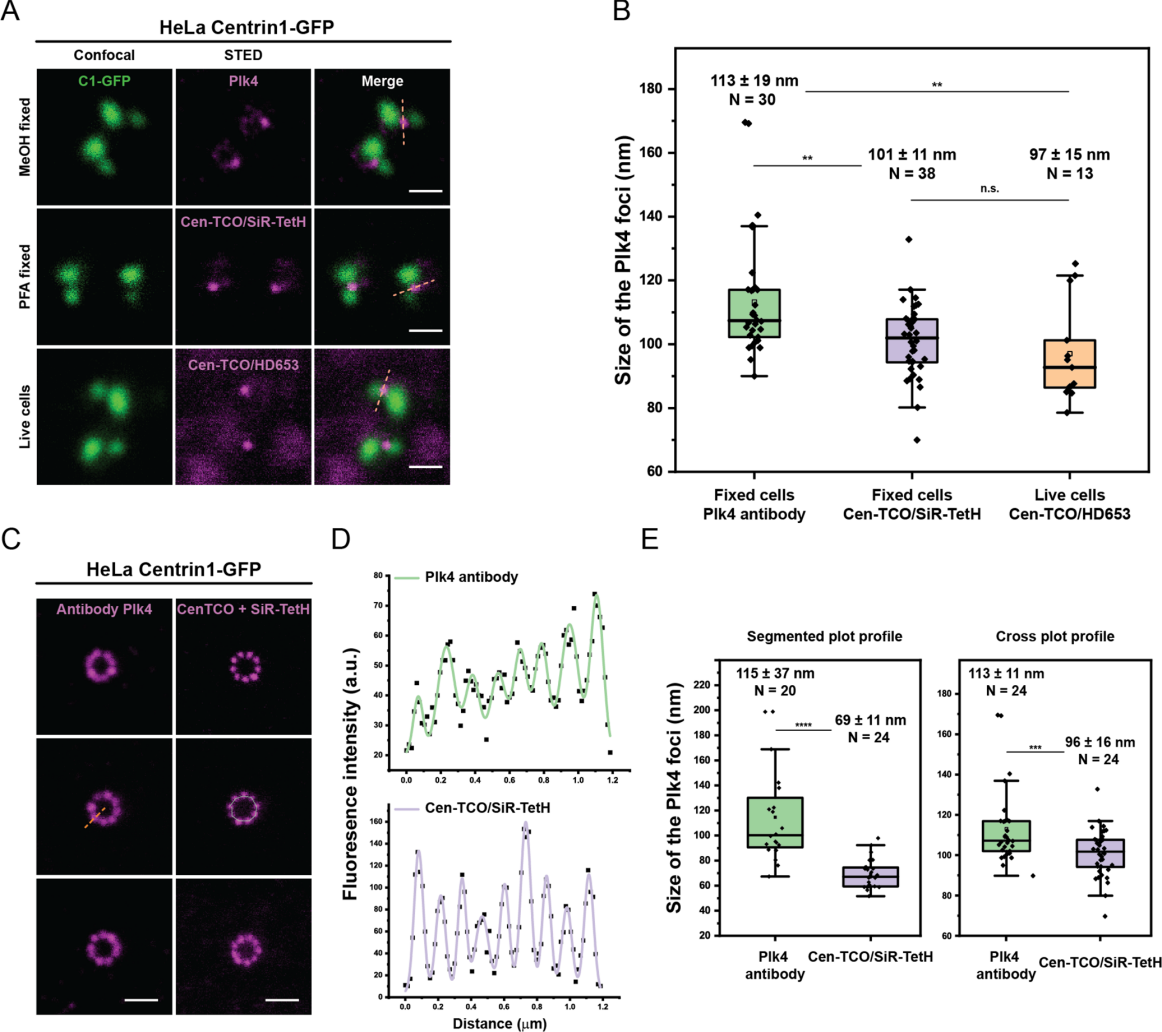
Plk4 foci size measurements. (A) Representative examples of HeLa cells expressing Centrin1-GFP incubated with 500 nM Cen-TCO (**3**) for 1 h followed by Plk4 labeling in different conditions. Upper panel: cells were fixed with −20 °C MeOH and labeled with mouse Plk4 primary antibodies in combination with antimouse Star 635P secondary antibodies; middle panel: cells were fixed with 2% PFA and labeled with SiR650-TetH (**4a**); lower panel: live cells imaging of Plk4 labeled with Cen-TCO/HD653. Scale bar: 500 nm. (B) The size (fwhm) comparison of the single Plk4 foci obtained from different labeling techniques shown in panel A). The fwhm of Plk4 foci is represented as mean ± s.d. N represents a number of samples obtained in multiple experiments. ** *p* value ≤0.01. n.s., not significant. (C) Representative examples of STED imaging of Plk4 accumulates obtained by 24 h incubation with Cen-TCO (**3**) followed by PFA fixation and labeling with left: primary Plk4 antibody and secondary. Abberior STAR 635P antibody; right: SiR650-TetH (**4a**). (D) Fluorescence intensity profile of the segmented green line in panel C, middle. Upper panel: plot profile obtained by labeling with antibody; lower panel: plot profile obtained by labeling with the Cen-TCO/SiR650-TetH system. (E) The size (fwhm) comparison of the single Plk4 foci obtained from different labeling techniques shown in panel C). Left: measurements obtained from segmented line passing in-between foci of 9-fold accumulated of Plk4. Right: Measurements obtained from the line drawn acros*s* of the circular signal of Plk4 accumulates. The fwhm of Plk4 foci are represented as mean ± s.d. N represents a number of measured foci. ****, *p* value ≤0.0001; ***, *p* ≤ 0.001. Represented STED images are not deconvoluted.

Immunostaining based on labeling with primary and secondary antibodies can increase the dimensions of the imaged structure by ∼20 nm. In addition, antibody epitopes on a target such as Plk4 might be distant from the active site. We therefore sought to utilize our newly gained ability to directly label Plk4 in super-resolution imaging to more precisely localize the active site. Specifically, we compared the fwhm of Plk4 foci obtained through antibody labeling^45^ with that achieved using Cen-TCO/SiR650-TetH. As shown in Figure 6A and 6B, we found that the latter revealed Plk4 foci of significantly smaller size in both fixed and live cells (Figure 6A). The increase in the resolution through chemical labeling was also apparent when imaging the accumulated Plk4 after inhibition with Cen-TCO (**3**) for 24 h (Figure 6C, 6D, and 6E)

Our work shows that for the low abundance protein Plk4, a two-step procedure is key for successful labeling and imaging at endogenous expression levels. This observation indicates that bio-orthogonal chemistry-based two-step labeling is a valuable alternative when traditional labeling with inhibitor-fluorophore conjugates is unsuccessful. We hypothesize that the smaller size of Cen-TCO relative to that of the corresponding SiR derivatives leads not only to higher affinity for Plk4, but also to improved cell permeability of the probe. Both effects might be important for the successful labeling of low abundance Plk4. Furthermore, this work underscores that an excellent background-to-signal ratio is essential for imaging low-abundance targets and that the use of bright and fluorogenic fluorophores such as silicon-based rhodamines is key to achieving that.

## Conclusions

We designed and synthesized novel probes for imaging Plk4 based on the potent inhibitor centrinone. The initial strategy of conjugating fluorogenic dyes to centrinone allowed the development of SiR595-centrinone (**2d**), which was successfully used to reveal the 9-fold symmetrical arrangement of overexpressed Plk4 in live-cell STED imaging. However, SiR595-centrinone (**2d**) was not able to detect endogenous Plk4, presumably due to its low expression levels in HeLa cells and the low affinity of the probe. To circumvent these problems, we utilized a two-step labeling strategy based on tetrazine bio-orthogonal chemistry. We demonstrated that Cen-TCO (**3**) together with fluorogenic HD653 (**5**) can reveal endogenous Plk4 during live-cell STED imaging, something that has not been previously achievable. Furthermore, we demonstrated that such an approach can be utilized in fixed cells to image Plk4 in different cell cycle stages. Our experiments also showed that the use of small molecule labeling leads to a more precise localization in super-resolution imaging of Plk4, due to direct binding to the active site and minimal probe size. However, the use of inhibitors to label proteins of interest inherently interferes with their biological activity. We anticipate that the identification of new, noninhibitory, effective binders (e.g., small molecules that bind to a surface pocket of a protein) will lead to the development of less disruptive labeling probes for the observation of biological processes. The two-step labeling approach demonstrated in this work can be highly beneficial for probes with limited cellular permeability. Nevertheless, targeting low abundance proteins such as Plk4 requires molecules such as centrinone that possess high affinity and high selectivity; such molecules are typically scarce. Finally, we anticipate that our probes will be of importance in future studies of Plk4 and centrioles.

## Experimental Section

### Live-Cell Labeling of HeLa Cells by SiR595-Centrinone

Live-cell staining with SiR595-probes was achieved by adding the probes from a 1 mM DMSO stock solution to the complete growth medium to obtain the desired final concentration (usually 200–1000 nM) and incubating for 1 h in a humidified 5% CO_2_ incubator at 37 °C. If required, Hoechst 33342 was added together with probes (5–15 min) at the final concentration of 1 μg/mL

### Staining of Plk4 in Living Cells with Cen-TCO/HD653 Labeling System

HeLa cells expressing Centrin1-GFP were incubated with 500 nM Cen-TCO for 1 h in a humidified 5% CO_2_ incubator at 37 °C, followed by a brief 15 min incubation with 500 nM HD653. Cells were imaged on confocal and STED microscopes without prior washing. We found that performing additional washing steps prior to imaging did not improve the background signal and induced rapid overamplification phenotype.

For imaging of Plk4 accumulations, HeLa cells expressing Centrin1-GFP cells were incubated with 500 nM Cen-TCO for 24 h, followed by a 15 min incubation with 500 nM HD653. Cells were imaged on confocal and STED microscopes with or without prior washing, as indicated in the text.

### Staining of Plk4 in Fixed Cells

Hela and U2OS cells were seeded on sets of noncoated coverslips placed in six-well culture plates. Cells were let to attach on coverslips overnight in a humidified 5% CO_2_ incubator at 37 °C. Cells were then incubated with the desired amounts of Cen-TCO (20–1000 nM) for 1 h (or 2–24 h). PFA fixation was performed by adding 2% PFA in growth medium for 10 min at room temperatures and then washed twice with PBS. Methanol fixation was performed as follows: growth medium was removed from cells; cells were incubated for 3–5 min in −20 °C cold methanol and washed three times with PBS. Upon fixation, cells were permeabilized and blocked with PBST (0.05% triton, 1% BSA in PBS) for 30 min. Labeling with primary antibodies was performed for 4 h at room temperature or overnight at 4 °C. To the primary antibody solution, SiR650-TetH was added to the final concentration of 200–400 nM. Note that it is sufficient to incubate SiR650-TetH for 1 h in conditions without antibody labeling. Coverslips were washed trice with PBST with 5 min incubations. Secondary antibody labeling was performed for 1 h, after which coverslips were washed three times with PBST and mounted on slides for imaging.

The following primary antibodies were used: Rabbit polyclonal antibodies against Cep152 (Bethyl Laboratories, A302–480A, IF 1:1000), and STIL (Abcam, ab89314, IF 1:500); mouse monoclonal antibodies against Plk4 (Merck Millipore, clone 6H5, MABC544, IF 1:500), and HsSAS-6 (Santa Cruz Biotechnology, Inc., sc-81431, IF 1:500). The following secondary antibodies were used: Alexa Fluor 488 goat antimouse IgG (H + L) (Molecular Probes, A11001, IF 1:1000), Alexa Fluor 488 goat antirabbit IgG (H + L) (Molecular Probes, A11008, IF 1:1000), Abberior STAR 580 goat antirabbit (SIGMA, 41367, 1:500), Abberior STAR 580 goat antimouse (SIGMA, 52403, 1:500), Abberior STAR 635P goat antimouse (SIGMA, 40734, 1:500).

## Supporting Information Available

Detailed experimental procedures, material and methods, synthesis of SiR595-centrinone, Cen-TCO, and other compounds; characterization, original ^1^H and ^13^C NMR spectra, absorption and emission spectra, procedures for cell culture, detailed procedure for labeling and imaging of Plk4, binding affinity and inhibition assay procedures, centriole scoring data procedures, and microscopy setting information (PDF)
