# Supplementary material for: Chemical Probe for Imaging of Polo-like Kinase 4 and Centrioles
Source: JACS Au. 2023 Aug 4;3(8):2247–56. doi: 10.1021/jacsau.3c00271 (PMC10466336; doi:10.1021/jacsau.3c00271)
Supplement: Supplementary file 1 — au3c00271_si_001.pdf [file au3c00271_si_001.pdf]

Supplementary material

Chemical probe for imaging polo-like kinase 4 and centrioles

Aleksandar Salim[1,2], Philip Werther[3], Georgios N. Hatzopoulos[4], Luc Reymond[2], Richard Wombacher[1,3], Pierre Gönczy[4*], Kai Johnsson[1,2*]

[1] Department of Chemical Biology, Max Planck Institute for Medical Research, Jahnstrasse 29, 69120 Heidelberg, Germany

[2] Institute of Chemical Sciences and Engineering (ISIC), École Polytechnique Fédérale de Lausanne (EPFL), 1015 Lausanne, Switzerland.

[3] Institute of Pharmacy and Molecular Biotechnology, Heidelberg University, Im Neuenheimer Feld 364, 69120 Heidelberg, Germany

[4] Swiss Institute for Experimental Cancer Research (ISREC), School of Life Sciences, Swiss Federal Institute of Technology (EPFL), 1015 Lausanne, Switzerland

*Correspondence: johnsson@mr.mpg.de ; pierre.Gönczy@epfl.ch

1.1 Supplementary figures

A

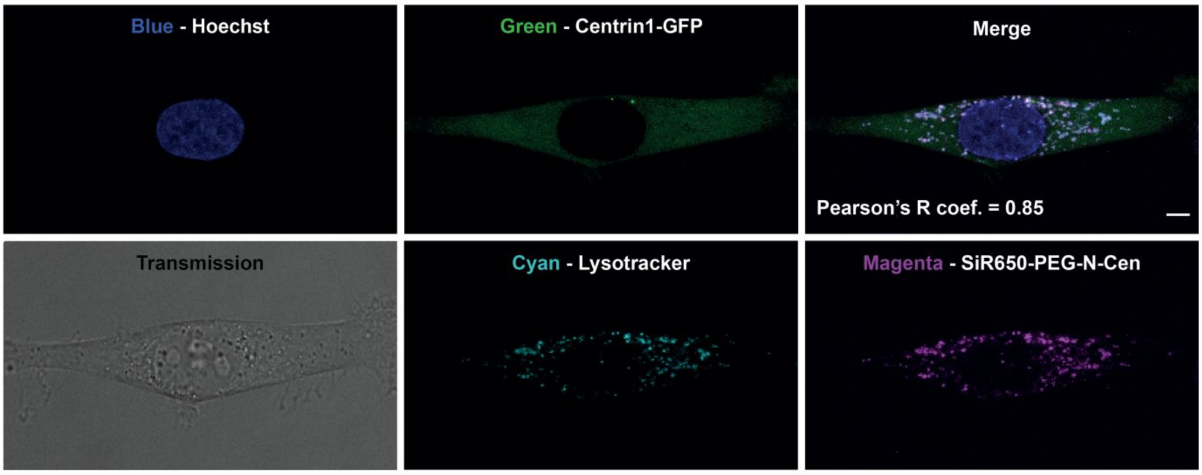

B

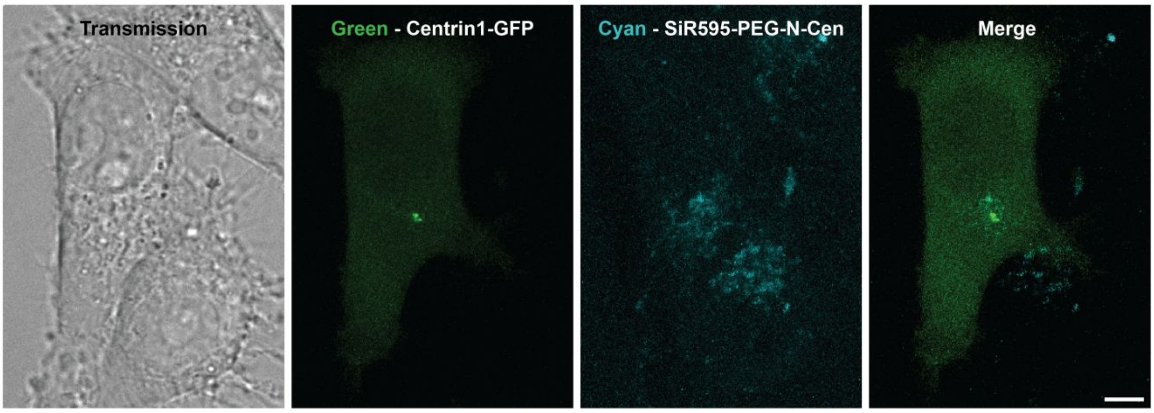

Figure S1. Accumulation of SiR650-PEG-N-Cen & SiR595-PEG-N-Cen probe in lysosomes/endosomes. A) Live confocal images of HeLa cells stably expressing Centrin1-GFP incubated for 1 h with SiR650-PEG-N-Cen (1 µM), 30 min with Lysotracker (50 nM) and 10 min with Hoechst 33342. Images indicate probe accumulation in lysosomes/endosomes. B) Live-confocal images of HeLa cells stably expressing Centrin1-GFP incubated for 24 h with SiR595-PEG-N-Cen (1 µM). The SiR595 channel shows distinct point like signals that most likely represent endolysosomal accumulation. We have not investigated this signal further. Scale bar: 5 µm.

A

Silicon-Rhodamine 595

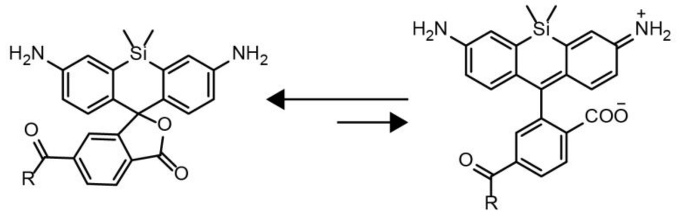

Spirolactone equilibrium

B

	λ_{max} abs (nm)	λ_{max} em (nm)	E_{max} (M^{-1} cm^{-1})[1]	D_{50} [2]	τ (ns)[3]	QY[1]
**SiR595**	595	620	75000	0	3.2/3.3/3.5	0.5
**SiR650**	652	667	120000	59	2.6/3.0/3.2	0.4

[1] E_{max} was measured in ethanol in presence of 0.1 % TFA; [2] Dioxane titrations were performed with 3 μM solution of the dye
[3] Fluorescence lifetime measurements of 200 nM dye solutions were made in TBS / TBS with 0.1 % SDS/ EtOH with 0.1 % TFA, respectively.

C

Excitation & Emission spectra of SiR595 & SiR650

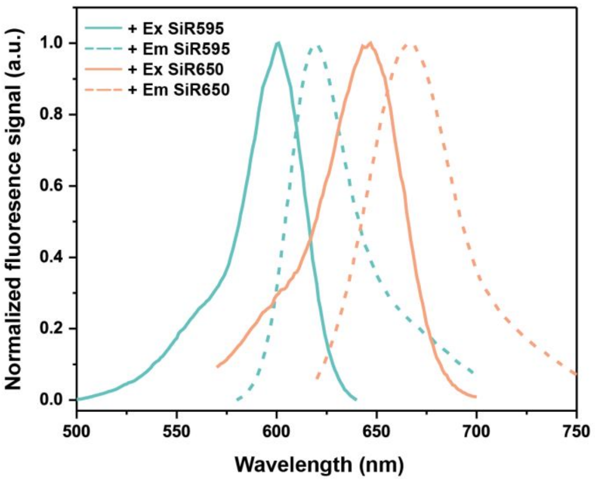

D

Dioxane titrations of SiR650 and SiR595

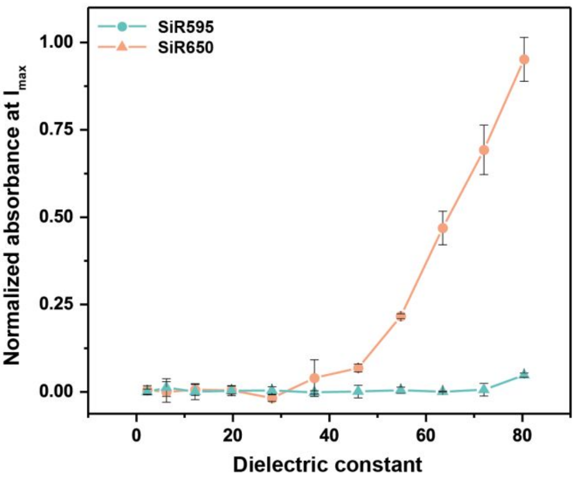

Figure S2. Spectral and fluorogenic properties of SiR595. **A**) Spirolactone equilibrium of SiR595. **B**) Table of photophysical characterization of SiR595 and SiR650. **C**) Extinction and emission spectra of SiR595. **D**) Dioxane titrations of SiR595 and SiR650.

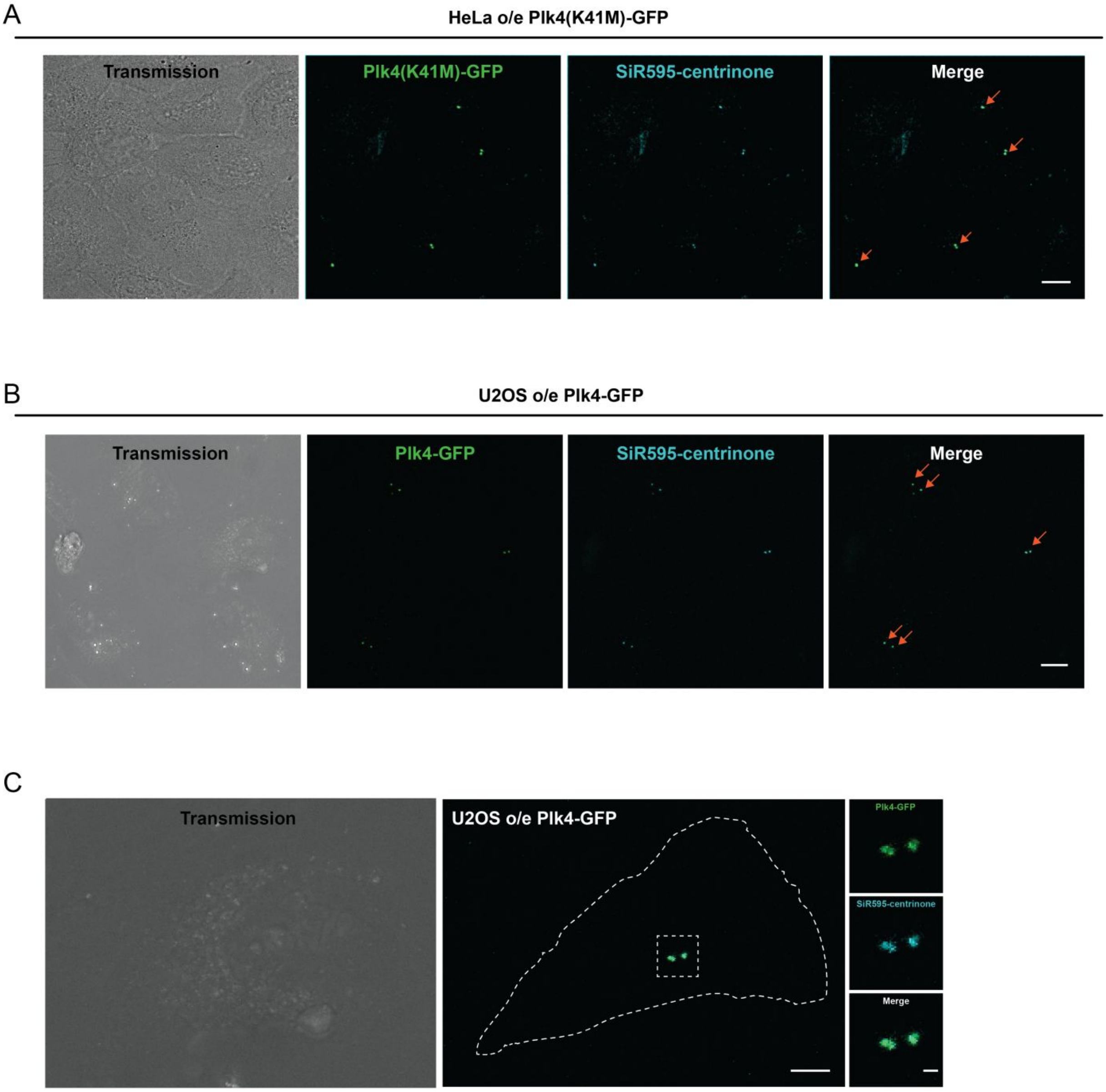

Figure S3. Live-cell confocal imaging with SiR595-centrinone. A**-& B)** Low magnification live-cell confocal imaging of HeLa (A) and U2OS (B) cells transfected with (A) GFP-Plk4(K41M)- or (B) Plk4-GFP, followed by labeling with SiR595-C2-C-Cen (500 nM). Scale bar: 10 µm; **C**) Live-cell confocal imaging of U2OS cells transfected with GFP-Plk4, followed by labeling of SiR595-C2-C-Cen (500 nM). Scale bar: 5 µm (A, B); 1 µm (C).

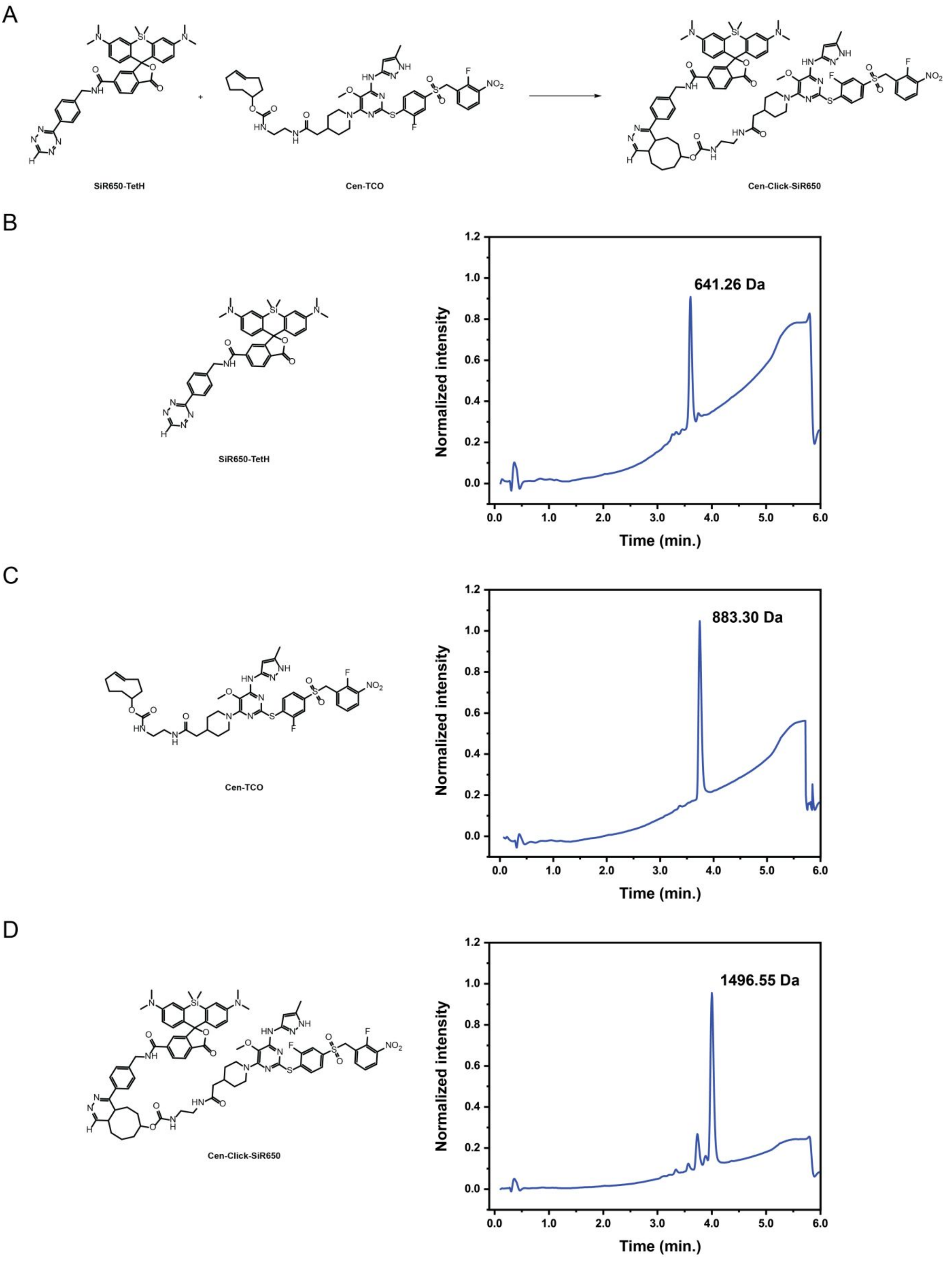

Figure S4. A) Scheme of click reaction between SiR650-TetH (**4a**) and Cen-TCO (**3**). **B- C**) LC-MS chromatograms of pure compounds SiR650-TetH (**4a**) and Cen-TCO (**3**), respectively. Chromatogram is represented as UV absorbance trace at 254 nm. **D**) LC-MS chromatograms of 20 µM SiR650-TetH (**4a**) was 50 µM Cen-TCO (**3**) mixture recorded after 30 min. of incubation. Chromatogram is represented as UV absorbance trace at 254 nm.

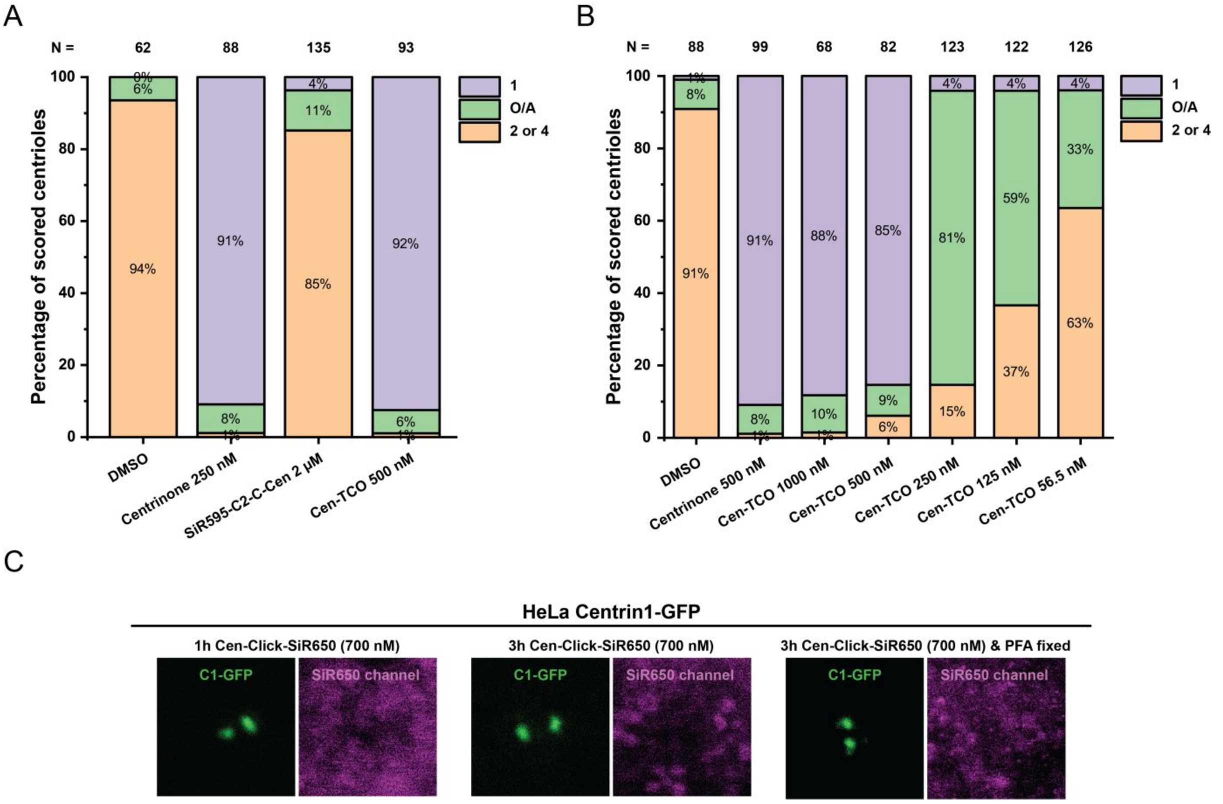

Figure S5. Phenotypic assessment of developed probes & assessment of Cen-Click-SiR650 for labeling of Plk4. **A**) Scoring of Centrin1-GFP foci in HeLa cells incubated with different centrinone based probes. Cells with one Centrin1-GFP focus exhibit an under-duplication phenotype, cells with multiple Centrin1-GFP foci an over-amplification phenotype. Cells with 2 or 4 Centrin1-GFP have a normal number. **B**) Scoring of Centrin1-GFP foci in HeLa cells incubated with different concentrations of Cen-TCO. **C)** Live-cell imaging of HeLa cells expressing Centrin1-GFP as a centriolar marker incubated with Cen-Click-SiR650 (700 nM) for 1h & 3h (left and middle) as well as images upon PFA fixation following incubation with 700 nM Cen-Click-SiR650 for 3h (right). Images show no co-localization of GFP and SiR650 signals

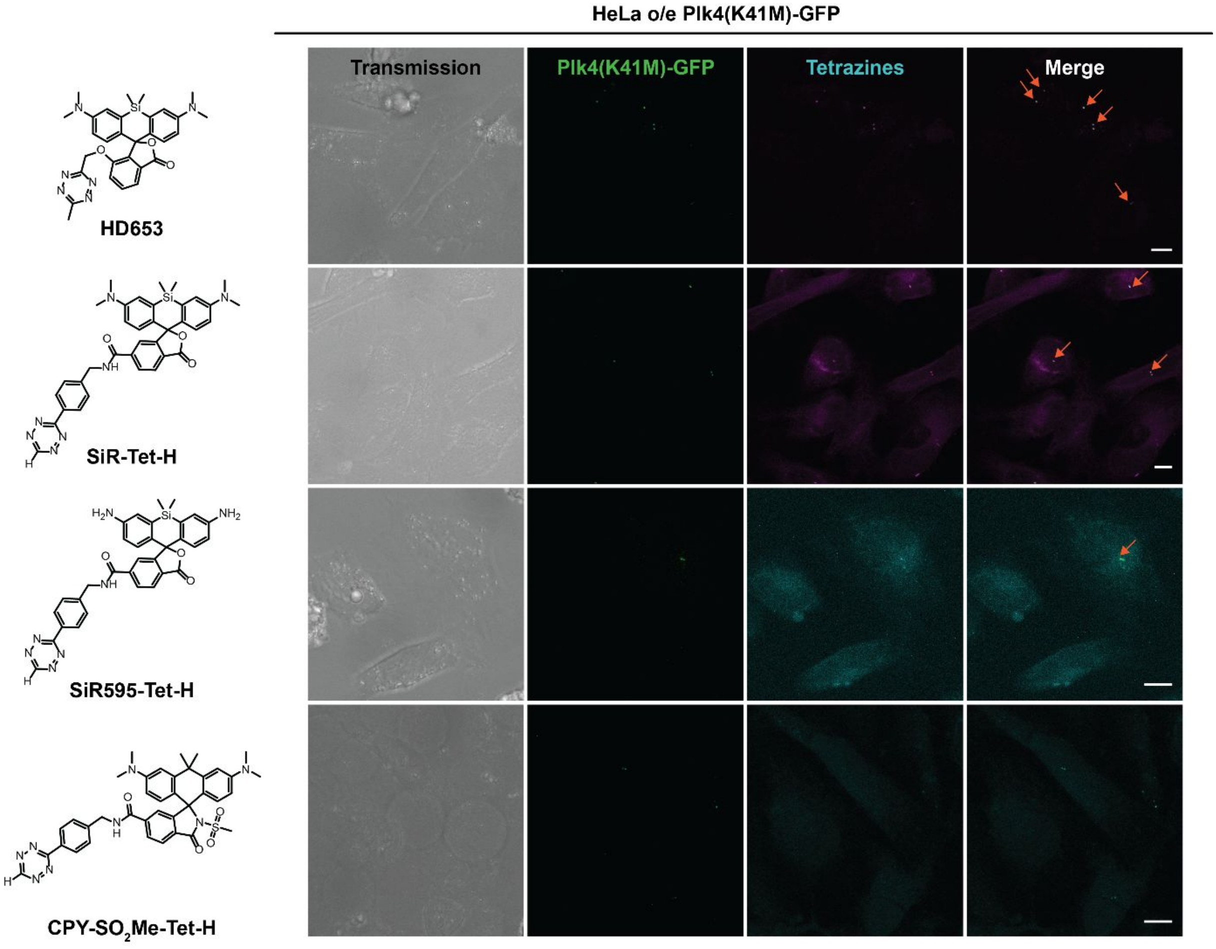

Figure S6. Various tetrazines are tested for labeling of overexpressed Plk4(K41)-GFP. Live-cell confocal imaging of HeLa cells transfected with Plk4(K41)-GFP incubated with Cen-TCO (500 nM) for 1 h, followed by 1 h incubation of different tetrazines (500 nM). Cells were imaged without washing. Scale bar: 10 µm. Orange arrows in the merged images indicate colocalization of centriolar signal from GFP and the respective tetrazine-fluorophores.

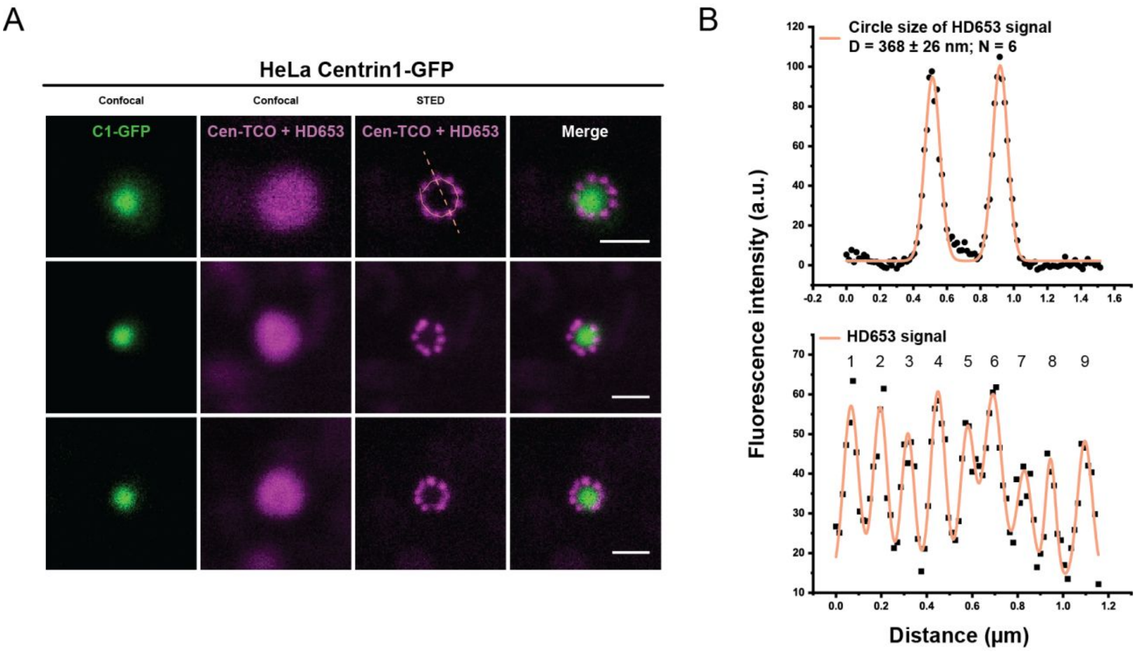

Figure S7. Live-cell STED imaging of Plk4 accumulation. **A)** Live-cell STED images of HeLa cells expressing Centrin1-GFP incubated with Cen-TCO (500 nM) for 24 h, followed by 15 min incubation with HD653 (500 nM). Cells were imaged after a brief wash with media. Scale bar: 500 nm. **B**) Upper panel: fluorescence intensity profile representation of the orange line in panel (A). The size of the diameter of Plk4 accumulations labelled with Cen-TCO & HD653 is represented as mean ± s.d. Lower panel: fluorescence intensity profile representation of circular segmented line in panel (A) representing nine-fold symmetrical arrangement of Plk4 accumulates.

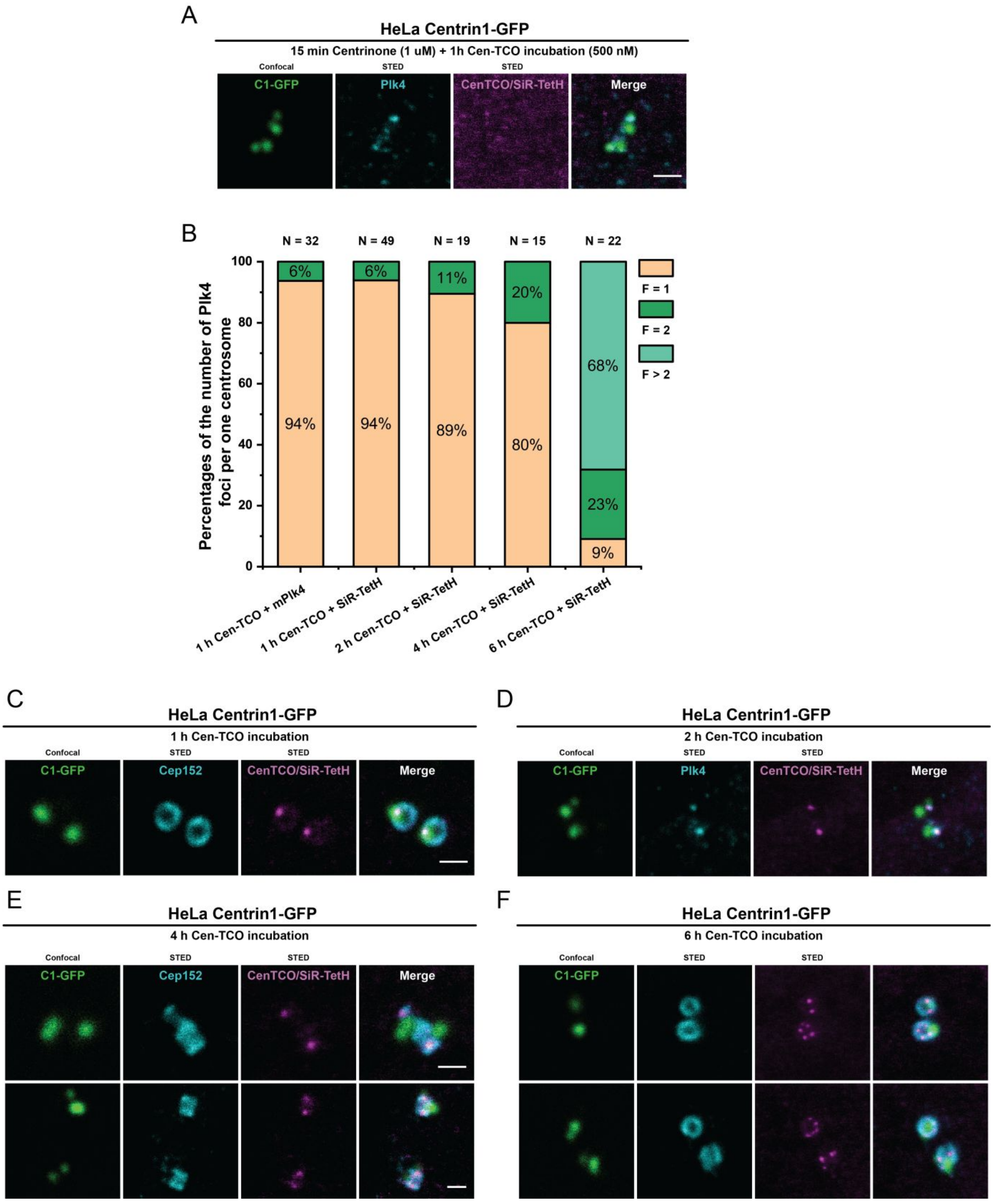

Figure S8. Effects of long-term Cen-TCO incubation on Plk4 localization**. A**) Confocal and STED imaging of HeLa cells expressing Centrin1-GFP incubated with centrinone (1 µM) for 15 min, followed by 1h Cen-TCO (500 nM). After fixation with 2% PFA, the sample was stained with SiR650-TetH (200 nM) and Plk4 antibodies. Scale bar: 500nm; **B**) Scoring of the Plk4 foci number per centriole at different time points of Cen-TCO (500 nM) incubation in HeLa cells expressing Centrin1-GFP as centriolar marker. **C-F**) Representative confocal and STED imaging of Hela cells expressing Centrin1-GFP labelled with Cen-TCO (500 nM) and Cep152/Plk4 antibodies at different time points. Scale bar: 500nm.

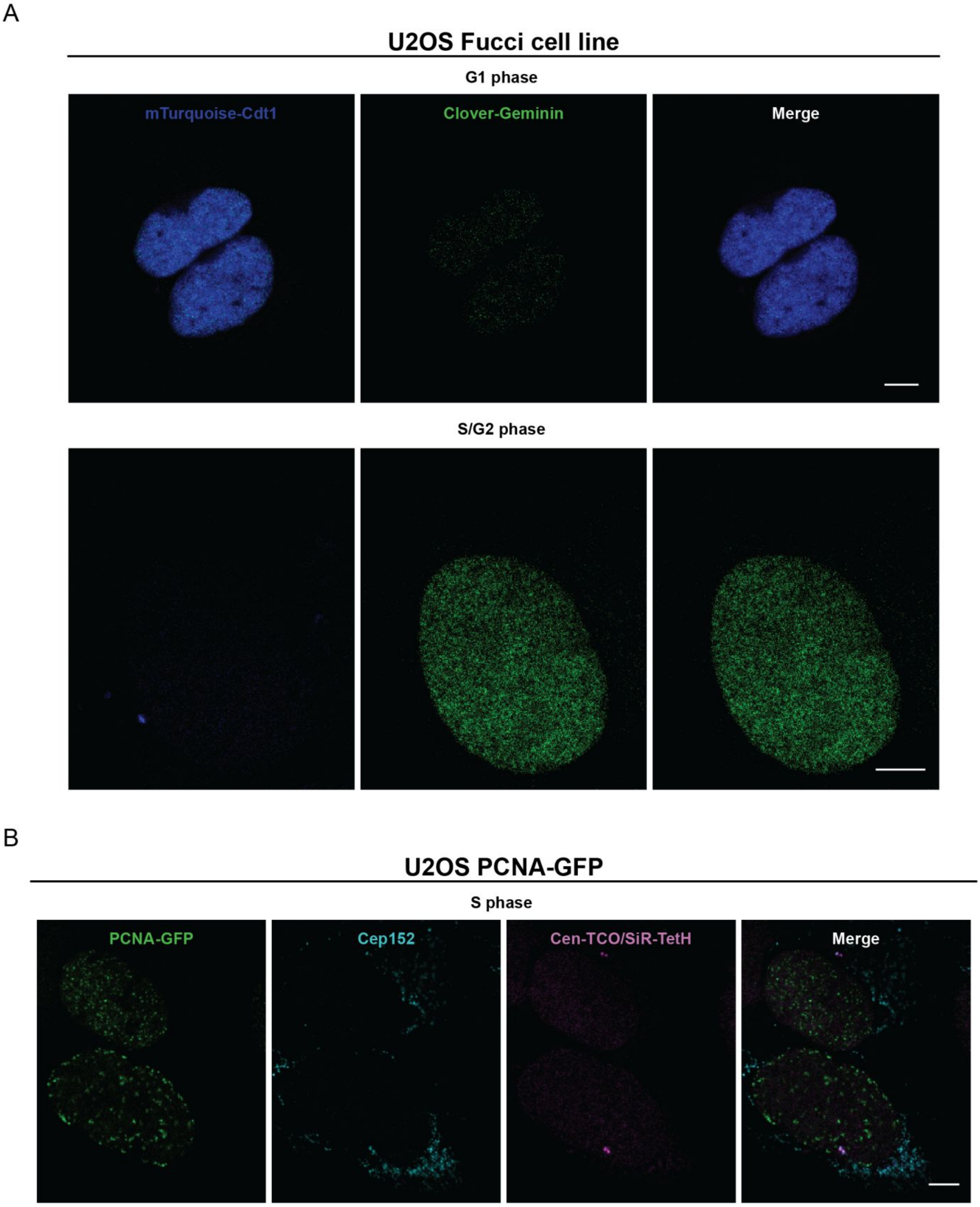

Figure S9. A) Confocal imaging of U2OS cells expressing mTurquoise-Cdt1 as a G1 cell cycle phase marker and Clover-Geminin as a S/G2 phase cell cycle marker **B**) Confocal imaging of U2OS cells expressing PCNA-GFP as a S cell cycle phase marker, with Plk4 labelled with Cen-TCO (500 nM) for 2h, followed by PFA fixation and incubation with SiR650-TetH (200nM) together with Cep152 antibody immunostaining.

Material and Methods

1.2 General considerations

All chemical reagents and anhydrous solvents for synthesis were purchased from commercial suppliers (Acros, Apollo, Armar, Bachchem, Biomatrik, Carbosynth, Fluka, Fluorochem, LC Laboratories, Merck, Reseachem, Roth, Sigma-Aldrich and TCI) and used without further purification. Composition of mixed solvents is given by volume ratio (v/v). Reactions in the absence of air and moisture were performed in oven-dried glassware under Ar or N2 atmosphere. Flash column chromatography was performed using a Biotage (Isolera™) flash system using SiliaSep™ columns. The used solvent compositions are reported individually in parentheses. Analytical thin layer chromatography was performed on glass plates coated with silica gel 60 F254 (Merck). Visualization was achieved using UV light (254 nm). Evaporation in vacuo was performed at 25–60 °C and 900–10 mbar. ^{1}H, ^{13}C, and ^{19}F NMR spectra were recorded on AV 400, Ascend™ 400 and AV 600 Bruker spectrometers at 400 MHz (1H), 101 MHz (^{13}C) and 377 MHz (^{19}F), respectively. All spectra were recorded at 298 K. Chemical shifts δ are reported in ppm downfield from tetramethylsilane using the residual deuterated solvent signals as an internal reference (CDCl3: δ H = 7.26 ppm, δ C = 77.16 ppm; CD3OD: δ H = 3.31 ppm, δ C = 49.00 ppm; DMSO-d6: δ H = 2.50 ppm, δ C = 39.52 ppm). For 1H, 13C and 19F NMR, coupling constants J are given in Hz and the resonance multiplicity is described as s (singlet), d (doublet), t (triplet), q (quartet), quint (quintet), sext (sextet), sept (septet), m (multiplet) and br. (broad). High-resolution mass spectrometry (HRMS) was performed by the MS-service of the EPF Lausanne (SSMI) on a Waters Xevo® G2-S Q-Tof spectrometer with electron spray ionization (ESI) or by the MS-facility of the Max Planck Institute for Medical Research on a Bruker maXis IITM ETD. Liquid chromatography coupled to mass spectrometry (LC-MS) was performed on a Shimadzu MS2020 connected to a Nexera UHPLC system equipped with a Waters ACQUITY UPLC BEH C18 (1.7 µm, 2.1 x 50 mm) column or a Supelco Titan C18 80 Å (1.9 µm, 2.1 x 50 mm). Buffer A: 0.05% HCOOH in H2O Buffer B: 0.05% HCOOH in ACN. Analytical gradient was from 10% to 90% B within 6 min with 0.5 mL/min flow unless otherwise stated. Preparative reverse phase high-performance liquid chromatography (RP-HPLC) was carried out on a Dionex system equipped with an UltiMate 3000 diode array detector for product visualization on a Waters Symmetry C18 column (5 µm, 3.9 x 150 mm), Waters SunFireTM Prep C18 OBDTM (5 µm, 10 x 150 mm) column, Supleco Ascentis® C18 column (5 µm, 10 x 250 mm) or on a Supleco Ascentis® C18 column (5 µm, 21.2 x 250 mm). Buffer A: 0.1% TFA in H2O Buffer B: ACN. Typical gradient was from 10% to 90% B within 32 min with 2, 4 or 8 mL/min flow.

1.3 Protocols

1.3.1 Extinction coefficient

SiR595-6'-COOH was dissolved in DMSO-*d6* and 1,2-dichloroethane (DCE) was added to a final concentration of 36.1 mM. The concentration of SiR595 was determined by ^{1}H-NMR using dichloroethane as internal standard and the solution was used to obtain a series of dilutions in EtOH + 0.1 % TFA. Absorbance was measured by UV-Vis spectrometry (JASCO V770 spectrophotometer) d from 500 nm to 750 nm. E_{max} was calculated using Lamberts-Beer law.

1.3.2 UV-Vis measurements

Solution of 5 µM substrate in TBS (20 mM Tris-HCl pH 7.4, 100 mM NaCl), TBS containing 1 mg/mL BSA, TBS containing 0.1 % SDS, ethanol or ethanol containing 0.1 % TFA were prepared. Absorbance

spectra were recorded using a JASCO V770 spectrophotometer and small 1 cm quartz cuvettes (Hellma Cuvettes) with volume of 125 µL.

1.3.3 Quantum yields measurements

A solution of 200 nM SiR595-6'-COOH was prepared in EtOH + 0.1 % TFA. Quantum yields were determined using a Hamamatsu Quantaurus QY.

1.3.4 Dioxane titration

Solutions of 10 µM SiR595-6'-COOH and SiR650-COOH were prepared in water-dioxane mixtures containing 0 %, 10 %, 20 %, 30 %, 40 %, 50 %, 60 %, 70 %, 80 %, 90 % and 100 % of dioxane (v/v). The absorbance spectra were recorded using a plate reader (TECAN Spark® 20M) using polypropylene Greiner 96-well pates in 1 nm steps. The normalized absorbance at λ_{max} was plotted against the dielectric constant of dioxane/water mixtures and when permitted the inflection point was determined by using sigmoidal fitting in Originlab 8.1.[1]

1.3.5 Estimation of fluorescence increase

The probes were analysed as described in Lukinavičius et al. 2014[1]. Briefly, SiR650 or SiR595-Centrinone probe from a 1 mM DMSO (Applichem) stock solution was directly added to a solution of purified Plk4 kinase domain (10 µM in 20 mM Tris, 200 mM NaCl at pH 7.5) or to a bovine serum albumin (BSA) (Sigma) solution (0.2 mg/mL in 20 mM Tris, 200 mM NaCl at pH 7.5). Final concentration of the probe was 3 µM. The samples were incubated for 1 h at 37 °C, and fluorescence was measured in a 96-well plate (Greiner Bio-One) on a plate reader (TECAN Spark® 20M). Fluorescence emission was recorded from 570 nm to 750 nm for SiR595 probes, and 630 nm to 750 nm for SiR650 while exciting at 550 nm ± 10 nm for SiR595 probes and at 610 ± 10 nm for SiR650 probes. A fluorescence excitation scan was recorded by measuring the emission at 660 ± 10 nm for SiR595 and 720 ± 10 nm for SiR650 probes, while exciting from 500 nm to 640 nm for SiR595 probes and from 580 nm to 700 nm for SiR650 probes. All samples were prepared in triplicates. Ratios F(+target)/F(+BSA) of fluorescence signals were measured at 620 nm for SiR595 probes and 670 nm for SiR650 probes.

1.3.6 Fluorescence Polarization assay & competition assay

A solution of 1 nM Alexa488-PEG-N-Centrinone was titrated with increasing concentrations of Plk4-KD dissolved in 20 mM Tris pH 8.0, 200 mM NaCl, 0.2 mg/mL BSA in a black flat bottom 96-well plate (Greiner Bio-One). Upon mixture of protein and ligand, the plate was incubated for 30 min at room temperature followed by fluorescence polarization measurements by a plate reader (TECAN Spark® 20M) using 485 nm as excitation wavelength (bandwidth 20 nm) and 535 nm as emission wavelength (bandwidth 25 nm). The obtained fluorescence polarization values from each well were plotted against the concentration of the protein. The apparent K_d values were obtained by fitting the curve to the following equation[2-3]:

$$FP = FP_{min} + \left(\frac{FP_{max} - FP_{min}}{2C_f}\right)\left(C_f + C_p + K_d - \sqrt{(C_f + C_p + K_d)^2 - 4C_fC_p}\right)$$

where FP_{min} is the minimum polarization value, FP_{max} is the maximum polarization value, C_f is the concentration of Alexa488-Centrinone and C_p is the concentration of the protein(Plk4-KD).

For the competition assay, we used 1 nM Alexa488-Centrinone and 5 nM Plk4-KD. The amount of protein should be around the K_d of the tracer to ensure that enough free protein that can bind the

competitor, and enough signal change coming from binding and unbinding of the tracer. The solution of the tracer and protein was titrated with increasing amounts of competitors, in our case SiR650- and SiR595-Centrinone probes. The mixture was then incubated for an hour at room temperature and the FP was then measured on a plate reader using the same conditions as mentioned before. From the obtained FP values, we have calculated the amount of free protein in the solution for each concentration of competitor using the equation of the single-site binding isotherm:

$$[P_{free}] = K_{d\_tracer} \frac{FP_{min} - FP}{FP - FP_{max}}$$

Where P_{free} is concentration of free protein, $K_{d\ tracer}$ represents the previously determined binding affinity of the tracer, FP_{min} is the minimum polarization value, FP_{max} is the maximum polarization value.

The obtained concentration of free protein was then fitted against the concentration of competitor and the obtained curve fitted by single site binding isotherm to determine the K_d of the protein towards the competitor:

$$FP = FP_{min} + \frac{FP_{max} - FP_{min}}{1 + \frac{K_{d,\ competitor}}{[Protein]}}$$

1.3.7 Cell Culture and Transfection

HeLa (ATCC), HeLa::Centrin1-GFP cells (Piel et al, 2000), U2OS (ATCC) cells were cultured in high-glucose phenol red free DMEM (Life Technologies) medium supplemented with GlutaMAX (Life Technologies), sodium pyruvate (Life Technologies) and 10% FBS (Life Technologies) in a humidified 5% CO_2 incubator at 37 °C. Cells were split every 3–4 days or at confluency. The cell lines were regularly tested for mycoplasma contamination. Cells were seeded on glass bottom 35 mm dishes (Mattek or Greiner Bio-One), 10 well glass bottom dishes (Greiner Bio-One) or on 12 mm coverslips one day before imaging. Transient transfection of cells was performed using Lipofectamine™ 2000 reagent (Life Technologies) according to the manufacturer's recommendations: 2.5µg of DNA was mixed with OptiMEM (100 µL, Life Technologies) and Lipofectamine™ 2000 (6 µL) was mixed with OptiMEM (100 µL). The solutions were incubated for 5 min at room temperature, then mixed and incubated for an additional 10 min at room temperature. The prepared DNA-Lipofectamine complex was added to a glass bottom 10-well glass bottom dishes with cells at 50–70% confluency. After 12 h incubation in a humidified 5% CO2 incubator at 37 °C the medium was changed to fresh medium. The cells were recovered in fresh media for 4 h before adding probes for imaging.

1.3.8 Staining of living cells with SiR595-centrinone probes

Live-cell staining with SiR595-probes was achieved by adding the probes from a 1 mM DMSO stock solution to the complete growth medium to obtain the desired final concentration (usually 200-1000 nM) and incubating for 1 h in a humidified 5% CO_2 incubator at 37 °C. If required, Hoechst 33342 was added together with probes (5-15 min) at the final concentration of 1 µg/ml.

1.3.9 Staining of Plk4 in living cells with Cen-TCO/HD653 labeling system

HeLa cells expressing Centrin1-GFP were incubated with 500 nM Cen-TCO for 1 h in a humidified 5% CO_2 incubator at 37 °C, followed by a brief 15 min incubation with 500 nM HD653. Cells were imaged on confocal and STED microscopes without prior washing. We found that performing additional washing steps prior to imaging did not improve the background signal and induced rapid over-amplification phenotype.

For imaging of Plk4 accumulations, HeLa cells expressing Centrin1-GFP cells were incubated with 500 nM Cen-TCO for 24 h followed by a 15 min incubation with 500 nM HD653. Cells were imaged on confocal and STED microscopes with or without prior washing, as indicated in the text.

1.3.10 Staining of Plk4 in fixed cells

Hela and U2OS cells were seeded on sets of non-coated coverslips placed in six-well culture plates. Cells were let to attach on coverslips overnight in a humidified 5% CO_2 incubator at 37 °C. Cells were then incubated with the desired amounts of Cen-TCO (20-1000 nM) for 1h (or 2h-24h). PFA fixation was performed by adding 2% PFA in growth medium for 10 min at room temperatures and then washed twice with PBS. Methanol fixation was performed as follows: growth medium was removed from cells, cells were incubated for 3–5min in −20 °C cold methanol and washed three times with PBS. Upon fixation, cells were permeabilized and blocked with PBST (0.05% triton, 1% BSA in PBS) for 30 min. Labeling with primary antibodies was performed for 4 h at room temperature or overnight at 4 ˚C. To the primary antibody solution SiR650-TetH was added to the final concentration of 200-400 nM. Note that it is sufficient to incubate SiR650-TetH for 1 h in conditions without antibody labeling. Coverslips were washed trice with PBST with 5 min incubations. Secondary antibody labeling was performed for 1 h after which coverslips were washed three times with PBST and mounted on slides for imaging.

The following primary antibodies were used: Rabbit polyclonal antibodies against Cep152 (Bethyl Laboratories, A302–480A, IF 1:1000), and STIL (Abcam, ab89314, IF 1:500); mouse monoclonal antibodies against Plk4 (Merck Millipore, clone 6H5, MABC544, IF 1:500), and HsSAS-6 (Santa Cruz Bio-technology, Inc., sc-81431, IF 1:500). The following secondary antibodies were used: Alexa Fluor 488 goat anti-mouse IgG (H + L) (Molecular Probes, A11001, IF 1:1000), Alexa Fluor 488 goat anti-rabbit IgG (H + L) (Molecular Probes, A11008, IF 1:1000), Abberior STAR 580 goat anti-rabbit (SIGMA, 41367, 1:500), Abberior STAR 580 goat anti-mouse (SIGMA, 52403, 1:500), Abberior STAR 635P goat anti-mouse (SIGMA, 40734, 1:500).

1.3.11 Centriole phenotypic scoring

HeLa::Centrin1-GFP previously seeded in 96-well plate were incubated with different concentrations of Cen-TCO for 24h in a humidified 5% CO_2 incubator at 37 °C. Upon incubation, cells were fixed with cold methanol (−20 °C for 3-5 min), washed with PBS and imaged on a confocal microscope. Scoring of centrioles was performed using a 63x objective and using the eye piece of the confocal microscope, counting the number of GFP foci per cell.

1.3.12 Plk4 foci scoring

HeLa::Centrin1-GFP, U2OS::PCNA-GFP or U2OS::Fucci cells were seeded on non-coated coverslips and let in incubator overnight. The cells were incubated with 500 nM Cen-TCO for 1h (HeLa cells) or 2h (U2OS cells) in warm media in a humidified 5% CO_2 incubator at 37 °C. Cells were imaged at the STED microscope and each clearly observed focus of labelled Plk4 was imaged and counted. When using U2-OS cell lines marking cell cycle stage, prior to imaging of centrioles, the nucleus was imaged to determine the cell cycle stage.

1.3.13 Expression, purification and characterization of Plk4-KD-6xHis

Plasmids encoding fragments of Plk4 kinase domain (2-275) with 6xHis-tag were cloned in pET system vectors. The recombinant protein expression of the fragments was performed in *E. coli* strain BL21 gold (DE3) in LB medium. Protein expression was induced at 18 °C by addition of 0.3 mM IPTG and

expression allowed to proceed for 18 h. Bacteria pellets were resuspended in lysis buffer containing 50 mM Tris (pH7.5), 400 mM NaCl, 2 mM $MgCl_2$, 5 mM EDTA, 1 mM DTT, 0.5 mM PMSF, 5% glycerol, 0.5% TritonX-100 and lysed by lysozyme treatment and sonication. The lysate was centrifuged for 45 min at 4 °C at 10000 rpm and the supernatant loaded on a 5mL HisTrap HP (GElifesciences) column using an Akta system (Cytiva). The column was washed (20mM Tris (pH 7.5), 1 mM DTT, 800 mM NaCl, 10% glycerol, 40 mM imidazole) and the protein finally eluted in elution buffer (20 mM Tris (pH 7.5), 1 mM DTT, 400 mM NaCl, 10% glycerol, 500 mM imidazole). Following buffer exchange to storage buffer (20 mM Tris (pH 7.5), 1 mM DTT, 200 mM NaCl, 10% glycerol) with spin columns, the protein was flash frozen and stored at -70 °C.

1.3.14 Confocal microscopy

Confocal imaging was performed on a Leica DMi8 microscope (Leica Microsystems) equipped with a Leica TCS SP8 X scan head, a SuperK white light laser, a 355 nm CW laser (Coherent), a HC PL APO 63x1.47 oil objective or a HC PL APO 40.0x1.10 water objective; SiR595-probes were excited at 590 nm, and SiR650 probes at 645 nm. Emissions for SiR595-probes were collected from 600 nm to 630 nm, for SiR650 from 670 nm to 750 nm. The microscope was equipped with a CO_2 and temperature controllable incubator (Life Imaging Services, 37 °C).

1.3.15 STED microscopy

STED microscopy images were taken on an Abberior easy3D STED/RESOLFT QUAD scanning microscope (Abberior Instruments GmbH, Göttingen, Germany) built on a motorized inverted microscope IX83 (Olympus, Tokyo, Japan). Fluorophores were excited with a pulsed laser diode at 561 nm or 640 nm wavelength (PicoQuant). The fluorescence detector is an avalanche photodiode (APD) and the microscope is equipped with a 775 nm pulsed STED laser. The SiR595 signal was acquired using a 590 nm to 630 nm detection window, and the SiR650 signal was acquired using a 660 nm to 750 nm detection window. A confocal channel with 485 nm excitation and 505 nm to 530 nm detection wavelengths was implemented to additionally image GFP. All images were acquired on 100x/1.40 UPlanSApo 100x/1.40 Oil 8/0.17/FN26.5 objectives. Pixel size, laser powers and dwell times were optimized for each sample. Note that all obtained images using STED microscopy were NOT deconvolved.

1.4 Synthesis Schemes

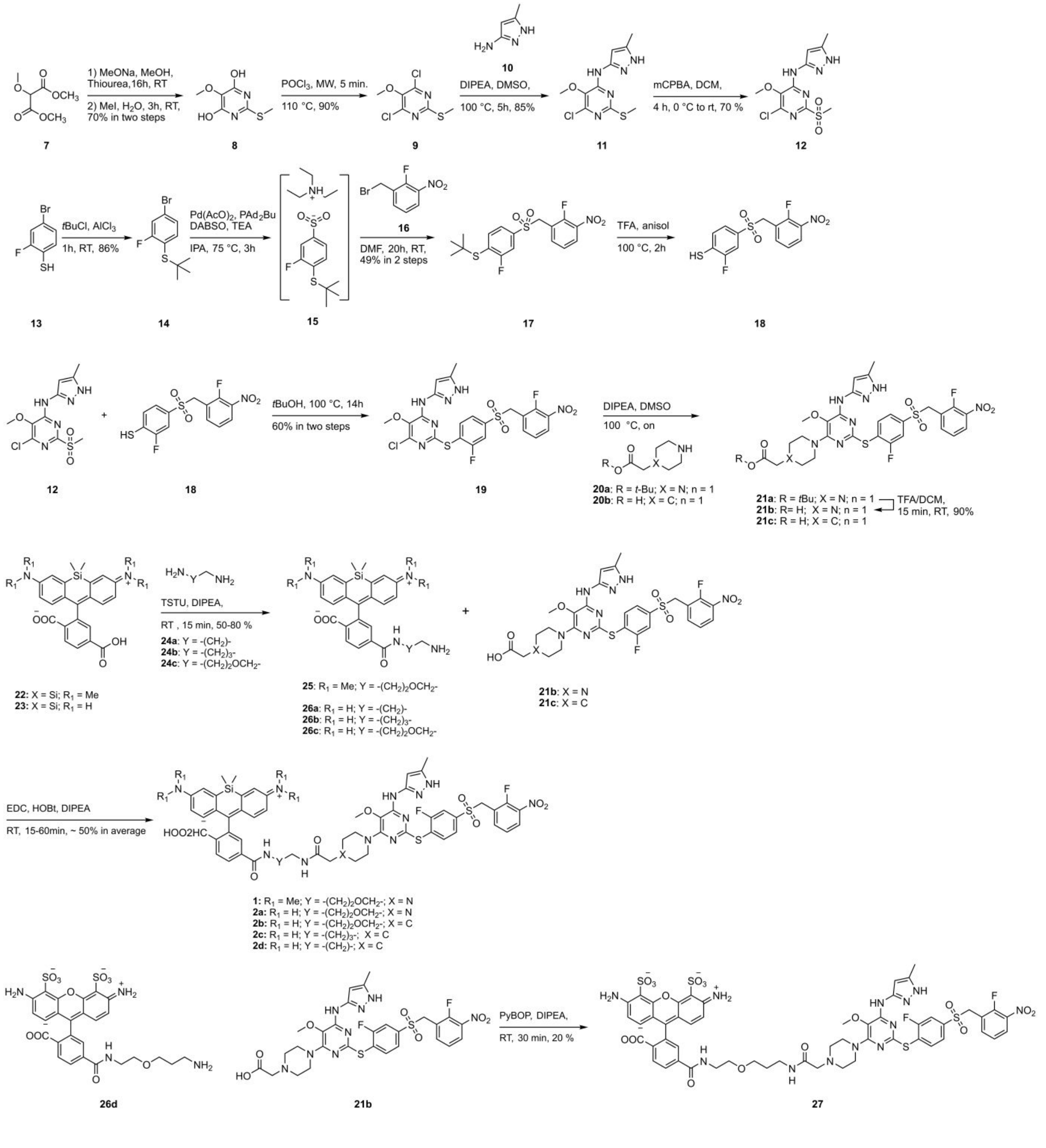

Supplementary Scheme 1. Synthesis of Fluorophore-Centrinone probes (**1, 2a-d, 27**)

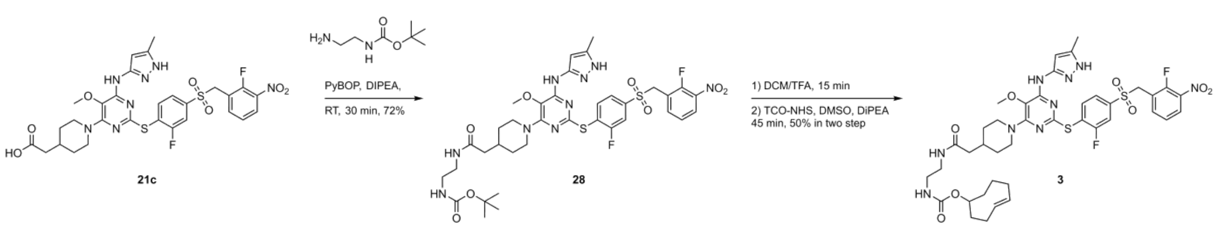

Supplementary Scheme 2. Synthesis of Cen-TCO (**3**) probe

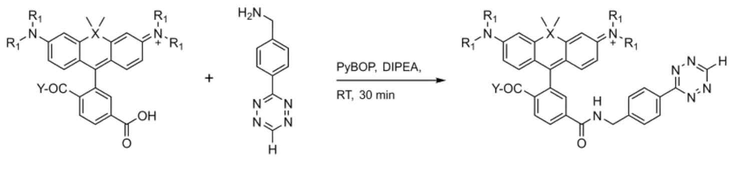

22: X = Si; R_1 = Me ; Y = OH
23: X = Si; R_1 = H; Y = OH
29: X = C; R_1 = Me; Y = SO_2Me

30

31a: X = Si; R_1 = Me ; Y = OH
31b: X = Si; R_1 = H; Y = OH
31c: X = C; R_1 = Me; Y = SO_2Me

Supplementary Scheme 3. Synthesis of fluorophore tetrazine probes (**31a-c**)

1.5 Synthesis protocols

1.5.1 4,6-Dihydroxy-5-methoxy thiopyrimidine **8**

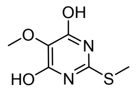

8

To a solution of dimethyl malonate **7** (200 mg, 2.23 mmol, 1.0 eq.) and thiourea (140 mg, 1.8 mmol, 1.5 eq.) in MeOH, a 20% sodium methoxide (170 mg, 3 mmol, 2.5 eq.) solution in methanol was added dropwise over 15 min at 0 °C, and the mixture was stirred at room temperature for 16 h. Upon reaction completion, MeOH was evaporated and the crude residue was dissolved in water (20 mL) before methyl iodide (270 mg, 120 µL,1.9 mmol, 1.5 eq.) was added, upon which the mixture was stirred at room temperature for 3 h. The reaction mixture was acidified with a HCl solution (6 M) to pH 3-4, and the product was allowed to precipitate. The precipitate was collected and the mother liquor was acidified 3 times to precipitate all of the product. The collected precipitate was washed with water and dried on air to afford the desired product **8** as a white powder (163 mg, 70 %).^{1}**H-NMR** (400 MHz, Methanol-d_4): δ = 3.72 (s, 3H), 2.53 (s, 3H) ppm.^{13}**C-NMR** (101 MHz, Methanol-d_4): δ =162.9, 157.4, 124.4, 60.3, 13.6. **HRMS (ESI):** calc. $C_6H_9N_2O_3S$ $[M+H]^+$: 189.0328; found 189.0333

1.5.2 4,6-Dichloro-5-methoxy thiopyrimidine **9**

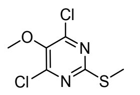

9

Compound **8** (500 mg, 2.66 mmol, 1.0 eq.) was added slowly into freshly distilled $POCl_3$ (4 mL) in a microwave vessel. The mixture was stirred at 110 °C for 5 min at 150 W in a microwave. Upon reaction, the reaction mixture was cooled to -20 °C and carefully quenched with ice. The product was extracted with EtOAc (3 x 20 mL) and the combined organic extracts were washed with brine, separated, dried over $MgSO_4$ and evaporated. The crude residue was dissolved in hexane and purified by flash column chromatography (hexane/DCM; gradient 0-50%) to obtain the desired product **9** as a beige powder (532 mg, 90%).^{1}**H-NMR** (400 MHz, Chloroform-*d*): δ = 3.91 (s, 3H), 2.55 (s, 3H) ppm.^{13}**C-NMR** (101 MHz, Chloroform-*d*): δ = 167.0, 155.3, 143.6, 61.4, 15.0 ppm**. HR-MS (EI):** calc. for $C_6H_7Cl_2N_2OS$ $[M+H]^+$: 224.9651, 226.9626, 228.9596 ; found 224.9656, 226.9631, 228,9599

1.5.3 6 - Chloro- 5 -methoxy- 4 - ((methyl-*1H*-pyrazol-3-yl) amino)-2- (methylthio) pyrimidine **11**

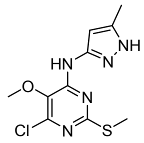

11

To a solution of compound **9** (40 mg, 118 µmol, 1.0 eq.) in DMSO, 5-methyl-*2H*-pyrazole-3-yl amine **10** (17 mg, 176 µmol, 1.5 eq.) and DIPEA (61 µL, 353 µmol, 3.0 eq.) were added. The reaction mixture was heated at 80 °C for 3 h before it was diluted with water (10 mL) and the product extracted with EtOAc (3 x 20 mL). The collected organic phase was washed with water and brine, separated, dried over $MgSO_4$ and evaporated. The crude residue was dissolved in EtOAc and purified by flash column chromatography (hexane/EtOAc; step gradient 0-100%) to obtain the desired product **11** as a brown powder (30 mg, 85%). **^{1}H NMR** (400 MHz, Methanol-d_4): δ = 6.45 (s, 1H), 3.86 (s, 3H), 2.51 (s, 3H), 2.29 (s, 3H) ppm. **^{13}C{^{1}H}-NMR** (101 MHz, Chloroform-*d*): δ = 167.3, 155.9, 150.1, 133.7, 98.0, 58.3, 18.4, 14.8 ppm. **HRMS (ESI):** calc. for $C_{10}H_{13}ClN_5OS$ $[M+H]^+$: 286.0529, 288.0500; found 286.0523, 288.0497

1.5.4 6-Chloro-5-methoxy-4-((5-methyl-1H-pyrazol-3-yl)amino)-2-(methylsulfonyl) pyrimidine **12**

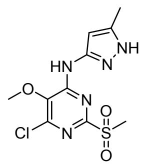

12

To a stirred solution of pyrimidine methyl sulfide **11** (250 mg, 0.87 mmol, 1 eq.) in DCM at 0 °C, a DCM solution of *m*CPBA (77% wt) (392 mg, 1.75 mmol, 2.0 eq.) was added. The reaction mixture was let warm up to r.t. and was stirred for 4 h. Upon completion, the mixture was diluted with DCM and washed with 50 % solution of $NaS_2O_3/NaHCO_3$ (3 x 50 mL). The organic phase was washed with water and brine, separated, dried over $MgSO_4$ and evaporated. The crude residue was dissolved and purified flash column chromatography (hexanes/EtOAc; step gradient 0-100%) to obtain the desired product **12** as white title powder (200 mg, 72 %). **^{1}H NMR** (400 MHz, DMSO-*d6*): δ = 10.38 (s, 1H), 6.47 (d, *J* = 0.8 Hz, 1H), 3.87 (s, 3H), 3.32 (s, 3H), 2.26 (s, 3H) ppm. **^{13}C{^{1}H}-NMR** (101 MHz, DMSO-*d6*): δ = 158.90, 155.52, 148.57, 146.36, 139.48, 137.70, 97.45, 61.24, 11.34 ppm. **HRMS (ESI)**: calc. for $C_{10}H_{12}ClN_5O_3S$ $[M+H]^+$: 318.0422; found 318.0420

1.5.5 1-(Bromomethyl)-2-fluoro-3-nitrobenzene **16**

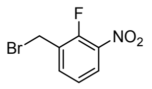

17

To a stirring solution of 1-methyl-2-fluro-3-nitrobenzene (2 g, 12.9 mmol, 1 eq.) and NBS (2.75 g, 15.5 mmol, 1.2 eq.), in 100 mL of DCE, was added AIBN (423 mg, 2.6 mmol, 0.2 eq.). The mixture was stirred under reflux for 6 h. The reaction mixture was cooled to r.t., diluted with DCM and washed with water, sat. $NaHCO_3$ and brine. To the organic phase, silica gel was added to obtain silica dry load upon solvent evaporation. Silica gel loaded with the compound was purified by flash chromatography (hexane/DCM; step gradient 0-2.5-5-10-50 %) to obtain the desired product **16** as yellow oil (1.36 g, 45 %).

^{1}H NMR (400 MHz, Chloroform-*d*) δ = 8.01 (ddd, *J* = 8.5, 7.0, 1.8 Hz, 1H), 7.71 (ddd, *J* = 7.9, 6.3, 1.8 Hz, 1H), 7.30 (td, *J* = 8.1, 1.3 Hz, 1H), 4.55 (d, *J* = 1.5 Hz, 2H) ppm. **^{13}C{^{1}H}-NMR** (101 MHz, Chloroform-*d*) δ = 153.60 (d, *J* = 267.8 Hz), 137.97, 136.64 (d, *J* = 3.7 Hz), 128.72 (d, *J* = 13.9 Hz), 126.35 (d, *J* = 2.6 Hz), 124.63 (d, *J* = 5.3 Hz), 23.74 (d, *J* = 5.5 Hz) ppm.

1.5.6 4-Bromo-2-fluoro-1-(*tert*-butylthio) benzene **14**

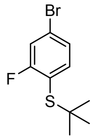

14

To the ice-cooled solution of thiophenol **13** (2 g, 9.7 mmol, 1 eq.) in 10 mL of *t*-BuCl, $AlCl_3$ (257 mg, 1.9 mmol, 0.2 eq.) was added in small portions. Evolved HCl was lead through solution of 2 M NaOH. Upon addition of $AlCl_3$, the ice bath was removed, and the reaction was stirred for 1h on r.t. Reaction mixture was poured into water and extracted with hexanes (3 x 20 mL). The pooled organic phases were washed with water, brine, dried over of an anh. $MgSO_4$ and filter. Prior to evaporation of organic phase, silica gel was added to form silica cake. Silica gel loaded with a compound was purified by flash chromatography (hexanes; isocratic) to obtain desired product **14** as a colourless oil (2.2 g, 86 %). **^{1}H NMR** (400 MHz, Chloroform-*d*) δ = 7.50 (dd, *J* = 8.2, 7.3 Hz, 1H), 7.29 (dd, *J* = 8.6, 2.0 Hz, 1H), 7.18 (ddd, *J* = 8.2, 1.9, 0.8 Hz, 1H), 1.30 (s, 9H) ppm. **^{13}C{^{1}H}-NMR** (101 MHz, Chloroform-*d*) δ = 164.04 (d, J = 250.9 Hz), 141.11, 127.58 (d, J = 4.1 Hz), 124.11 (d, J = 9.0 Hz), 119.74 (d, J = 27.9 Hz), 119.20 (d, J = 19.1 Hz), 47.65, 31.02 ppm.

1.5.7 *tert*-Butyl (2-fluoro-4-((2-fluoro-3-nitrobenzyl) sulfonyl) phenyl) sulfane **17**

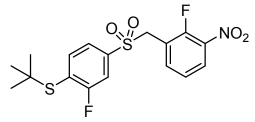

17

A glass tube was charged with DABSO (274 mg, 0.12 mmol, 0.6 eq.), palladium(II) acetate (43 mg, 190 µmol, 0.1 eq.) and CataCXium A® (136 mg, 380 µmol, 0.2 eq.), sealed with a rubber septum and evacuated and filled with nitrogen four times. Aryl bromide **14** (500 mg, 1.9 mmol, 1.0 eq.), anhydrous triethylamine (777 µL, 5.7 mmol, 3.0 eq.) and anhydrous 2-propanol (5 mL) were added sequentially through the septum and the mixture stirred under positive pressure of nitrogen in a preheated oil bath at 75 °C for 16 h Reaction was monitored by LC-MS, and when most of the starting material was transformed into sulfinic salt **15**, the solution was cooled to room temperature, and anh. DMF (~15 mL) solution of benzyl bromide **16** (533 mg, 2.3 mmol, 1.2 eq.) was added dropwise and the solution stirred at the same temperature for 6 h. Upon completion, the mixture was poured onto water (50 mL) and extracted with EtOAc (3 x 50 mL). The combined organic fractions were dried over $MgSO_4$, filtered and evaporated. Flash column chromatography (hexane/ EtOAc, 0–100 % gradient) afforded the titled sulfone **17** as a pale yellow solid (380 mg, 49 %). **^{1}H NMR** (400 MHz, Chloroform-*d*) δ = 8.06 (ddd, *J* = 8.6, 7.0, 1.8 Hz, 1H), 7.74 (ddd, *J* = 7.8, 6.0, 1.8 Hz, 1H), 7.68 (dd, *J* = 7.9, 6.6 Hz, 1H), 7.46 (dd, *J* = 7.3, 1.9 Hz, 1H), 7.43 (dd, *J* = 8.0, 2.0 Hz, 1H), 7.36 (td, *J* = 8.1, 1.3 Hz, 1H), 4.50 (d, *J* = 1.1 Hz, 2H), 1.32 (s, 9H) ppm. **^{13}C{^{1}H}-NMR** (101 MHz, Chloroform-*d*) δ = 163.70 (d, *J* = 252.8 Hz), 153.94 (d, *J* = 267.1 Hz), 139.67 (d, *J* = 6.4 Hz), 138.19 (d, *J* = 3.3 Hz), 137.78, 129.11 (d, *J* = 19.2 Hz), 127.29 (d, *J* = 2.4 Hz), 124.81 (d, *J* = 5.2 Hz), 123.72 (d, *J* = 4.4 Hz), 119.12 (d, *J* = 13.9 Hz), 116.06 (d, *J* = 28.1 Hz), 55.20 (d, *J* = 2.9 Hz), 49.05, 31.13 ppm. **^{19}F-NMR** (376 MHz, Chloroform-*d*): δ = -108.39 (d, *J* = 2.7 Hz), -122.09 (d, *J* = 2.7 Hz) ppm. **HRMS (ESI):** calc. for $C_{17}H_{17}F_2NO_4S_2$ $[M+Na]^+$: 424.0465; found 424.0459

1.5.8 6-chloro-2-((2-fluoro-4-((2-fluoro-3-nitrobenzyl)sulfonyl)phenyl)thio)-5-methoxy-4-((5-methyl-1H-pyrazol-3-yl)amino) pyrimidine **13**

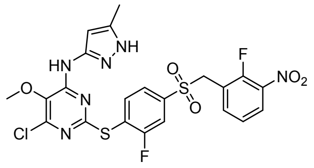

19

A reaction tube was charged with sulfone **17** (40 mg, 100 µmol, 1.3 eq.) and carefully dissolved in pure TFA followed by the addition of anisole (21.66 mg, 200 µmol, 2.6 eq.). The flask was sealed, air was exchanged with argon multiple times and the reaction mixture was heated to 100 °C for 2 h. Upon reaction completion, the mixture was cooled down to room temperature and TFA was evaporated. The crude containing compound **18** was kept under argon, before the dissolved methyl sulfone **12** (27 mg, 85 µmol, 1.0 eq.) in *tert*-butanol (10 mL) was added. The mixture was sealed and heated at 100 °C overnight under argon. Upon completion, the solvent was evaporated and crude was purified via RP-HPLC (H_2O\ACN = gradient 10–90 % (50 min.)) to afford the titled sulfone **19** as a pale yellow TFA salt (28 mg, 60 %). **^{1}H NMR** (400 MHz, DMSO-d_6) δ = 9.81 (s, 1H), 8.18 (ddd, *J* = 8.6, 7.1, 1.8 Hz, 1H), 8.00 (dd, *J* = 8.1, 6.8 Hz, 1H), 7.84 (dd, *J* = 8.1, 1.9 Hz, 1H), 7.67 (dd, *J* = 8.1, 1.9 Hz, 1H), 7.65 – 7.58 (m, 1H), 7.45 (t, *J* = 8.0 Hz, 1H), 5.45 (d, *J* = 0.8 Hz, 1H), 5.02 (s, 2H), 3.76 (s, 2H), 2.05 (s, 3H) ppm. **^{13}C{^{1}H}-NMR** (101 MHz, DMSO-d_6) δ 161.63 (d, *J* = 252.4 Hz), 161.16, 154.47, 153.47 (d, *J* = 265.9 Hz), 148.41, 145.78, 141.06 (d, *J* = 6.7 Hz), 139.03 (d, *J* = 3.8 Hz), 138.87, 138.04, 137.33 (d, *J* = 8.0 Hz), 133.33, 127.02 (d, *J* = 2.2 Hz), 124.94 (d, *J* = 4.8 Hz), 124.80 (d, *J* = 3.9 Hz), 124.22 (d, *J* = 18.4 Hz), 118.87 (d, *J* = 14.2 Hz), 115.97 (d, *J* = 26.4 Hz), 96.00, 60.69, 54.10, 10.80 ppm. **^{19}F NMR** (376 MHz, DMSO-d_6) δ = -102.18 (t, *J* = 7.4 Hz), -121.97 (t, *J* = 6.9 Hz) ppm. **HRMS (ESI):** calc. for $C_{22}H_{17}ClF_2N_6O_5S_2$ $[M+H]^+$: 583.0431, found 583.0436.

1.5.9 *tert*-Butyl-2-(4-(2-((2-fluoro-4-((2-fluoro-3-nitrobenzyl)sulfonyl)phenyl)thio)-5-methoxy-6-((5-methyl-1H-pyrazol-3-yl)amino)pyrimidin-4-yl)piperazin-1-yl)acetate **21a**

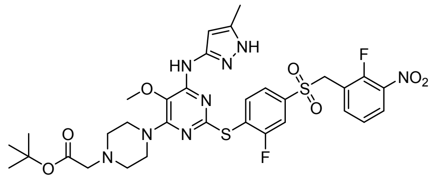

21a

Compound **19** (7 mg, 12 µmol, 1.0 eq.) was dissolved in anh. DMSO (1 mL) then piperazine derivative **20a** (2.9 mg, 14 µmol, 1.2 eq.) and DIPEA (2.3 mg, 18 µmol, 1.5 eq.) was added to the solution. The mixture was heated at 95 °C overnight. Upon cooling, the mixture was acidified with acetic acid (200 µL), followed by adding water (200 µL) before purification by RP-HPLC (ACN/H_2O; gradient 10-90%). Corresponding fractions were collected, frozen and lyophilized overnight to obtain the desired product **21a** as TFA salt in form of a white powder (5 mg, 56 %). **^{1}H NMR** (400 MHz, Chloroform-*d*) δ = 10.11 (s, 1H), 8.08 (ddd, *J* = 8.5, 7.0, 1.7 Hz, 1H), 7.84 (dd, *J* = 8.3, 6.4 Hz, 1H), 7.74 (ddd, *J* = 7.7, 5.9, 1.7 Hz, 1H), 7.56 (td, *J* = 7.7, 1.8 Hz, 2H), 7.44 – 7.32 (m, 1H), 6.01 (s, 1H), 4.54 (s, 2H), 4.02 (s, 4H), 3.79 (s, 2H), 3.66 (s, 3H), 3.36 (s, 4H), 2.66 (s, 2H), 2.25 (s, 3H), 1.46 (s, 10H) ppm.

1.5.10 2-(4-(2-((2-fluoro-4-((2-fluoro-3-nitrobenzyl)sulfonyl)phenyl)thio)-5-methoxy-6-((5-methyl-1H-pyrazol-3-yl)amino)pyrimidin-4-yl)piperazin-1-yl)acetic acid **21b**

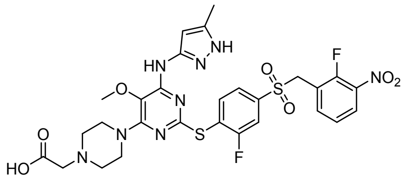

21b

Compound **21a** (5 mg, 6.7 µmol, 1 eq.) was dissolved in DCM containing 20 % of TFA. Reaction was stirred for 15 min. upon which DCM/TFA mixture was evaporated under vacuum. The residue was dissolved and purified by RP-HPLC (ACN/H_2O; gradient 10-90%). Corresponding fractions were collected, frozen and lyophilized overnight to obtain the desired product **21b** as TFA salt in form of a white powder (4.2 mg, 90 %). **^{1}H NMR** (400 MHz, DMSO-*d$_6$*) δ = 8.93 (s, 1H), 8.18 (ddd, *J* = 8.6, 7.1, 1.8 Hz, 1H), 7.96 (dd, *J* = 8.1, 6.8 Hz, 1H), 7.82 (dd, *J* = 8.1, 1.9 Hz, 1H), 7.65 (ddd, *J* = 8.1, 6.1, 1.8 Hz, 2H), 7.46 (t, *J* = 8.0 Hz, 1H), 5.48 (s, 1H), 5.01 (s, 2H), 4.15 (zs, 2H), 3.96 (broad peak, 4H), 3.57 (s, 3H), 3.34 (broad peak, 4H), 2.04 (s, 3H) ppm. **^{13}C{^{1}H}-NMR** (101 MHz, DMSO-*d$_6$*) δ = 167.74, 163.31, 160.59 (d, *J* = 44.4 Hz), 154.76 (d, *J* = 87.5 Hz), 153.01 (d, *J* = 90.6 Hz), 146.89, 141.18 (d, *J* = 6.9 Hz), 139.02 (d, *J* = 105.7 Hz), 137.76 (d, *J* = 8.1 Hz), 127.47, 125.65, 125.43 (d, *J* = 5.6 Hz), 125.06, 123.32, 119.28, 118.18, 116.21 (d, *J* = 26.5 Hz), 115.25, 95.77, 59.24, 55.74, 54.57, 51.87, 42.90, 11.40 ppm. **^{19}F NMR** (376 MHz, DMSO-*d$_6$*) δ = -102.27 (t, *J* = 7.4 Hz), -122.12 (d, *J* = 6.7 Hz) ppm.

1.5.11 2-(1-(2-((2-fluoro-4-((2-fluoro-3-nitrobenzyl)sulfonyl)phenyl)thio)-5-methoxy-6-((5-methyl-1H-pyrazol-3-yl)amino)pyrimidin-4-yl)piperidin-4-yl) acetic acid **21c**

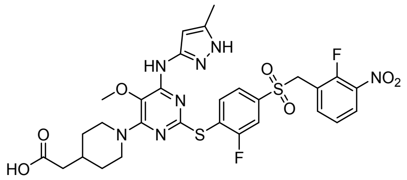

21c

Compound **19** (10 mg, 17 µmol, 1.0 eq.) was dissolved in anh. DMSO (1 mL) then piperidine derivative **20b** (3.7 mg, 26 µmol, 1.5 eq.) and DIPEA (11 mg, 86 µmol, 5 eq.) was added to the solution. The mixture was heated at 95°C overnight. Upon cooling, the mixture was acidified with acetic acid (200 µL), followed by addition of water (200 µL) before purification by RP-HPLC (ACN/H_2O; gradient 10-90%). The corresponding fractions were collected, frozen and lyophilized overnight to obtain the desired product **21c** as TFA salt in form of white powder (5 mg, 42 %).

^{1}H NMR (400 MHz, DMSO-d_6) δ = 8.94 (s, 1H), 8.17 (ddd, *J* = 8.6, 7.1, 1.8 Hz, 1H), 7.96 (dd, *J* = 8.1, 6.8 Hz, 1H), 7.81 (dd, *J* = 8.1, 1.9 Hz, 1H), 7.68 – 7.61 (m, 2H), 7.45 (t, *J* = 8.0 Hz, 1H), 5.64 (s, 1H), 5.01 (s, 2H), 4.18 (d, *J* = 13.0 Hz, 3H), 3.54 (s, 3H), 2.81 (td, *J* = 12.9, 2.4 Hz, 2H), 2.15 (d, *J* = 6.9 Hz, 2H), 2.09 (s, 3H), 1.88 (ddd, *J* = 11.1, 7.2, 3.8 Hz, 0H), 1.69 – 1.61 (m, 2H), 1.14 (qd, *J* = 12.5, 3.8 Hz, 2H) ppm. **^{13}C{^{1}H}-NMR** (101 MHz, DMSO-d_6) δ = 173.40, 162.87, 160.37, 159.56, 154.22 (d, *J* = 111.2 Hz), 152.68 (d, *J* = 111.1 Hz), 145.90, 140.64 (d, *J* = 6.7 Hz), 140.41, 139.06, 138.03, 137.32 (d, *J* = 8.0 Hz), 127.01, 125.46 (d, *J* = 18.5 Hz), 124.95 (d, *J* = 4.8 Hz), 124.47, 122.15, 118.91 (d, *J* = 14.4 Hz), 115.61 (d, *J* = 26.5 Hz), 95.14, 58.66, 54.04, 45.61, 40.50, 32.58, 31.48, 11.04 ppm. **^{19}F NMR** (376 MHz, DMSO-d_6) δ = -102.31(t, *J* = 7.4 Hz, -122.02(t, *J* = 6.9 Hz) ppm. **HRMS (ESI):** calc. for $C_{29}H_{29}F_2N_7O_7S_2$ $[M+H]^+$: 690.1611; found 690.1614.

1.5.12 SiR595’6-COOH **22**, SiR650’6-COOH **23,** CPY-SO2Me- COOH **28**

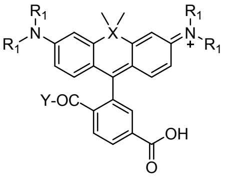

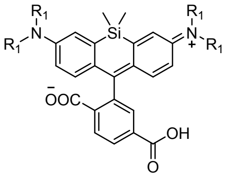

22: X = Si; R_1 = Me ; Y = OH
23: X = Si; R_1 = H; Y = OH
28: X = C; R_1 = Me; Y = SO_2Me

22: X = Si; R_1 = Me
22: X = Si; R_1 = H

These compounds were synthesized according to published literature.[4-5]

1.5.13 SiR650 – PEG linker **25**

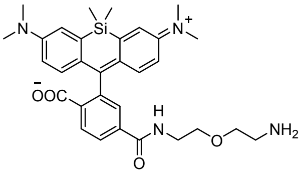

25

The compound was synthesized according to published literature.[6]

1.5.14 Alexa488 – PEG linker **26d**

The compound was synthesized according to published literature.

1.5.15 SiR595 - linker **26a-c**

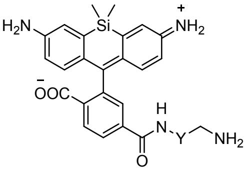

26a: Y = -(CH_2)-
26b: Y = -(CH_2)$_3$-
26c: Y = -(CH_2)$_2$OCH_2-

General procedure: A 2 mL vial was charged with SiR595-6’-COOH **22** (3 mg, 7.2 µmol, 1.0 eq) in DMSO (1 mL) and DIPEA (4.65 mg, 36 µmol, 5.0 eq.) and TSTU (2.4 mg, 7.9 µmol, 1.1) were added in one portion. Upon addition, the mixture turned from blue to colourless (DIPEA) to yellow (TSTU). After the mixture was stirred at rt for 5 min, diamine **24a-c** (10 eq) was added and the reaction mixture was vigorously stirred for 15 min and then quenched with HOAc (25 eq) and 0.1% aqueous TFA (~100 µL per 3 mg of SiR595-6’-COOH). The crude was subjected to RP-HPLC (ACN/H_2O; gradient 10-90%) to obtain **26a** (51%, 7.2 mM, 510 µL), **26b** (44%, 5.7 mM, 500 µL) and **26c** (69%, 10 mM, 500 µL) as blue powder that was dissolved in dry DMSO. Concentration was measured by UV-Vis spectrometry.

20a:

^{1}H NMR (400 MHz, DMSO-*d*$_6$) δ = 8.85 (t, *J* = 5.6 Hz, 1H), 8.15 – 8.06 (m, 2H), 7.78 (s, 2H), 7.73 (s, 1H), 7.10 (d, *J* = 2.2 Hz, 2H), 6.64 (d, *J* = 3.1 Hz, 4H), 5.58 (broad peak, 4H), 3.48 (q, *J* = 6.0 Hz, 2H),

2.96 (q, *J* = 5.9 Hz, 2H), 0.57 (s, 3H), 0.48 (s, 3H) ppm.**HRMS (ESI):** calc. for $C_{25}H_{26}N_4O_3Si$ $[M+H]^+$: 459.1847; found 459.1847

20b:

^{1}H NMR (400 MHz, DMSO-*d*$_6$) δ = 8.81 (t, *J* = 5.7 Hz, 1H), 8.13 – 8.00 (m, 2H), 7.71 (s, 1H), 7.68 (s, 2H), 7.12 (d, *J* = 2.3 Hz, 2H), 6.72 – 6.59 (m, 4H), 5.58 (broad peak, 4H) 3.25 (q, *J* = 5.7 Hz, 2H), 2.78 (q, *J* = 6.1 Hz, 2H), 1.59 – 1.46 (m, 4H), 0.58 (s, 3H), 0.48 (s, 3H) ppm.**^{13}C{^{1}H}-NMR** (101 MHz, DMSO-*d*$_6$) δ = 169.00, 165.05, 154.13, 145.87, 139.61, 136.11, 132.13, 128.13, 127.8, 125.79, 123.09, 122.8, 120.09, 116.81, 38.73, 38.63, -0.13, -1.46. **HRMS (ESI):** calc. for $C_{27}H_{30}N_4O_3Si$ $[M+H]^+$: 486.2087; found 486.2086

20c:

^{1}H NMR (400 MHz, DMSO-*d*$_6$) δ = 8.79 (t, *J* = 5.6 Hz, 1H), 8.14 – 8.04 (m, 2H), 7.82 (s, 2H), 7.74 – 7.71 (m, 1H), 7.15 (d, *J* = 2.9 Hz, 2H), 6.72 – 6.63 (m, 5H), 3.61 – 3.57 (m, 2H), 3.55 (d, *J* = 5.7 Hz, 2H), 3.46 (t, *J* = 5.6 Hz, 2H), 2.98 (q, *J* = 5.5 Hz, 2H), 0.59 (s, 3H), 0.50 (s, 3H) ppm.**^{13}C{^{1}H}-NMR** (101 MHz, DMSO-*d*$_6$) δ = 169.01, 164.74, 154.13, 146.55, 139.84f, 136.04, 131.60, 128.09, 127.97, 127.70,127.59, 125.33, 123.18, 122.69, 119.63, 117.53, 116.40, 114.60, 38.72, 38.64, 25.99, 24.58, -0.10, -1.f45. **HRMS (ESI):** calc. for $C_{27}H_{30}N_4O_4Si$ $[M+H]^+$: 503.2109; found 503.2107

1.5.16 SiR650/595-PEG-N-Centrinone probes **1 & 2a**

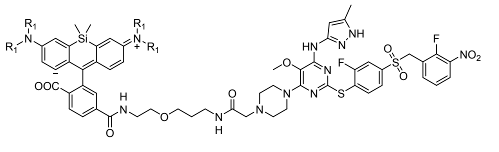

21a: R_1 = Me;
22a: R_1 = H

Compound **15b** (1 mg, 1.5 µmol, 1 eq.) was dissolved in DMSO dissolved in 1.5 mL Eppendorf vial to which DIPEA (2 mg (3 uL), 14.5 µmol, 10 eq.), EDC (0.25 mg, 1.6 µmol, 1.1 eq.), HOBt (0.25 mg, 0.3 µmol, 0.20 eq.) and 1.2 eq. of were added **19**, **20c, 27** or were added. Mixture was incubated for 6 h at room temperature. Reaction was followed by LC-MS and upon reaction completion, HOAc (~100 µL) and H2O (~100 µL) were added. The crude was subjected to RP-HPLC (ACN/H_2O; gradient 10-90%) to obtain **21** (23 %, 3 mM, 100 µL) or **22a** (29 %, 3.3 mM, 100 µL) or **27** (21 %, 3.1 mM, 100 uL) as powder that was dissolved in DMSO.

1.5.17 SiR595-linker-C-Centrinone probes **2b-d**

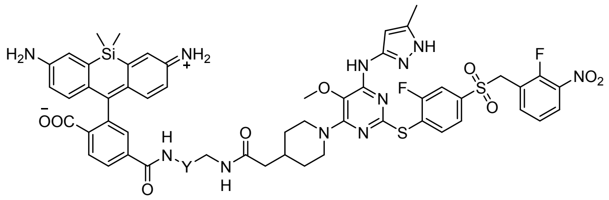

22b: Y = -$(CH_2)_2OCH_2$-
22c: Y = -$(CH_2)_3$-
22d: Y = -(CH_2)-

General procedure: Compound **21c** (2 mg, 2.9 µmol, 1 eq.) was dissolved in DMSO dissolved in 1.5 mL Eppendorf vial to which DIPEA (4 mg (3 uL), 29 µmol, 10 eq.), PyBOP (1.54 mg, 3.48 µmol, 1.2 eq.) and **26c** (2.38 mg, 4.9 µmol, .1.7 eq.). Mixture was incubated for 45 min at room temperature. Reaction was followed by LC-MS and upon reaction completion, HOAc (~100 µL) and H2O (~100 µL) were

added. The crude was subjected to RP-HPLC (ACN/H_2O; gradient 10-90%) to obtain **2b** (88%, 10 mM, 255 µL) as blue powder that was dissolved in DMSO.

20b:

^{1}H NMR (400 MHz, DMSO-*d*$_6$) δ = 8.94 (s, 1H), 8.82 (t, J = 5.5 Hz, 1H), 8.17 (ddd, J = 8.6, 7.1, 1.8 Hz, 1H), 8.12 (dd, J = 8.1, 1.4 Hz, 1H), 8.05 (d, J = 8.0 Hz, 1H), 7.95 (dd, J = 8.1, 6.8 Hz, 1H), 7.85 (t, J = 5.7 Hz, 1H), 7.81 (dd, J = 8.0, 2.0 Hz, 1H), 7.76 – 7.73 (m, 1H), 7.68 – 7.59 (m, 2H), 7.45 (t, J = 8.0 Hz, 1H), 7.16 (d, J = 2.2 Hz, 2H), 6.74 – 6.64 (m, 4H), 5.61 (s, 1H), 5.01 (s, 2H), 4.14 (d, J = 12.7 Hz, 2H), 3.87 (broad peak, 4H), 3.51 (s, 3H), 3.49 (d, J = 5.7 Hz, 2H), 3.39 (t, J = 5.7 Hz, 4H), 3.17 (q, J = 5.8 Hz, 2H), 2.77 (d, J = 12.6 Hz, 2H), 2.08 (s, 3H), 1.95 (d, J = 7.0 Hz, 2H), 1.88 – 1.79 (m, 1H), 1.55 (d, J = 12.1 Hz, 2H), 1.11 – 1.00 (m, 2H), 0.58 (s, 3H), 0.49 (s, 3H). **^{13}C{^{1}H}-NMR** (101 MHz, DMSO-*d*$_6$) δ = 170.77, 164.64, 163.10, 162.75 ,160.39, 159.65, 154.77, 153.5 (d, J = 35.71 Hz), 152.08, 146.10, 140.64, 139.93,138.9, 138.11, 137.40, 133.22, 128.32, 127.59, 127.1 (d, 24.1 Hz), 125.35, 124.98, 124.53, 122.11, 118.85 (d, 13.4 Hz), 117.31, 115.57, 114.37, 95.16, 58.58, 48.66, 45.74, 42.42, 38.94, 37.87, 33.15, 31.66, 26.81, 26.50, 11.07, -0.08, -1.56. **^{19}F NMR** (376 MHz, DMSO-*d*$_6$) δ = -102.19 (t, J = 7.5 Hz), -122.00 (t, J = 7.0 Hz). **HRMS (ESI):** calc. for $C_{56}H_{57}F_2N_{11}O_{10}S_2Si$ [M+2H]$^+$: 587.6807; found 587.6804.

20c:

^{1}H NMR (400 MHz, DMSO-*d*$_6$) δ = 8.87 (s, 1H), 8.78 (t, J = 5.7 Hz, 1H), 8.17 (t, J = 7.6 Hz, 1H), 8.14 – 8.03 (m, 2H), 7.96 (t, J = 7.5 Hz, 1H), 7.81 (d, J = 8.4 Hz, 2H), 7.73 (s, 1H), 7.64 (d, J = 7.7 Hz, 2H), 7.45 (t, J = 8.0 Hz, 1H), 7.13 (s, 2H), 6.66 (t, J = 6.3 Hz, 4H), 5.59 (s, 1H), 5.02 (s, 2H), 4.14 (s, 2H), 3.81 (broad peak, 4H), 3.52 (s, 3H), 3.23 (d, J = 6.3 Hz, 2H), 3.03 (q, J = 6.3 Hz, 2H), 2.81 – 2.74 (m, 2H), 2.08 (s, 3H), 1.96 (d, J = 7.2 Hz, 2H), 1.87 (s, 1H), 1.58 (d, J = 12.2 Hz, 2H), 1.43 (dq, J = 29.0, 7.7 Hz, 4H), 1.08 (m, 2H), 0.58 (s, 3H), 0.49 (s, 3H). **^{13}C{^{1}H}-NMR** (101 MHz, DMSO-*d*$_6$) δ = 170.77, 164.64, 163.10, 162.75 ,160.39, 159.65, 154.77, 153.5 (d, J = 35.71 Hz), 152.08, 146.10, 140.64, 139.93,138.9, 138.11, 137.40, 133.22, 128.32, 127.59, 127.1 (d, 24.1 Hz), 125.35, 124.98, 124.53, 122.11, 118.85 (d, 13.4 Hz), 117.31, 115.57, 114.37, 95.16, 58.58, 48.66, 45.74, 42.42, 38.94, 37.87, 33.15, 31.66, 26.81, 26.50, 11.07, -0.08, -1.56. **^{19}F NMR** (376 MHz, DMSO-*d*$_6$) δ = -102.21 (t, *J* = 7.3 Hz), -121.99 (t, *J* = 6.8 Hz). **HRMS (ESI):** calc. $C_{56}H_{57}F_2N_{11}O_9S_2Si$ [M+2H]$^+$: 579.6833; found 579.6827.

20d:

^{1}H NMR (400 MHz, DMSO-*d*$_6$) δ = 8.79 (m, 2H), 8.21 – 8.12 (m, 1H), 8.12 – 8.02 (m, 2H), 7.99 – 7.90 (m, 2H), 7.81 (dd, J = 8.0, 1.9 Hz, 1H), 7.71 (s, 1H), 7.64 (ddd, J = 7.8, 5.2, 1.8 Hz, 2H), 7.44 (t, J = 8.0 Hz, 1H), 7.10 (s, 2H), 6.64 (d, J = 2.1 Hz, 4H), 5.56 (s, 1H), 5.01 (s, 2H), 4.12 (d, J = 12.6 Hz, 3H), 3.53 (broad peak, 4H), 3.49 (s, 3H), 3.27 (s, 2H), 3.19 (d, J = 6.2 Hz, 2H), 2.72 (t, J = 12.2 Hz, 2H), 2.07 (s, 3H), 1.95 (d, J = 7.2 Hz, 2H), 1.84 (s, 1H), 1.55 (d, J = 12.2 Hz, 2H), 1.05 (q, J = 11.4, 10.5 Hz, 2H), 0.57 (s, 3H), 0.48 (s, 3H). **^{13}C{^{1}H}-NMR** (101 MHz, DMSO-*d*$_6$) δ = 170.77, 164.64, 163.10, 162.75 ,160.39, 159.65, 154.77, 153.5 (d, J = 35.71 Hz), 152.08, 146.10, 140.64, 139.93,138.9, 138.11, 137.40, 133.22, 128.32, 127.59, 127.1 (d, 24.1 Hz), 125.35, 124.98, 124.53, 122.11, 118.85 (d, 13.4 Hz), 117.31, 115.57, 114.37, 95.16, 58.58, 48.66, 45.74, 42.42, 38.94, 37.87, 33.15, 31.66, 26.81, 26.50, 11.07, -0.08, -1.56. **^{19}F NMR** (376 MHz, DMSO-*d*$_6$) δ = -102.20 (t, *J* = 7.4 Hz), -122.00 (t, *J* = 6.7 Hz). **HRMS (ESI):** calc. $C_{54}H_{53}F_2N_{11}O_9S_2Si$ [M+H]$^+$: 1129.3206; found 1129.3205.

1.5.18 *tert*-butyl (2-(2-(1-(2-((2-fluoro-4-((2-fluoro-3-nitrobenzyl) sulfonyl) phenyl) thio) - 5-methoxy-6-((5-methyl-1H-pyrazol-3-yl) amino) pyrimidin-4-yl) piperidin- 4-yl) acetamido) ethyl) carbamate **27**

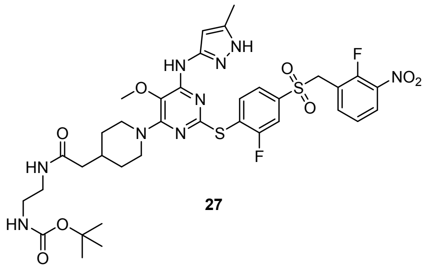

27

Compound **21c** (4 mg, 5.8 µmol, 1 eq.) was dissolved in DMSO dissolved in 1.5 mL Eppendorf vial to which DIPEA (4 mg (3 uL), 29 µmol, 5 eq.), PyBOP (2.82 mg, 6.38 µmol, 1.1 eq.) and N-Boc mono protected ethylene diamine (1.2 mg, 7.54 µmol, 1.3 eq.). Mixture was incubated for 30 min at room temperature. Reaction was followed by LC-MS and upon reaction completion, HOAc (~100 µL) and H2O (~100 µL) were added. The crude was subjected to RP-HPLC (ACN/H_2O; gradient 10-90%). The corresponding fractions were collected, frozen and lyophilized overnight to obtain the desired product to obtain TFA salt of **27** (72%, 3.5 mg) as white powder.

^{1}H NMR (400 MHz, DMSO-d_6) δ = 95 (s, 1H), 8.17 (ddd, *J* = 8.6, 7.1, 1.8 Hz, 1H), 7.96 (dd, *J* = 8.1, 6.8 Hz, 1H), 7.85 – 7.77 (m, 2H), 7.69 – 7.58 (m, 2H), 7.45 (t, *J* = 8.0 Hz, 1H), 6.75 (t, *J* = 5.6 Hz, 1H), 5.63 (s, 1H), 5.01 (s, 2H), 4.16 (d, *J* = 12.9 Hz, 2H), 3.53 (s, 3H), 3.09 – 3.01 (m, 2H), 2.95 (q, *J* = 6.2 Hz, 2H), 2.80 (t, *J* = 12.5 Hz, 2H), 2.09 (s, 3H), 1.98 (d, *J* = 7.1 Hz, 2H), 1.90 (m, 1H), 1.60 (d, *J* = 12.6 Hz, 2H), 1.37 (s, 9H), 1.09 (q, *J* = 12.2, 11.6 Hz, 2H) ppm. **^{13}C{^{1}H}-NMR** (101 MHz, DMSO-d_6) δ = 171.02, 162.83, 160.37, 159.56, 158.47, 158.10, 155.58, 154.63*, 153.70, 153.13, 152.10, 145.75, 140.60, 139.01, 138.00, 137.34, 126.99, 125.4 (d, J = 18.7 Hz), 124.91, 124.52, 122.17, 118.87, 116.94, 115.47, 95.10, 77.61, 58.61, 54.01, 45.67, 42.36, 39.71*, 38.56, 33.02, 31.56, 28.22, 11.00 ppm. **^{19}F NMR** (376 MHz, DMSO-d_6) δ = -102.27 (t, *J* = 7.5 Hz), -122.03 ppm.

1.5.19 (E)-cyclooct-4-en-1-yl (2-(2-(1-(2-((2-fluoro-4-((2-fluoro-3-nitrobenzyl) sulfonyl) phenyl) thio) -5-methoxy-6-((5-methyl-1H-pyrazol-3-yl)amino) pyrimidin-4-yl)piperidin-4-yl) acetamido)ethyl)carbamate **25**

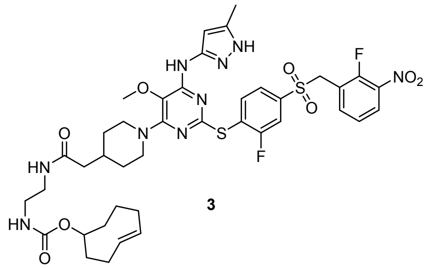

3

Compound **27** (3 mg, 3.6 µmol, 1 eq.) was dissolved in DCM containing 20 % of TFA. Reaction was stirred for 15 min. upon which DCM/TFA mixture was evaporated under vacuum. The mixture was dissolved into DMSO and DIPEA (2.3 mg, 17.8 µmol, 5 eq.) and TCO-NHS (1.1 mg, 3.9 µmol, 1.1 eq.) were added. Reaction mixture was stirred for 45 min. at room temperature. Reaction was followed by LC-MS and upon reaction completion, HOAc (~100 µL) and H2O (~100 µL) were added. The crude was subjected to RP-HPLC (ACN/H_2O; gradient 10-90%). The corresponding fractions were collected, frozen and lyophilized overnight to obtain the desired product to obtain TFA salt of **3** (50% in two steps, 1.6 mg) as white powder.

^{1}H NMR (400 MHz, DMSO-d_6) δ = 8.99 (s, 1H), 8.18 (ddd, J = 8.6, 7.1, 1.7 Hz, 1H), 7.97 (dd, J = 8.1, 6.8 Hz, 1H), 7.85 – 7.76 (m, 2H), 7.71 – 7.61 (m, 2H), 7.46 (t, J = 8.0 Hz, 1H), 6.91 (t, J = 5.7 Hz, 1H), 5.64 (s, 1H), 5.57 (td, J = 11.0, 5.2 Hz, 1H), 5.47 – 5.39 (m, 1H), 5.02 (s, 4H), 4.17 (d, J = 13.9 Hz, 3H), 3.54 (s, 2H), 3.05 (t, J = 5.9 Hz, 2H), 2.98 (d, J = 6.8 Hz, 2H), 2.80 (t, J = 12.4 Hz, 2H), 2.27 (d, J = 5.1 Hz, 2H), 2.10 (s, 3H), 1.97 (d, J = 7.0 Hz, 2H), 1.89 (m, 5H), 1.60 (m, 4H), 1.32 – 1.18 (m, 2H), 1.10 (dd, J = 18.1, 6.5 Hz, 2H) ppm. **^{13}C{^{1}H}-NMR** (101 MHz, DMSO-d_6) δ = 171.02, 162.85, 160.35, 159.55, 155.78, 154.41 (d, 57.22 Hz), 153.4 (d, 265.93 Hz), 145.77, 140.61, 139.04, 138.00, 137.34, 134.89, 132.50, 126.99, 125.4(d, 18.45 Hz), 124.89, 124.58, 122.16, 118.88 (d, 14.25), 116.89, 115.67, 114.00, 95.10, 79.08, 58.60, 54.00, 53.57, 45.67, 42.35, 40.66, 38.51, 38.17, 33.73, 33.01, 32.15, 31.56, 30.59, 18.07, 16.71, 11.00 ppm. **^{19}F NMR (376 MHz, DMSO-d_6) δ =** -102.26 (t, J = 7.5 Hz), -122.03 (d, J = 6.8 Hz). **HRMS (ESI):** calc. for $C_{36}H_{43}F_2N_9O_8S_2$ $[M+H]^+$: 831.2644; found 831. 2640

1.5.20 Synthesis of tetrazine fluorophore conjugates

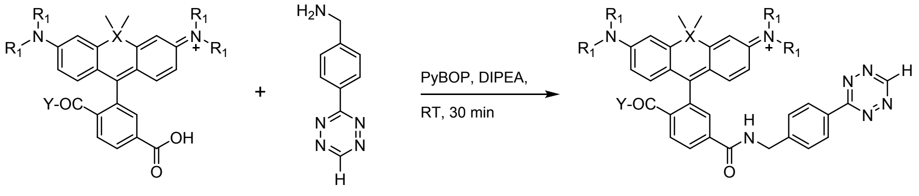

22: X = Si; R_1 = Me ; Y = OH
23: X = Si; R_1 = H; Y = OH
28: X = C; R_1 = Me; Y = SO_2Me

29

30a: X = Si; R_1 = Me ; Y = OH
30b: X = Si; R_1 = H; Y = OH
30c: X = C; R_1 = Me; Y = SO_2Me

General procedure: A fluorescent dye **22** (3 mg, 5.8 µmol, 1 eq.) was dissolved in DMSO (0.5 mL) followed by the addition of the DIPEA (4.65 mg, 36.0 µmol, 5 eq.), PyBOP (3.5 mg, 7.9 µmol, 1.1 eq.) and **29** (1.5 mg, 7.9 µmol, 1.1 eq.). Mixture was incubated for 30 min. at room temperature. Reaction was followed by LC-MS and upon reaction completion, HOAc (~100 µL) and H2O (~100 µL) were added. The crude was subjected to RP-HPLC (ACN/H_2O; gradient 10-90%) to obtain **30a** (2.35 mg, 55 %)

30b:

^{1}H NMR (400 MHz, DMSO-d_6) δ = 9.45 (s, 1H), 8.42 (d, J = 8.0 Hz, 2H), 8.19 (d, J = 8.1 Hz, 1H), 8.08 (d, J = 8.1 Hz, 1H), 7.79 (s, 1H), 7.58 (d, J = 8.1 Hz, 2H), 7.08 (s, 2H), 6.64 (s, 4H), 4.58 (d, J = 5.8 Hz, 2H),3.67 (broad peak, 4H), 2.98 (s, 3H), 0.57 (s, 3H), 0.48 (s, 3H) ppm. **^{13}C{^{1}H}-NMR** (101 MHz, DMSO-d_6) δ = 167.06, 164.95, 163.14, 158.29, 157.93, 143.83, 139.55*, 130.49, 128.36, 128.24, 127.96*, 127.48, 117.31, 116.99, 42.74, f20.81, -0.12, -1.57 ppm.

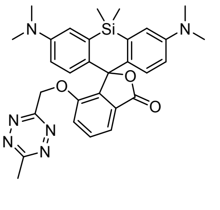

29

HD653 (**5**) was provided by Wombacher lab.[7]

1.6 NMR characterization

1.6.1 4,6-Dihydroxy-5-methoxy thiopyrimidine **8**

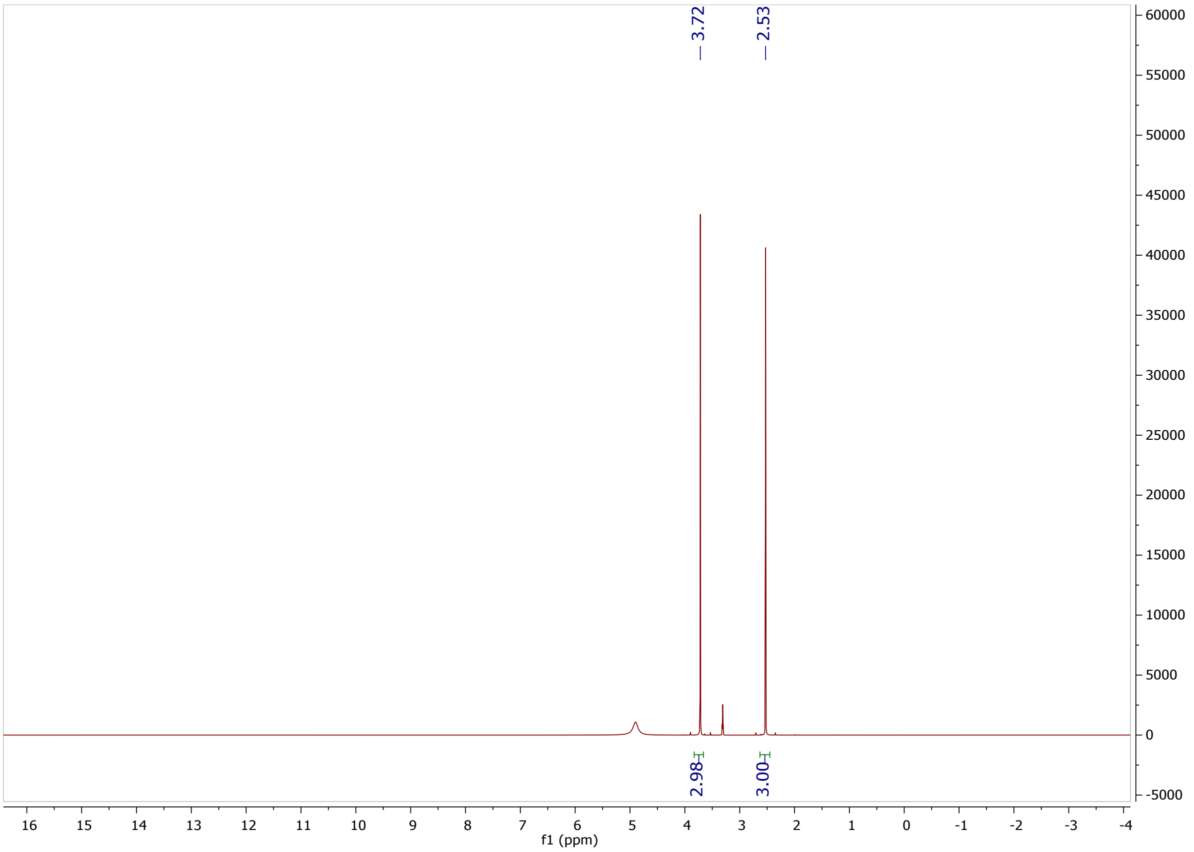

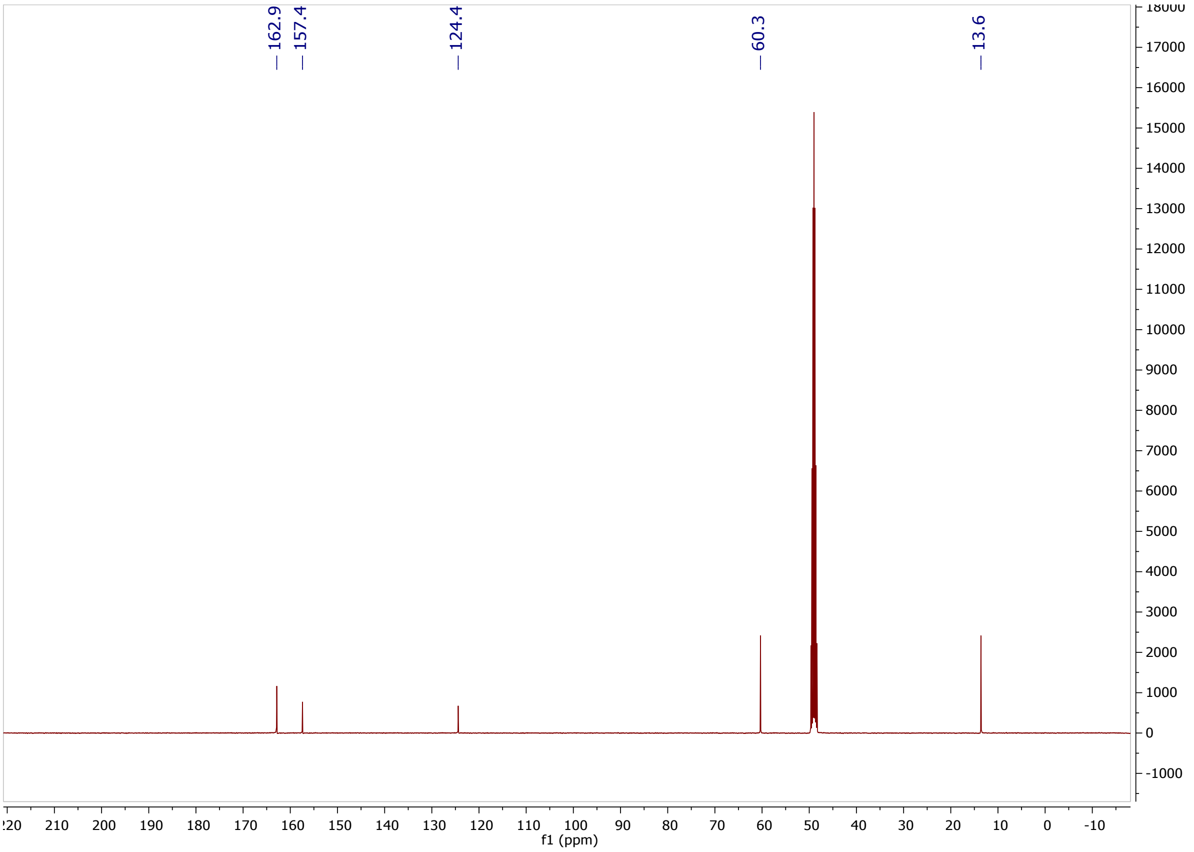

1.6.2 6-Chloro-5-methoxy-4-((methyl-*1H*-pyrazol-3-yl)amino)-2-(methylthio) pyrimidine **9**

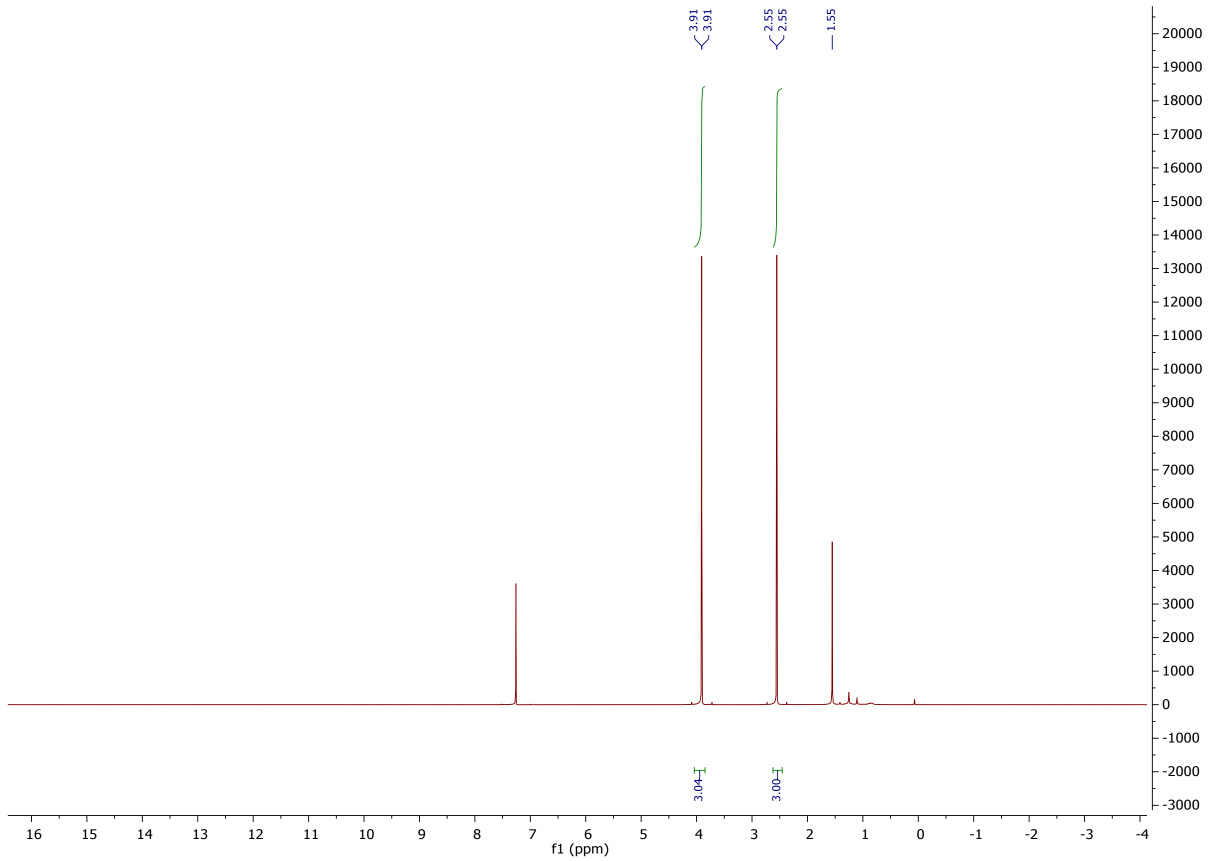

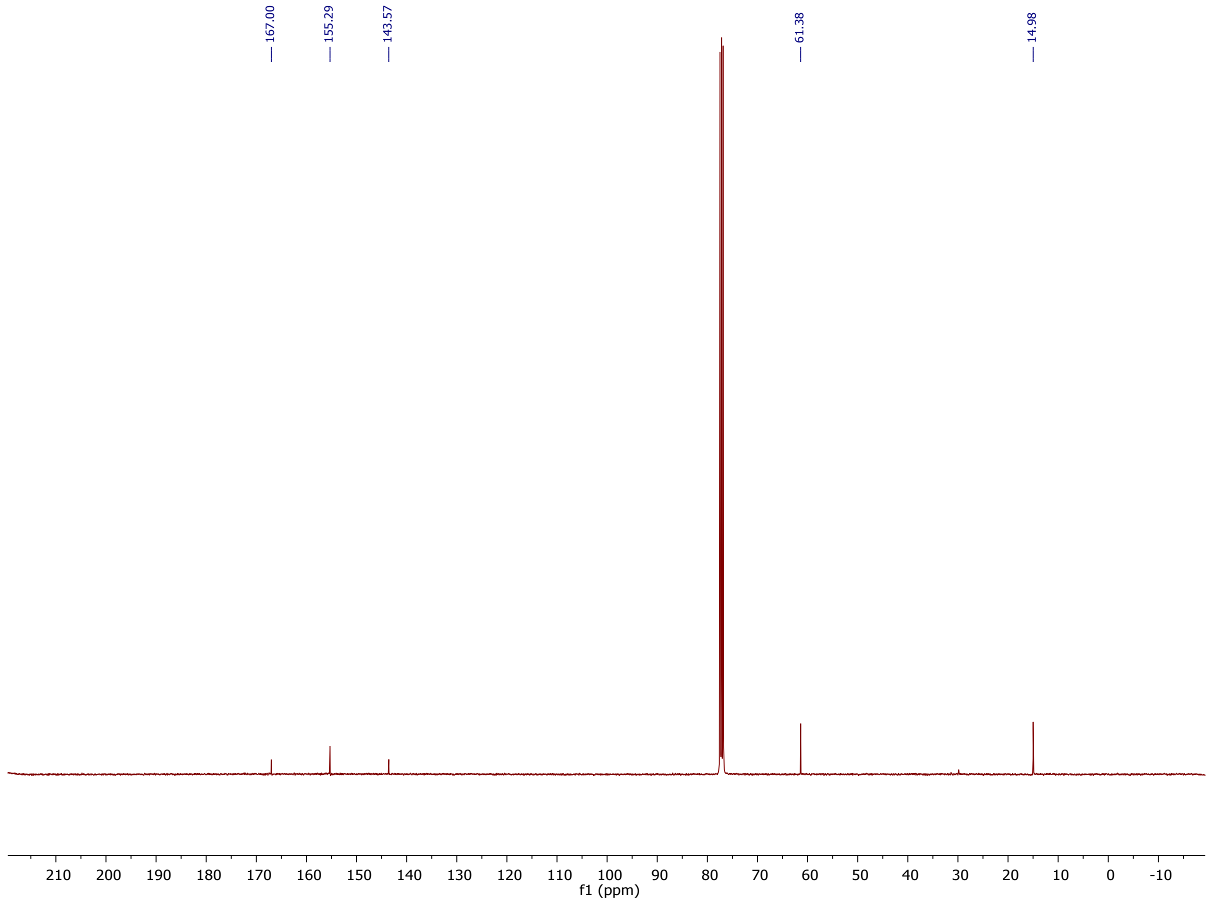
167.00
155.29
143.57
61.38
14.98
210
200
190
180
170
160
150
140
130
120
110
100
90
80
70
60
50
40
30
20
10
0
-10
f1 (ppm)

1.6.3 6-chloro-5-methoxy-4-((5-methyl-1H-pyrazol-3-yl)amino)-2-(methylsulfonyl) pyrimidine **11**

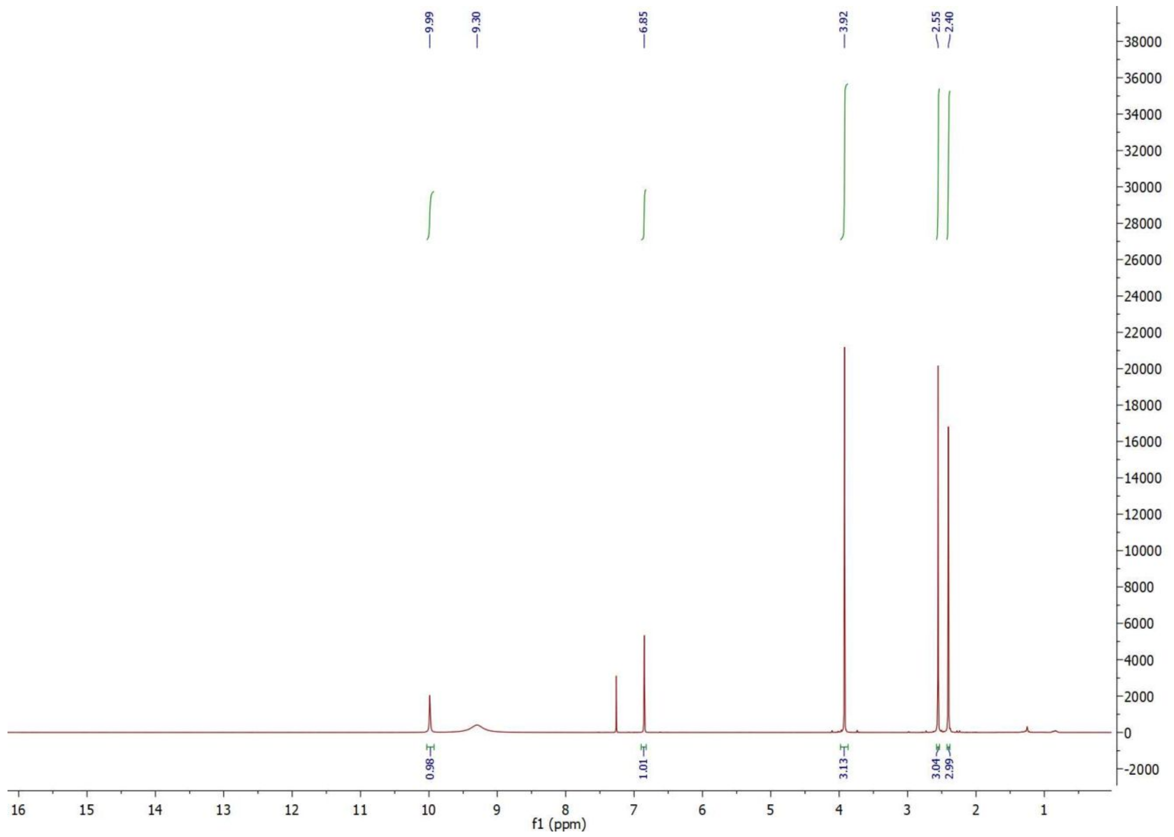

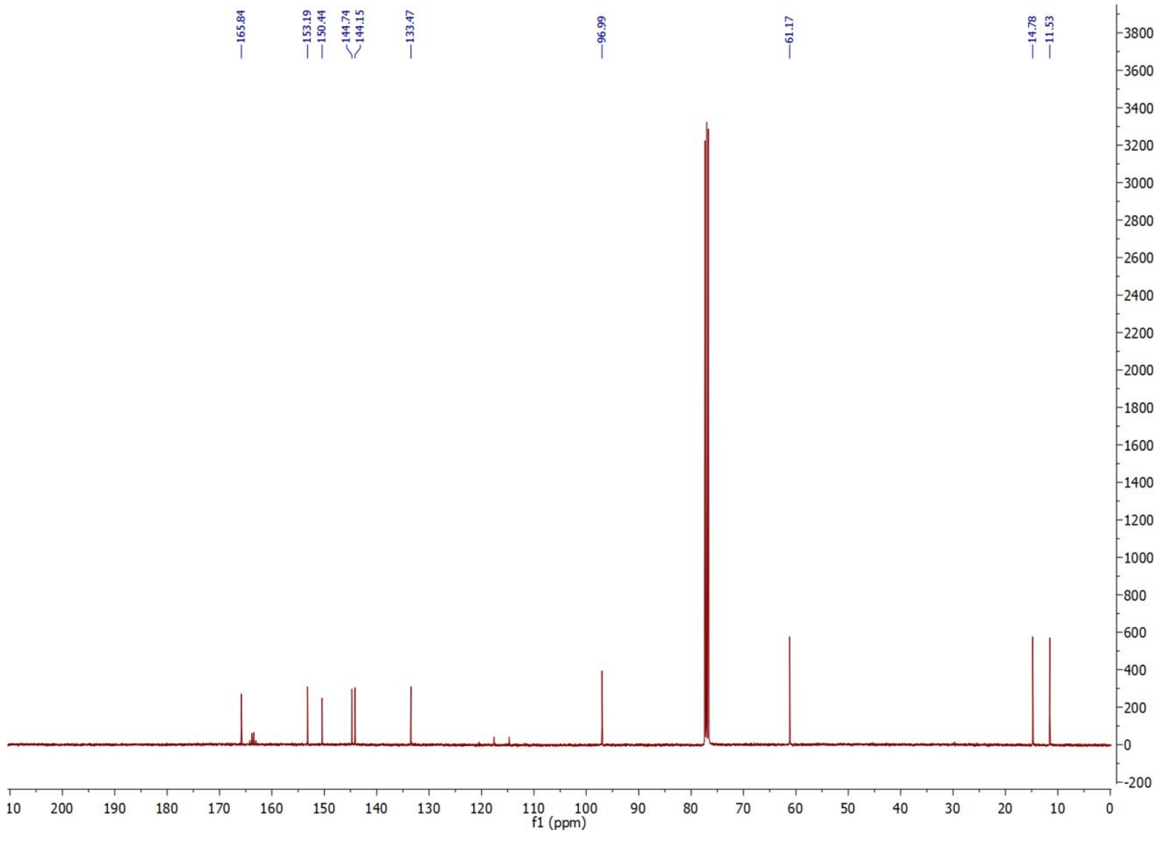

1.6.4 6-chloro-5-methoxy-4-((5-methyl-1H-pyrazol-3-yl)amino)-2-(methylsulfonyl) pyrimidine **12**

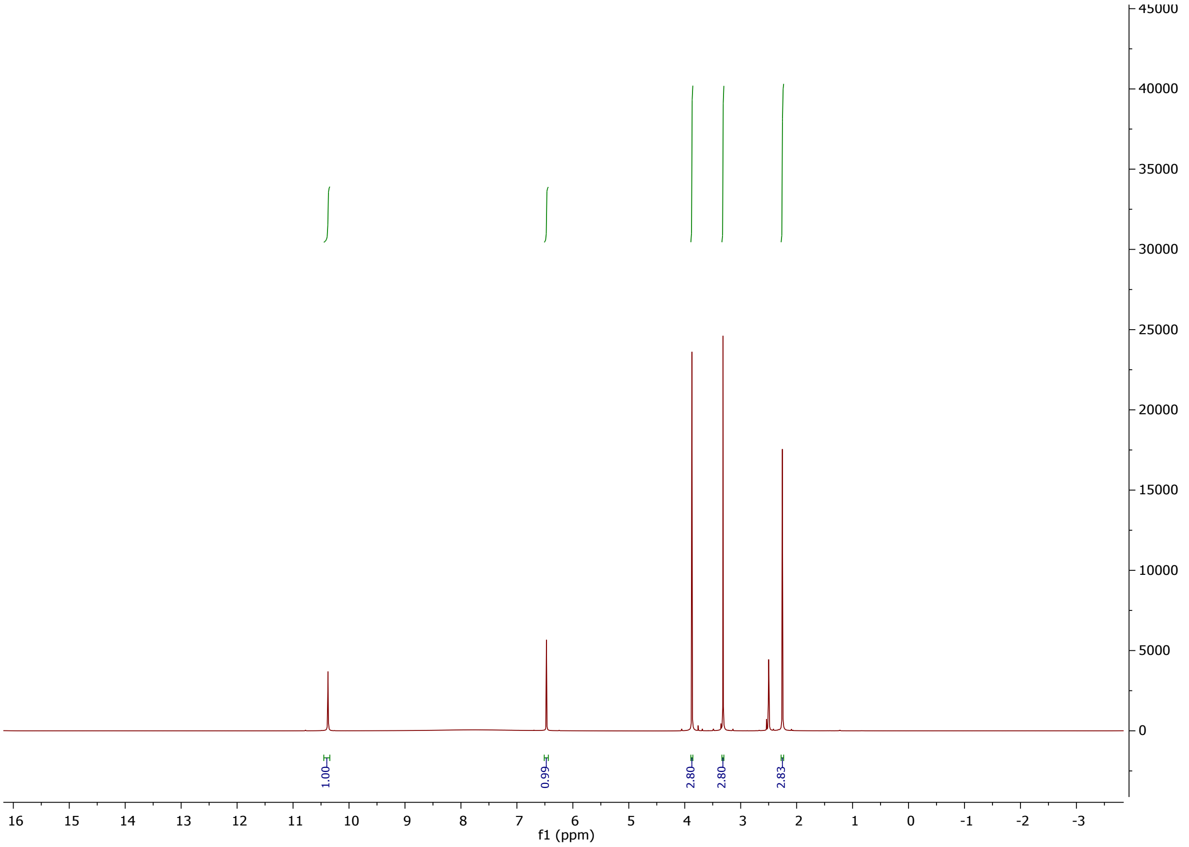

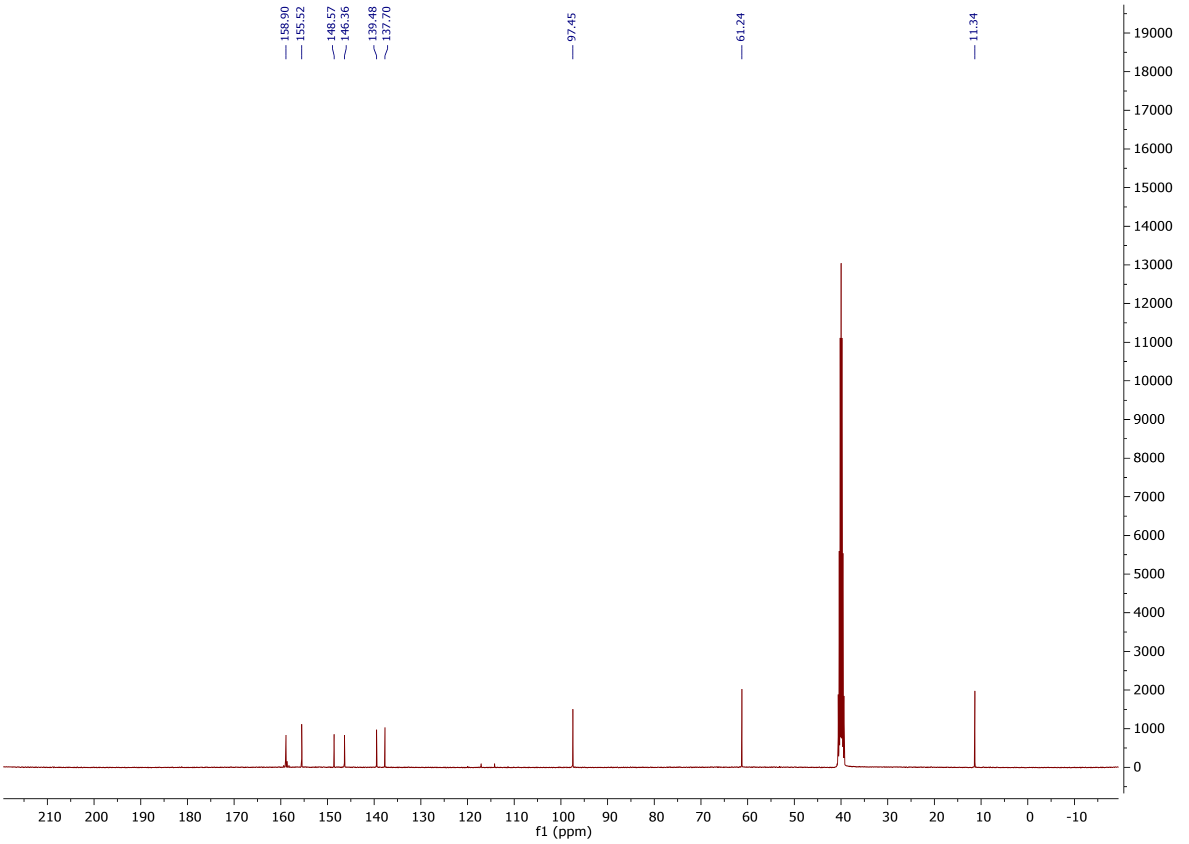

1.6.5 1-(bromomethyl)-2-fluoro-3-nitrobenzene **16**

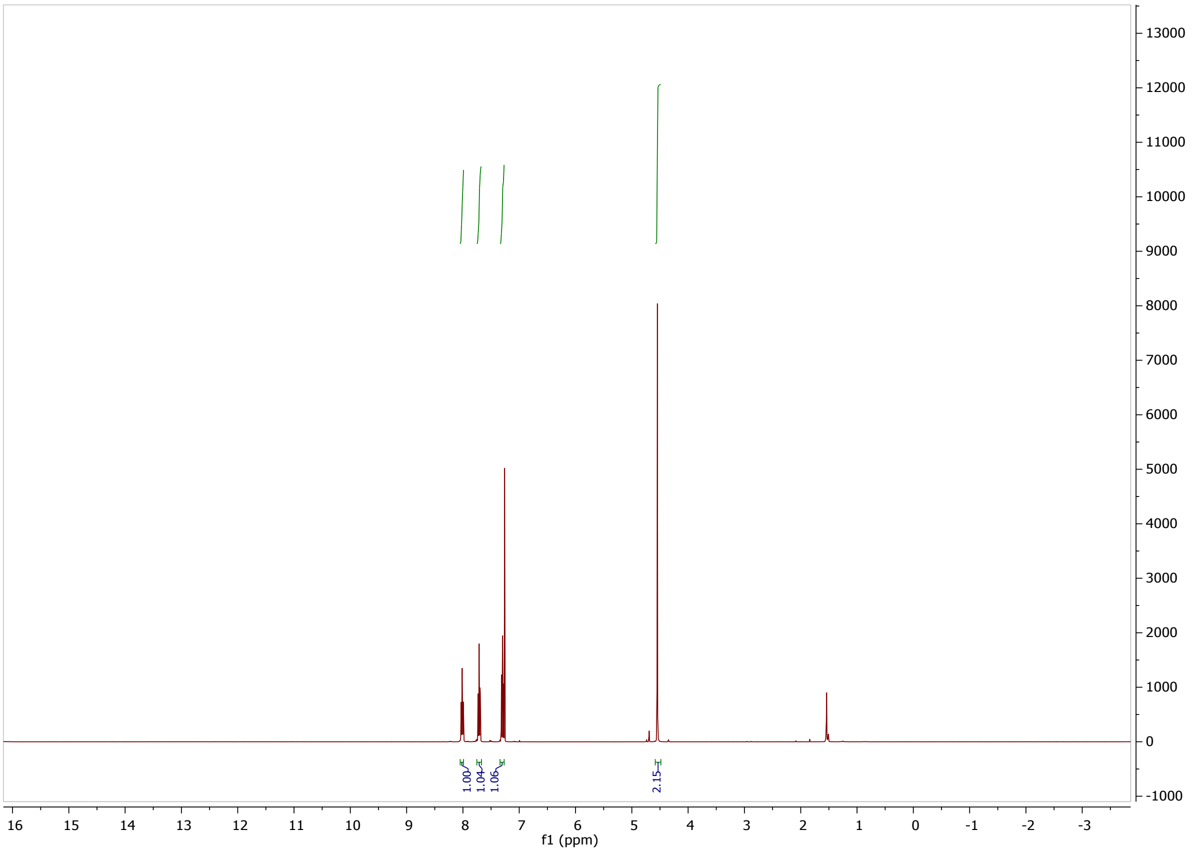

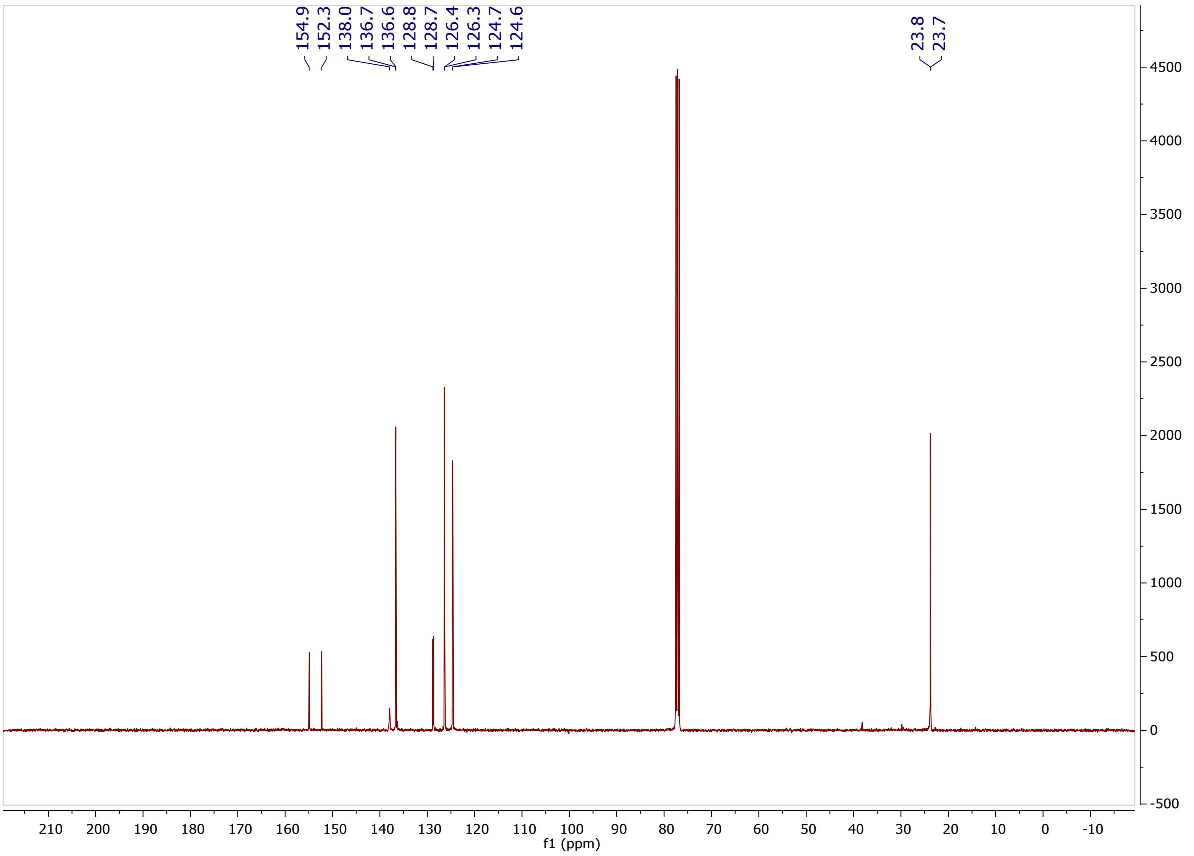

1.6.6 4-bromo-2-fluoro-1-(*tert*-butylthio) benzene **14**

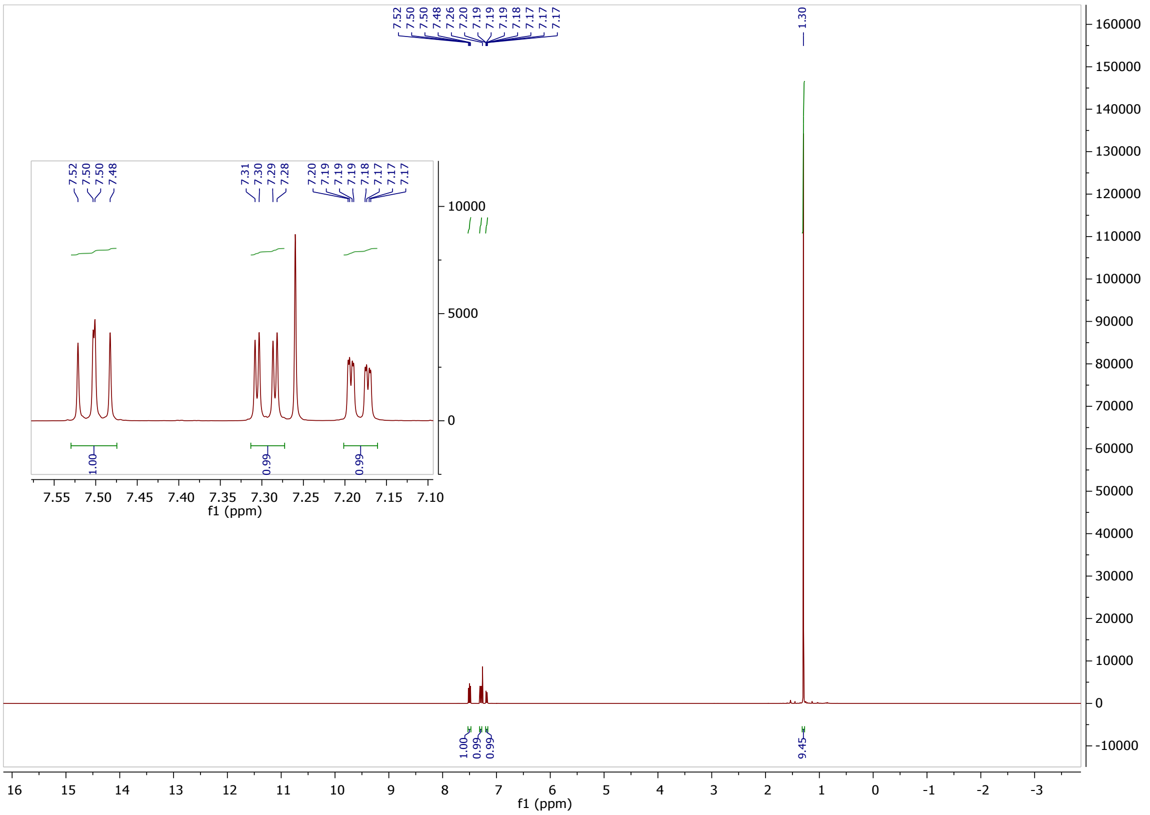

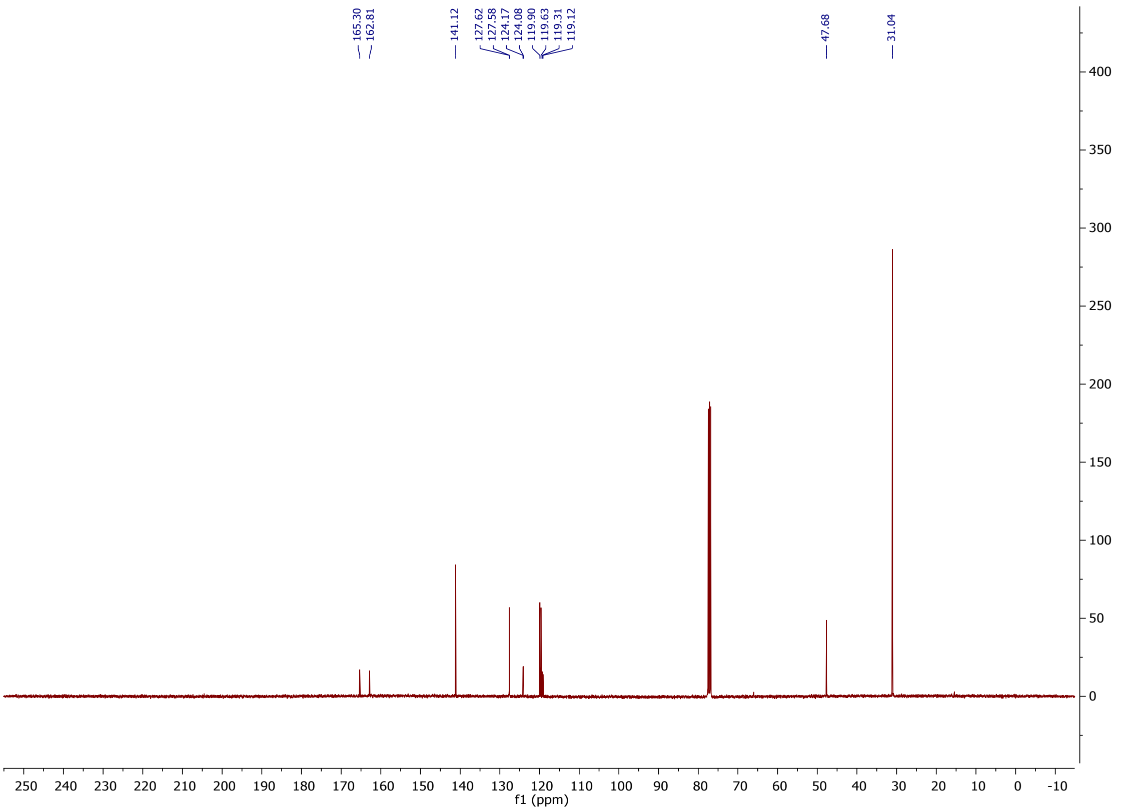

1.6.7 *tert*-Butyl (2-fluoro-4-((2-fluoro-3-nitrobenzyl) sulfonyl) phenyl) sulfane **17**

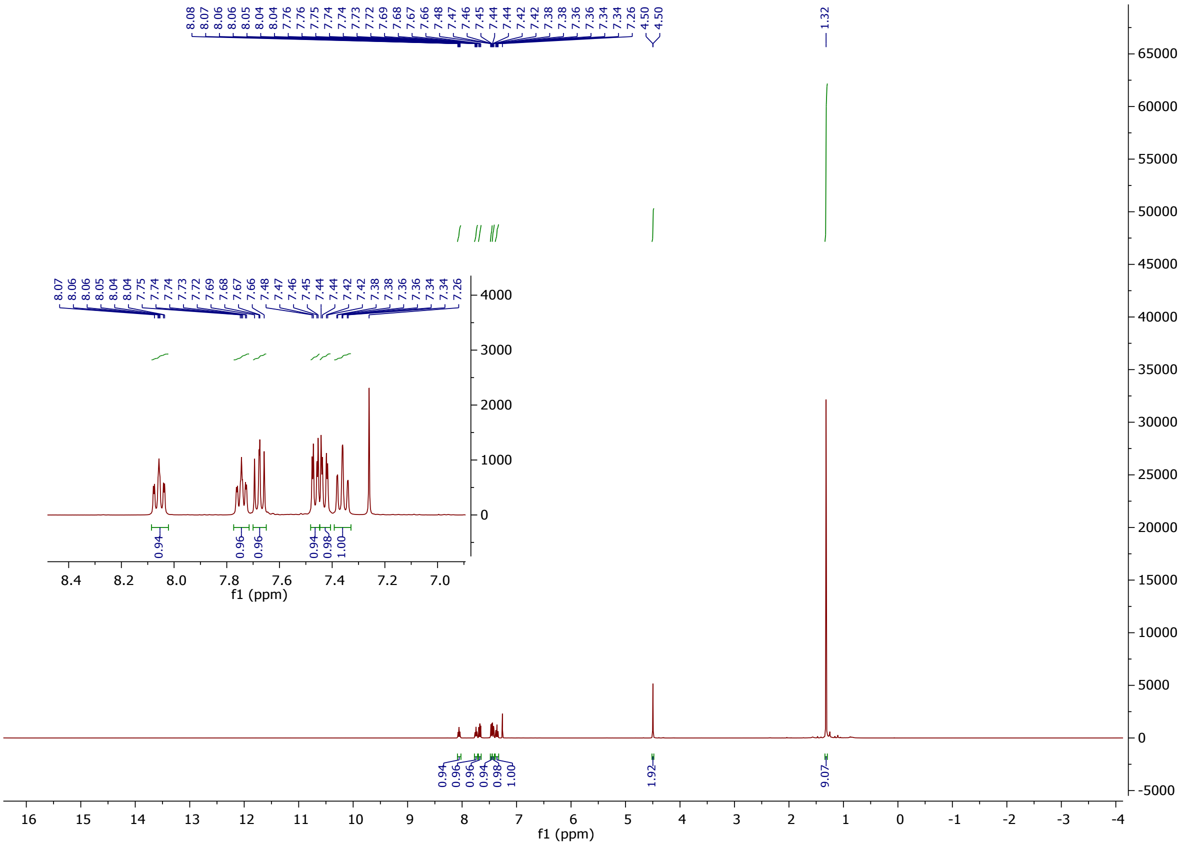

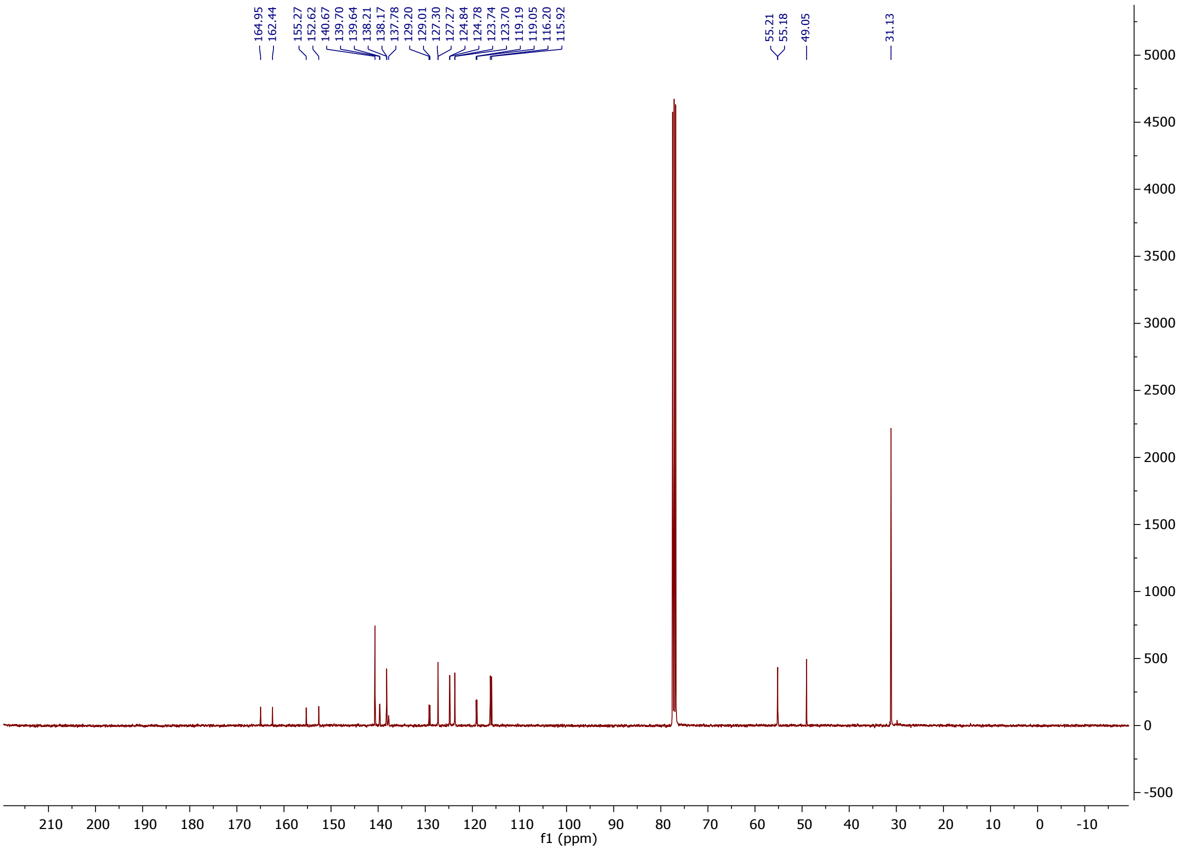

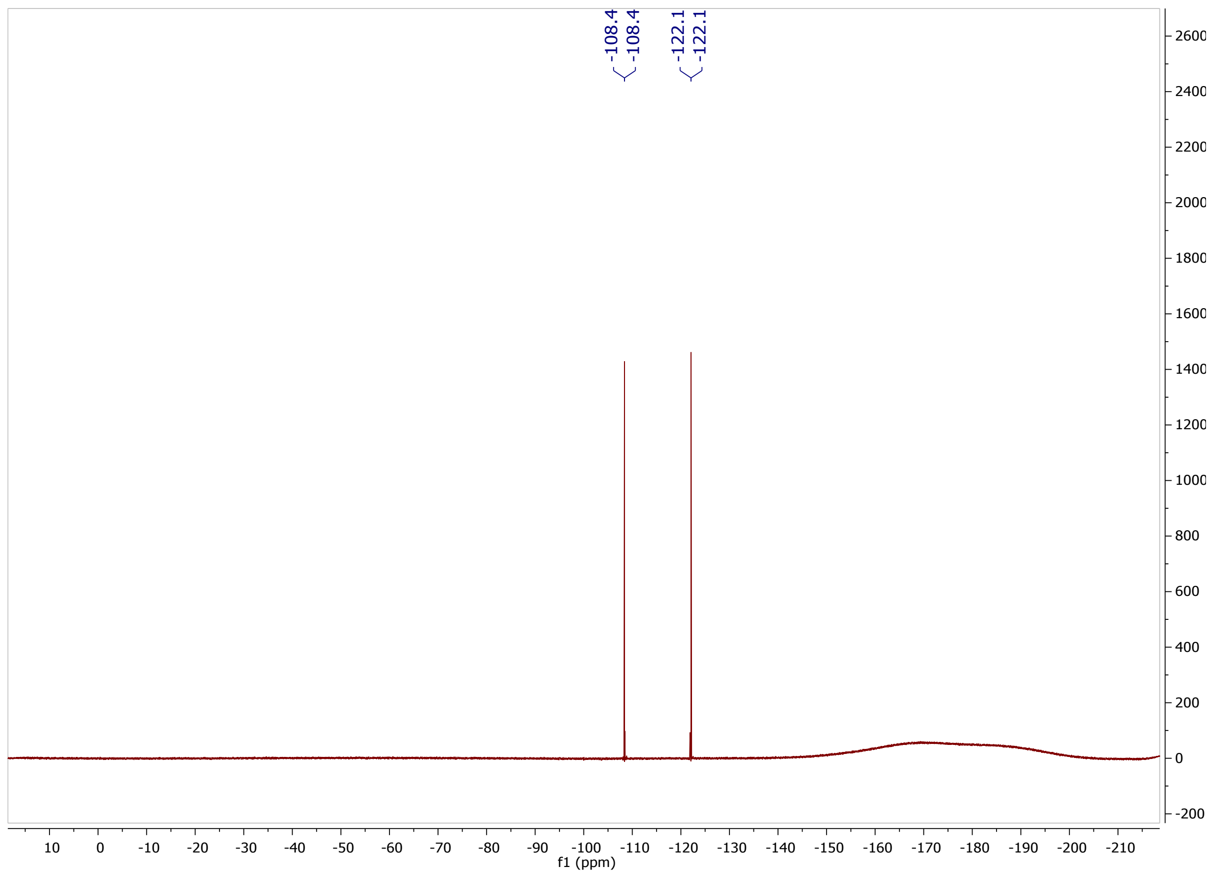
-108.4
-108.4
-122.1
-122.1
2600
2400
2200
2000
1800
1600
1400
1200
1000
800
600
400
200
0
-200
10 0 -10 -20 -30 -40 -50 -60 -70 -80 -90 -100 -110 -120 -130 -140 -150 -160 -170 -180 -190 -200 -210
f1 (ppm)

1.6.8 6-chloro-2-((2-fluoro-4-((2-fluoro-3-nitrobenzyl)sulfonyl)phenyl)thio)-5-methoxy-4-((5-methyl-1H-pyrazol-3-yl)amino) pyrimidine **19**

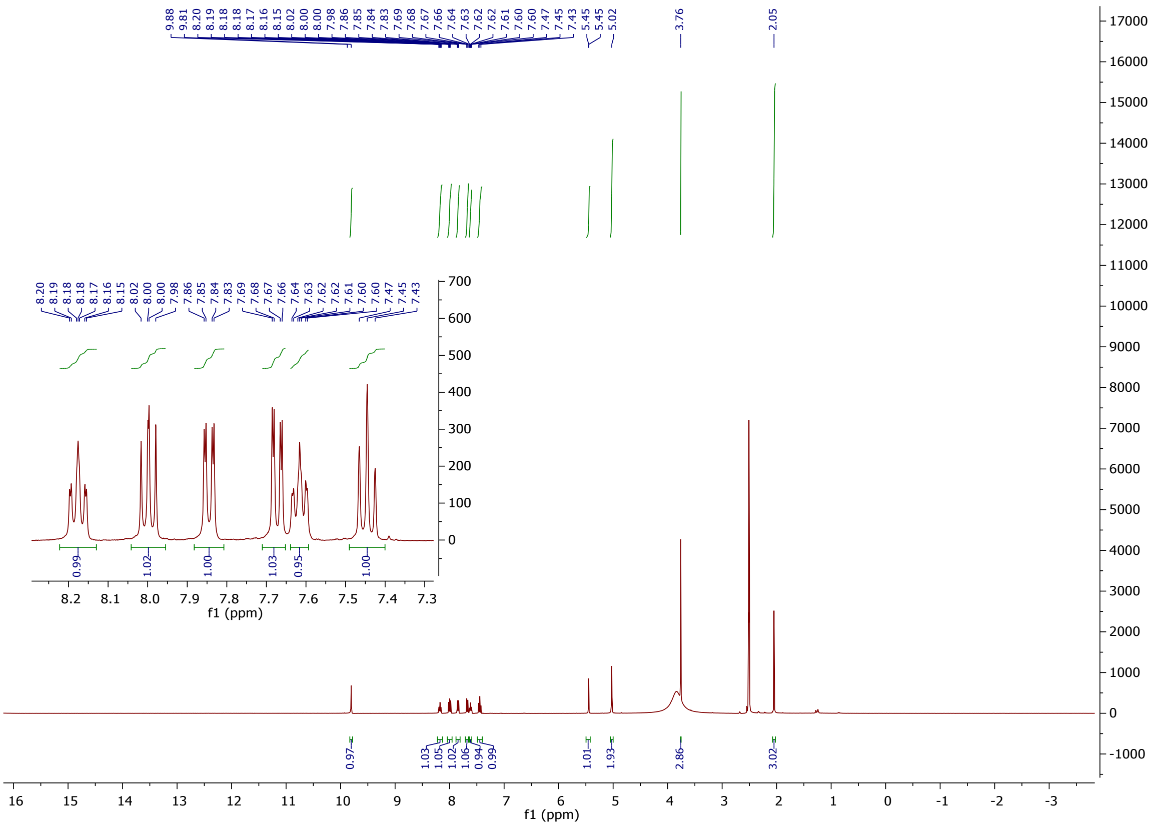

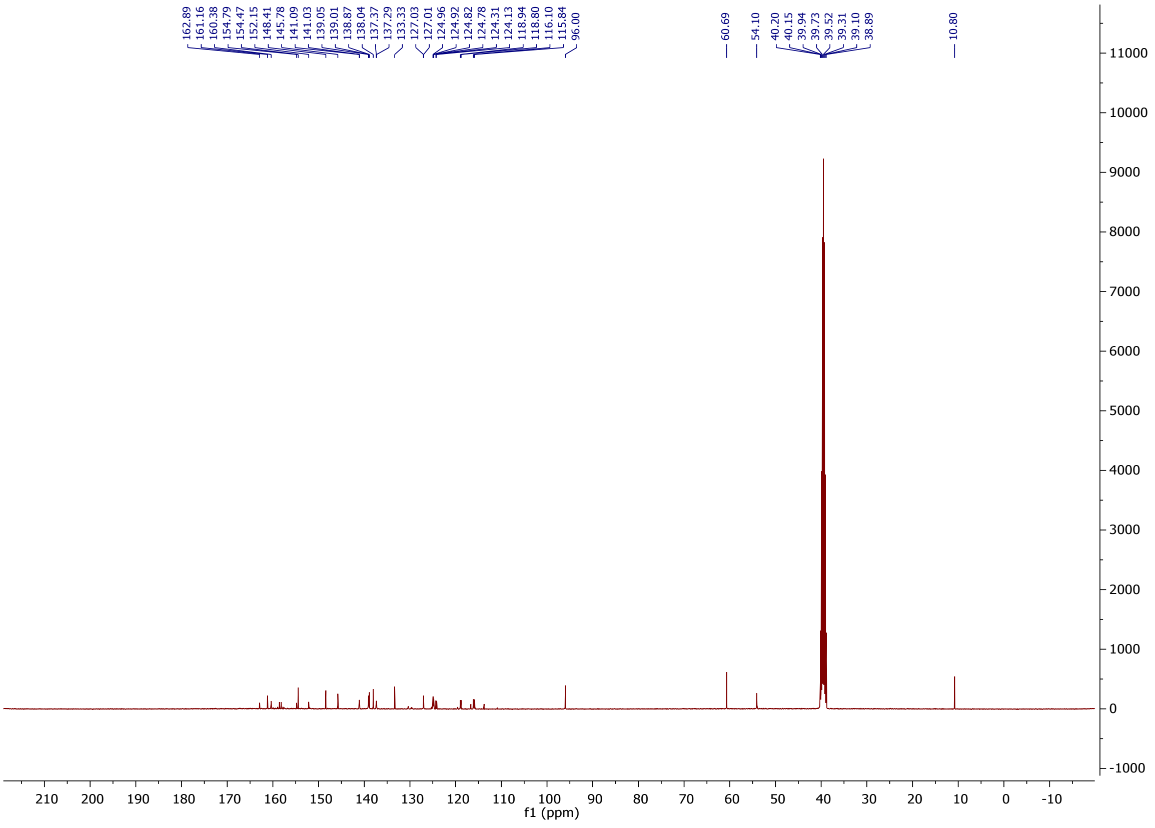

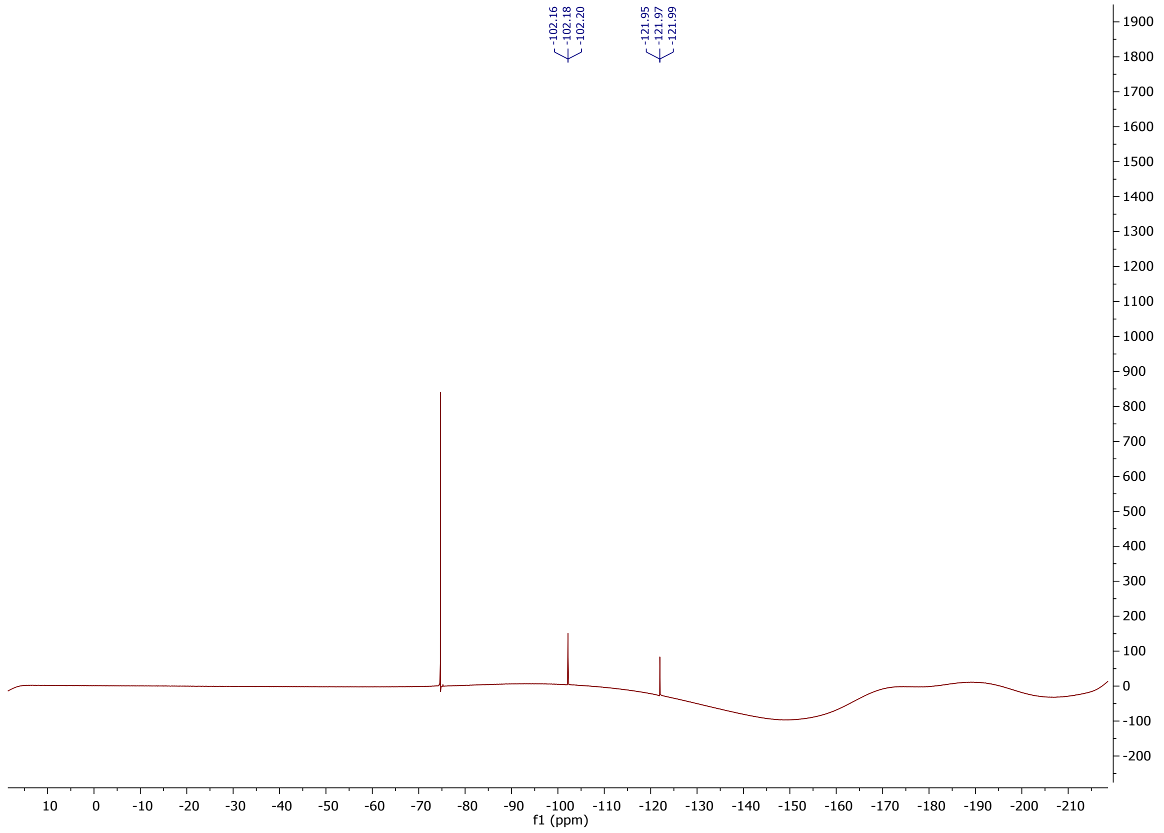
-102.16
-102.18
-102.20
-121.95
-121.97
-121.99
f1 (ppm)

1.6.9 *tert*-Butyl 2-(4-(2-((2-fluoro-4-((2-fluoro-3-nitrobenzyl)sulfonyl)phenyl)thio)-5-methoxy-6-((5-methyl-1H-pyrazol-3-yl)amino)pyrimidin-4-yl)piperazin-1-yl)acetate **21a**

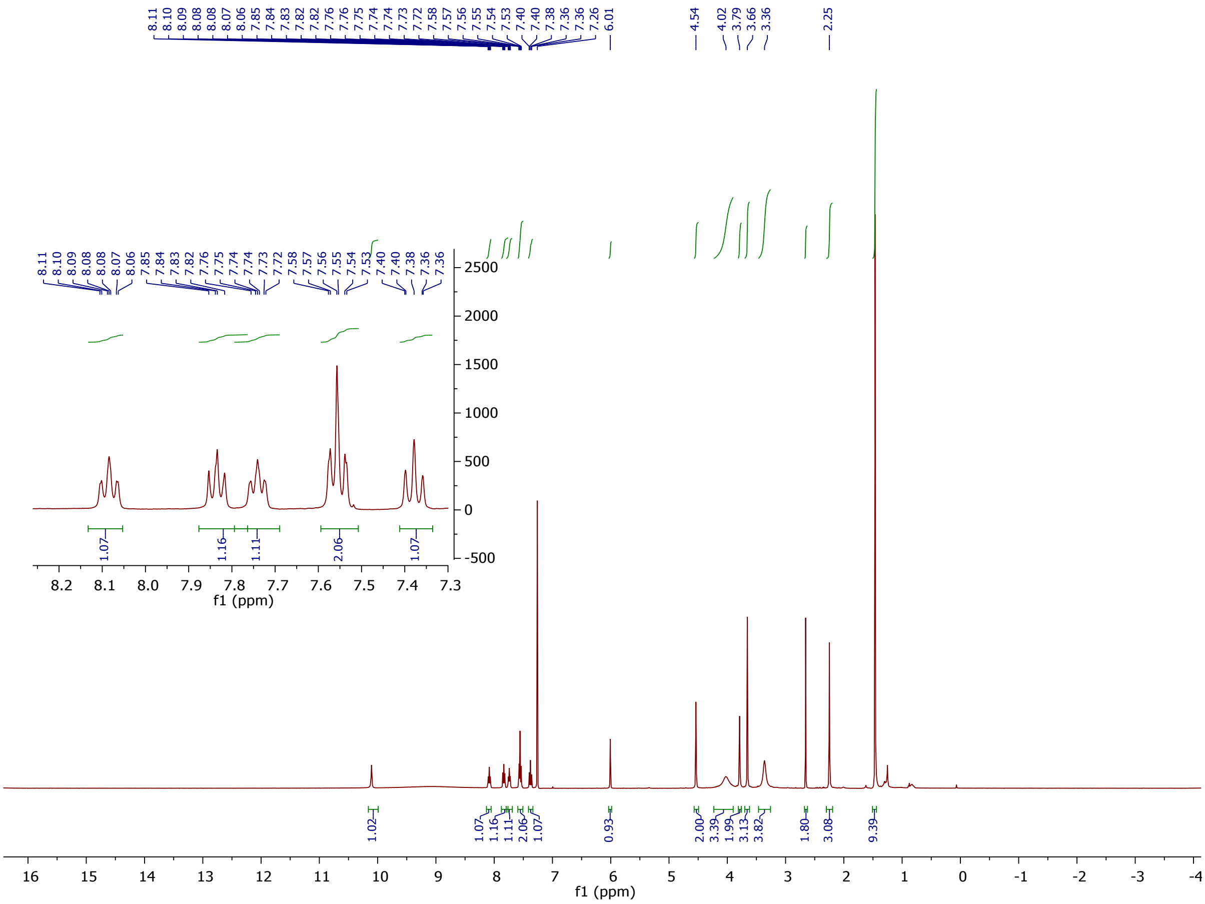

1.6.10 2-(4-(2-((2-fluoro-4-((2-fluoro-3-nitrobenzyl)sulfonyl)phenyl)thio)-5-methoxy-6-((5-methyl-1H-pyrazol-3-yl)amino)pyrimidin-4-yl)piperazin-1-yl)acetic acid **21b**

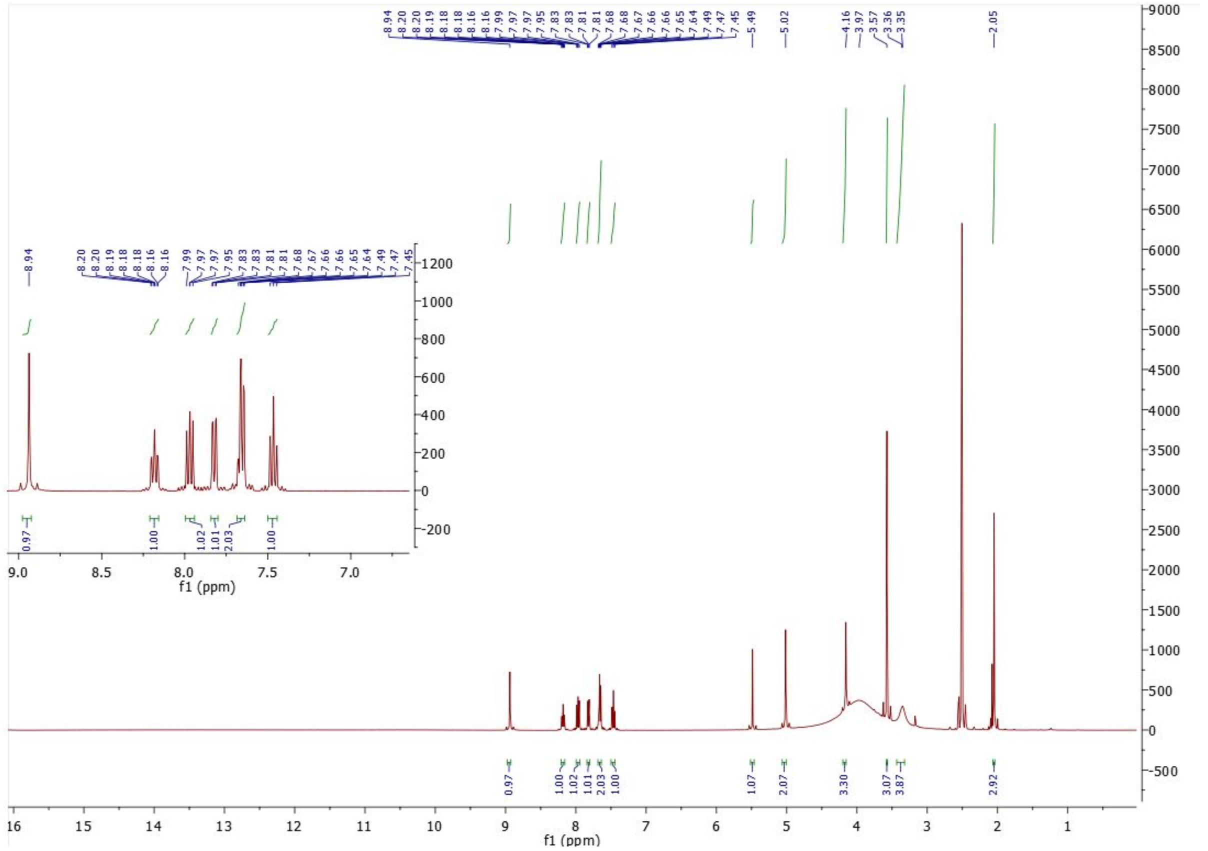

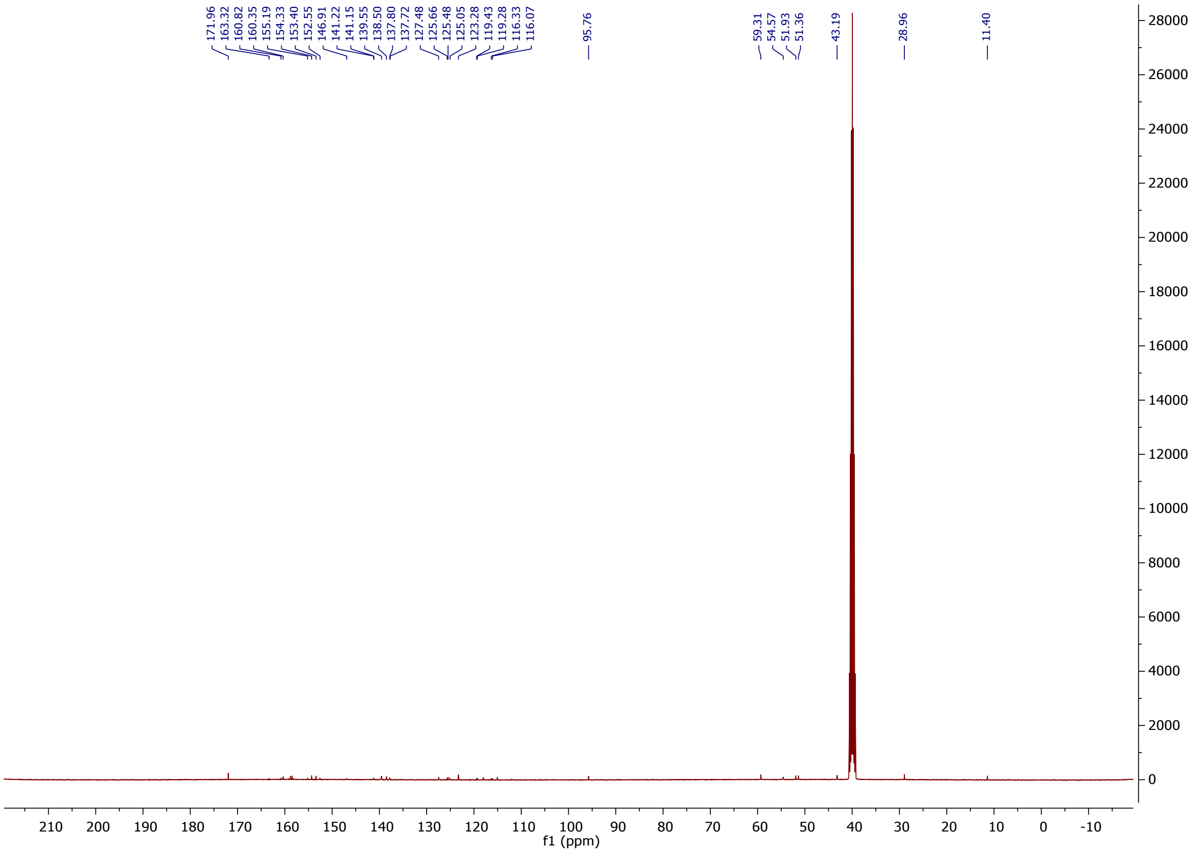

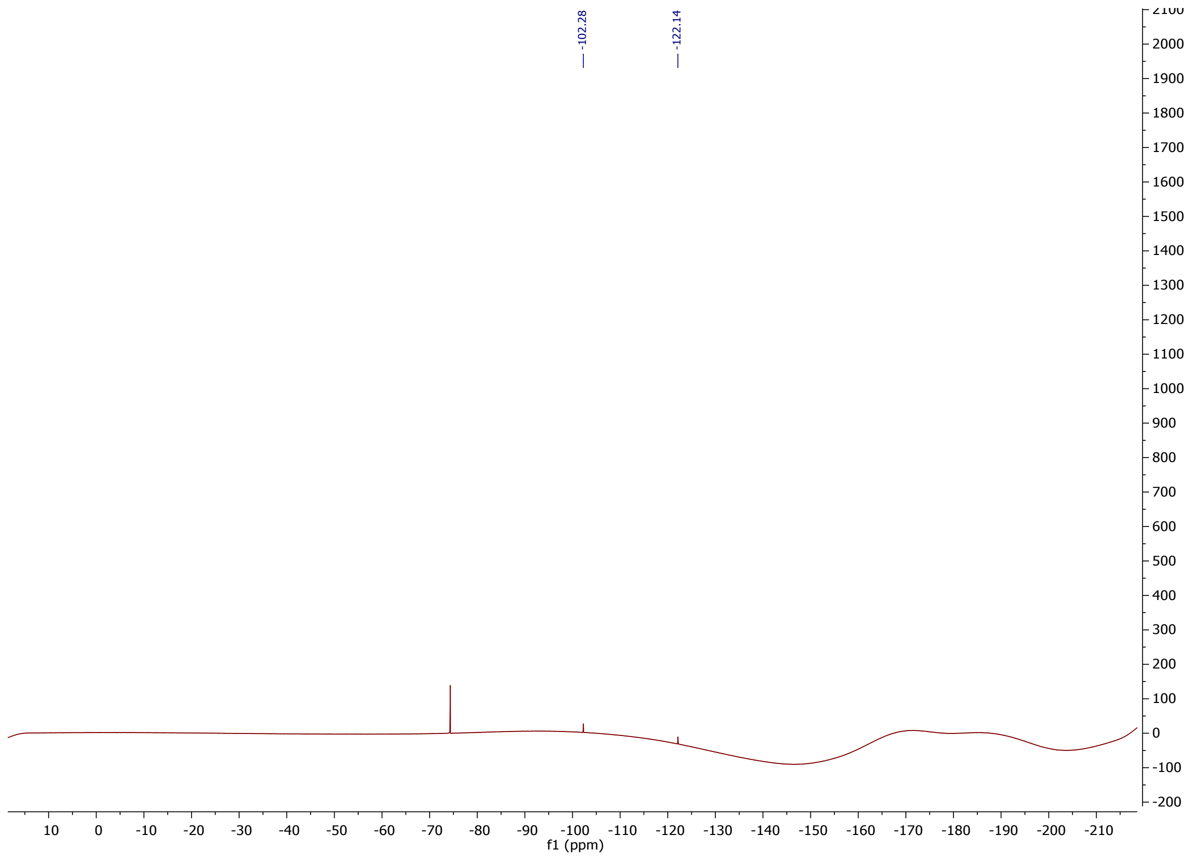

-102.28
-122.14
f1 (ppm)

1.6.11 2-(1-(2-((2-fluoro-4-((2-fluoro-3-nitrobenzyl)sulfonyl)phenyl)thio)-5-methoxy-6-((5-methyl-1H-pyrazol-3-yl)amino)pyrimidin-4-yl)piperidin-4-yl) acetic acid **21c**

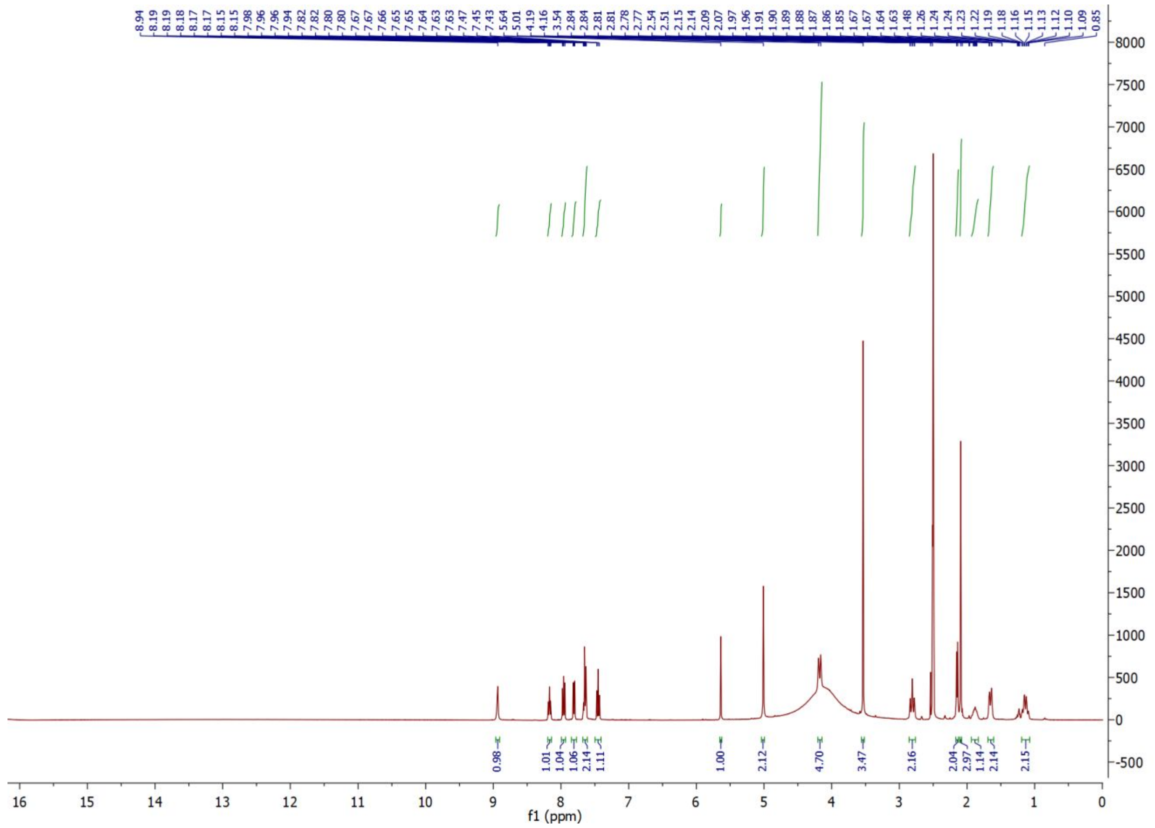

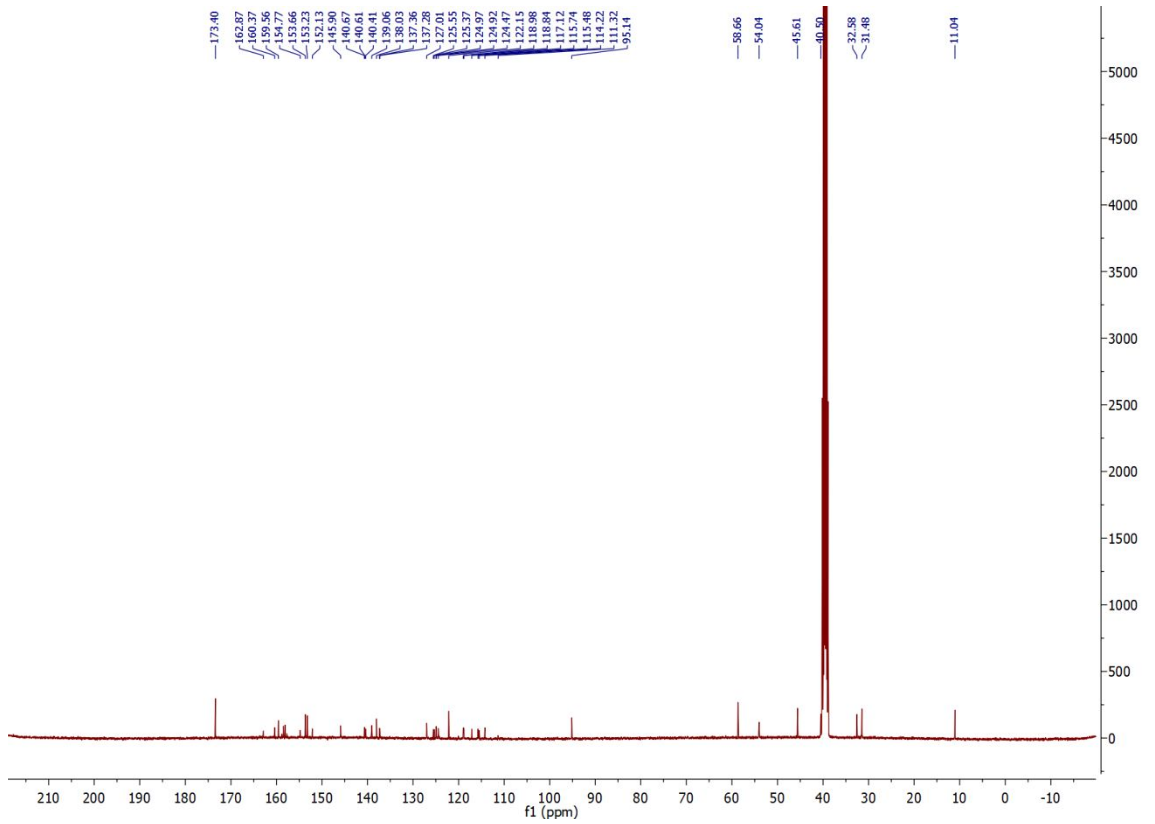

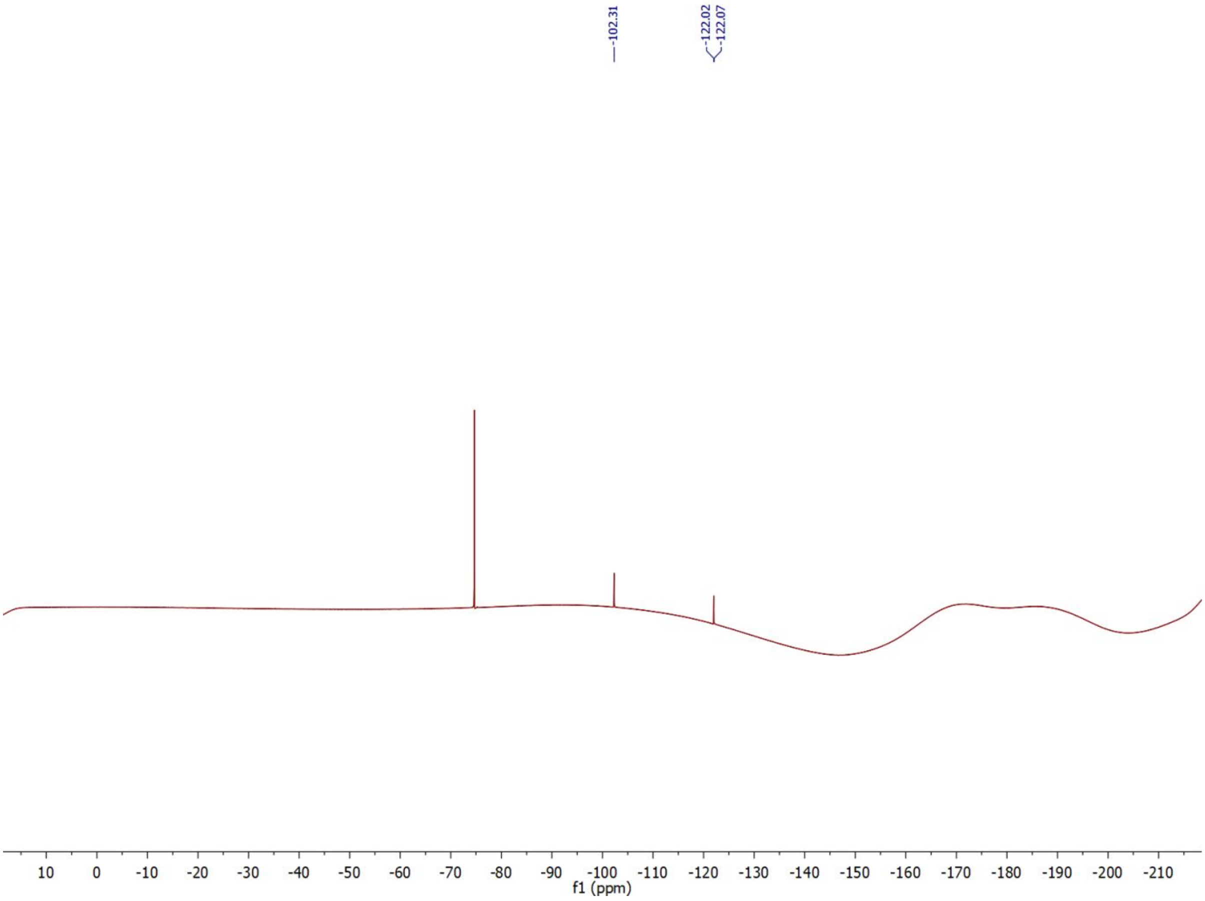

1.6.12 SiR595-Linkers **26a-c**

20a

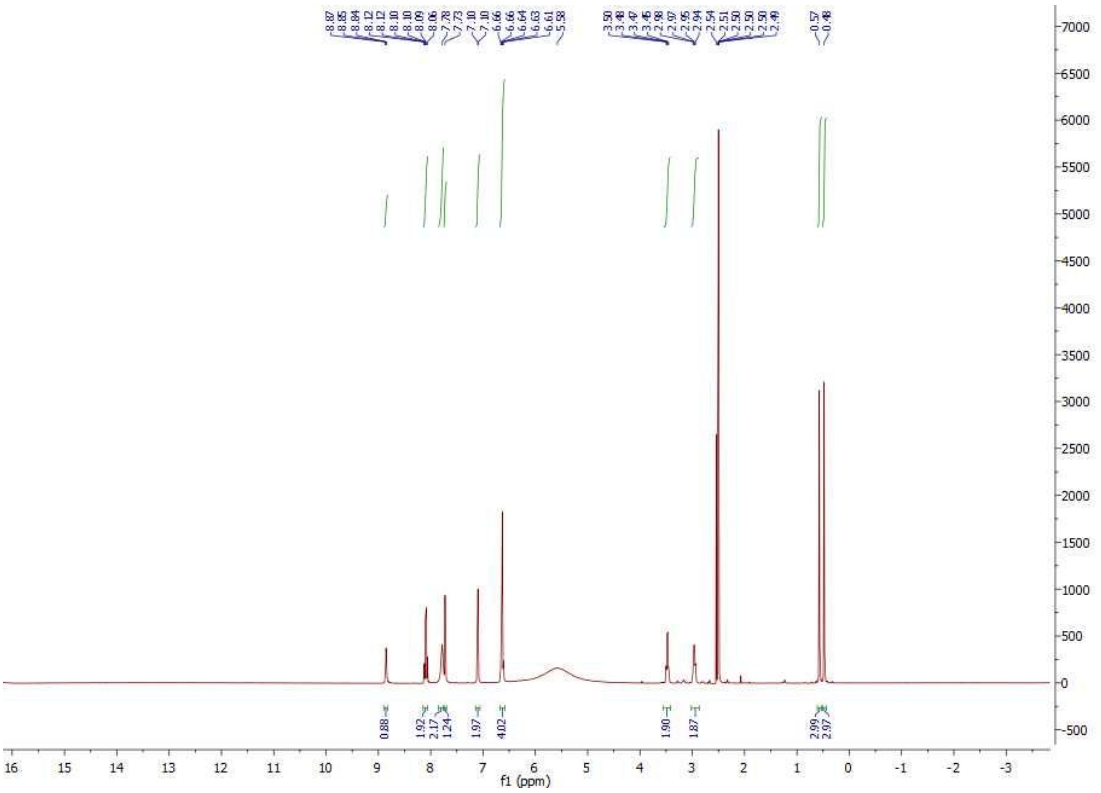

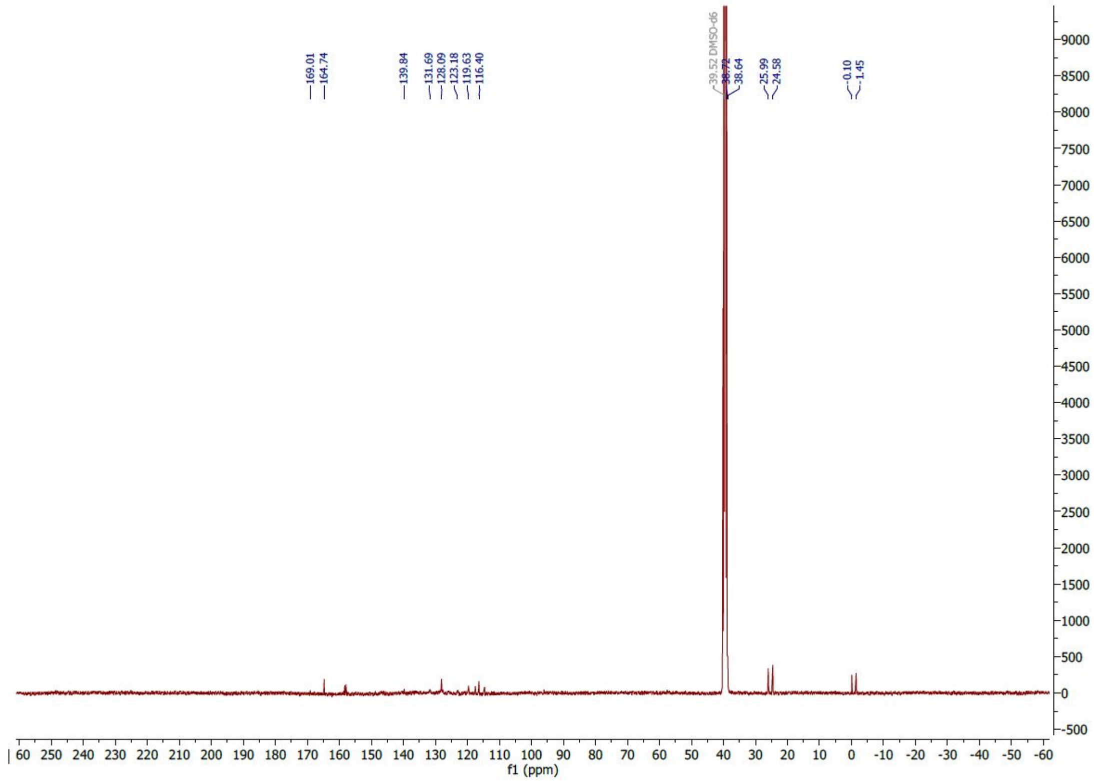

20b

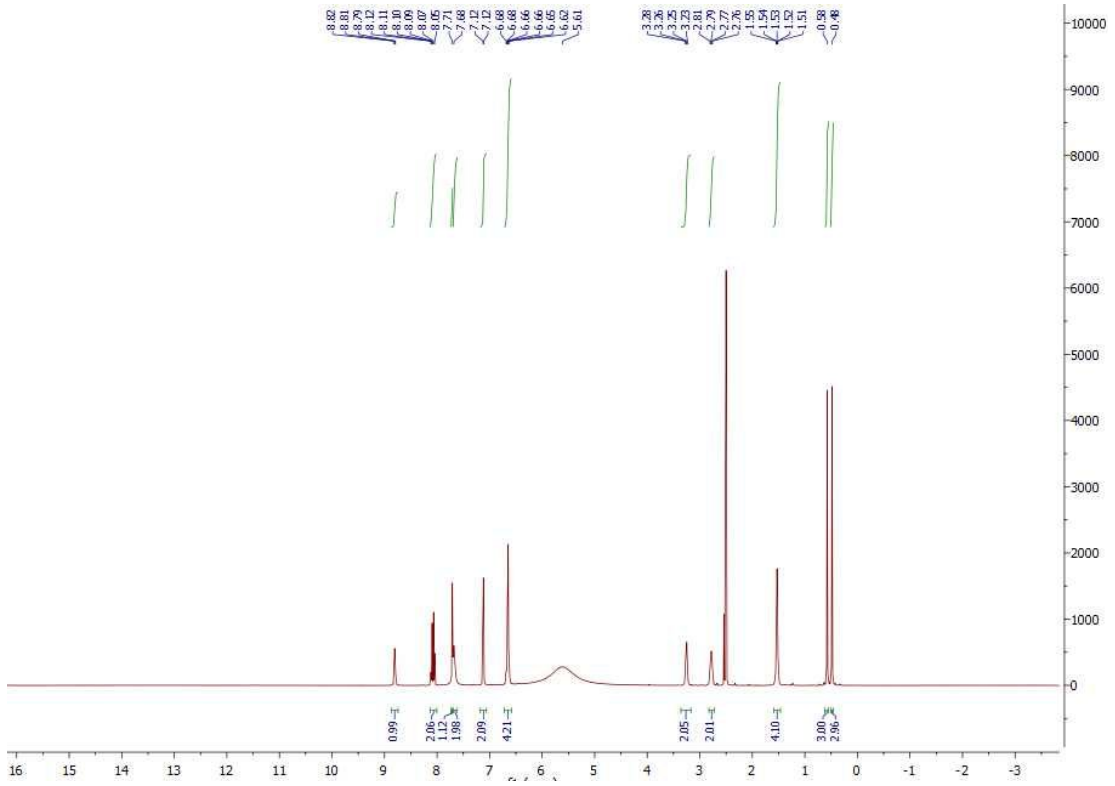

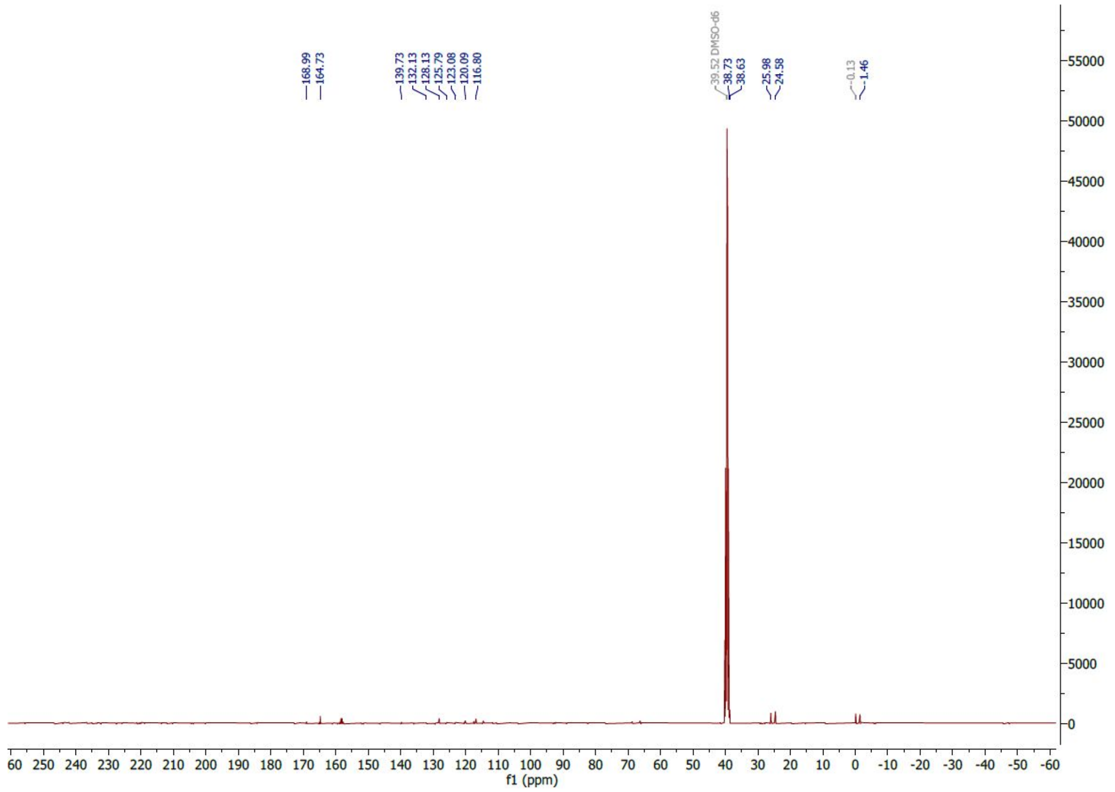

20c

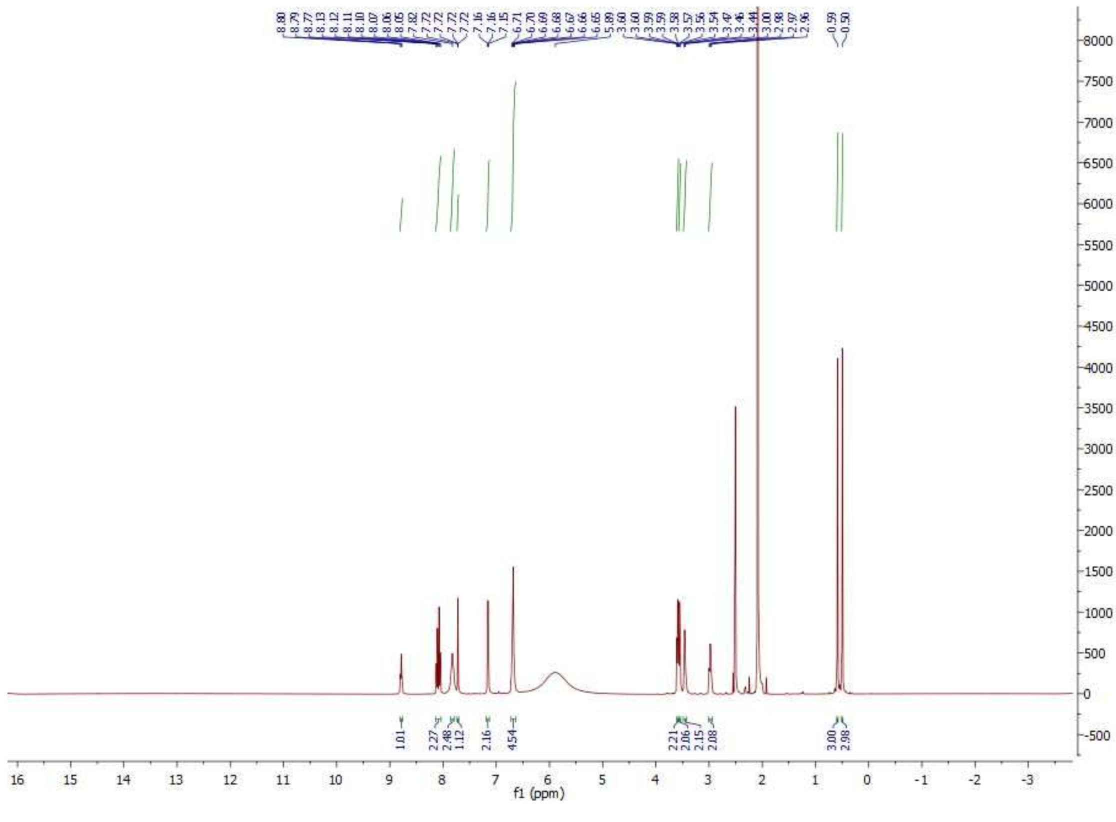

1.6.13 SiR595-linker-C-Cen probes (**2b-d**)

2b – SiR595-PEG-C-Cen

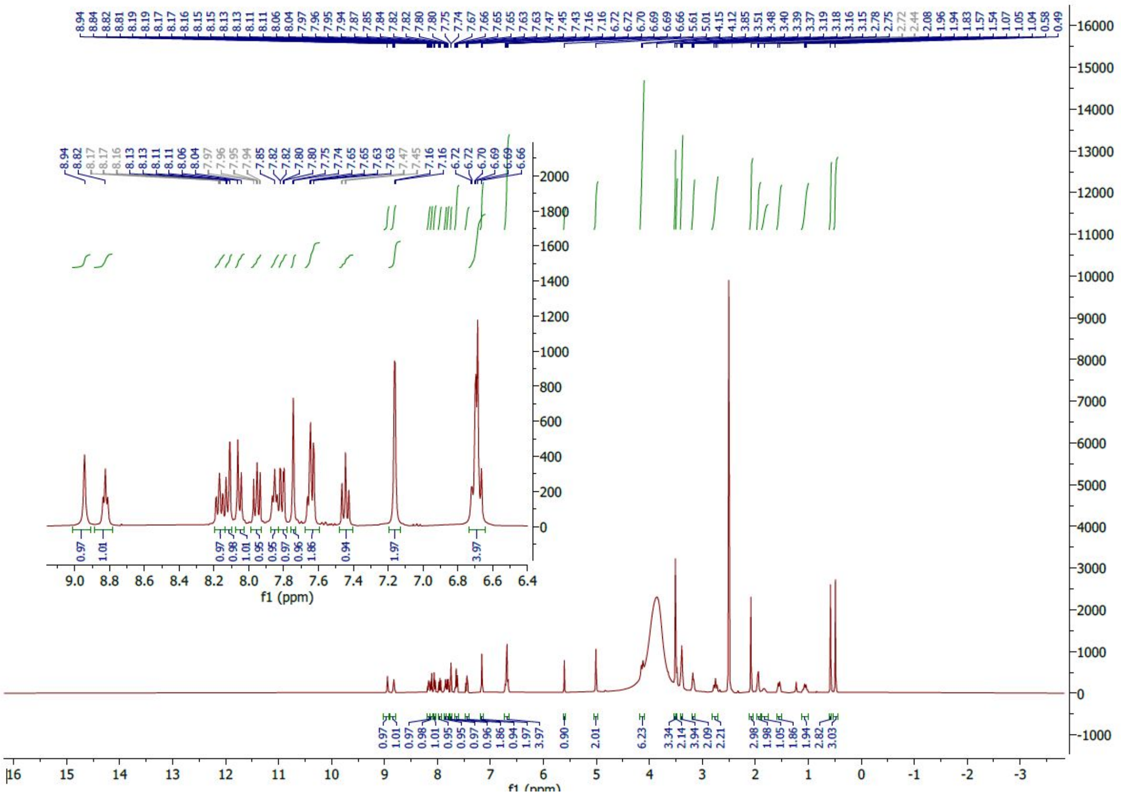

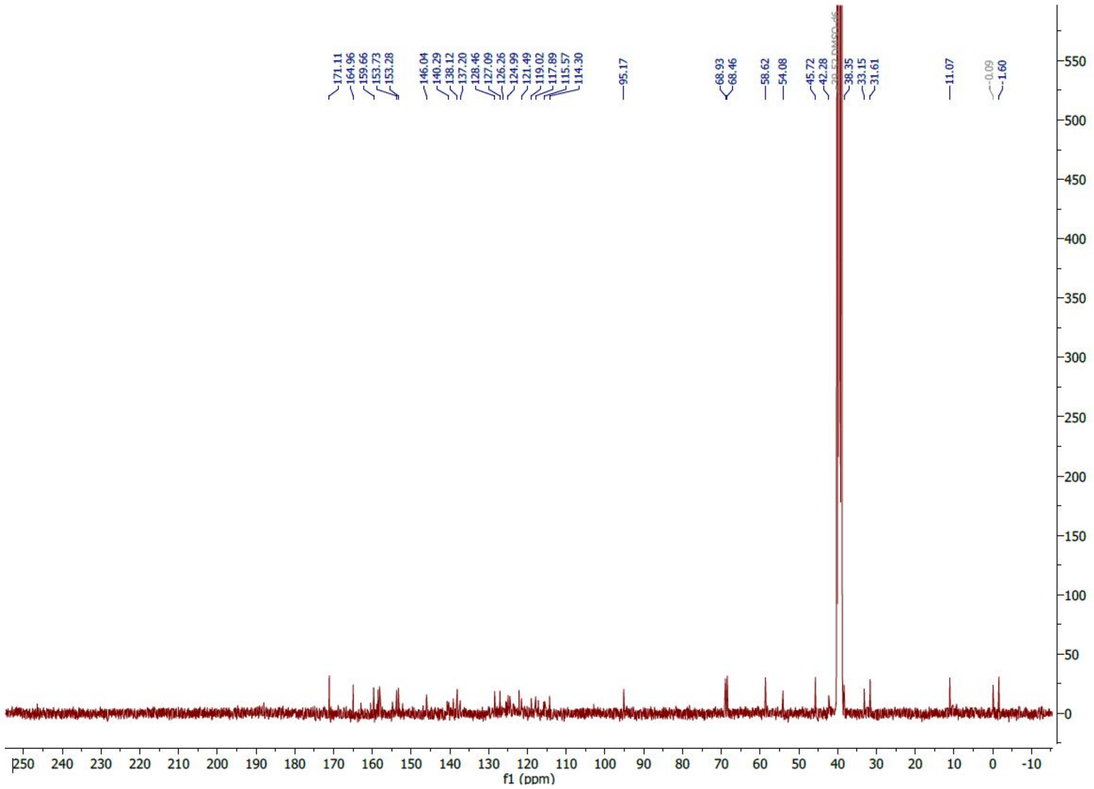

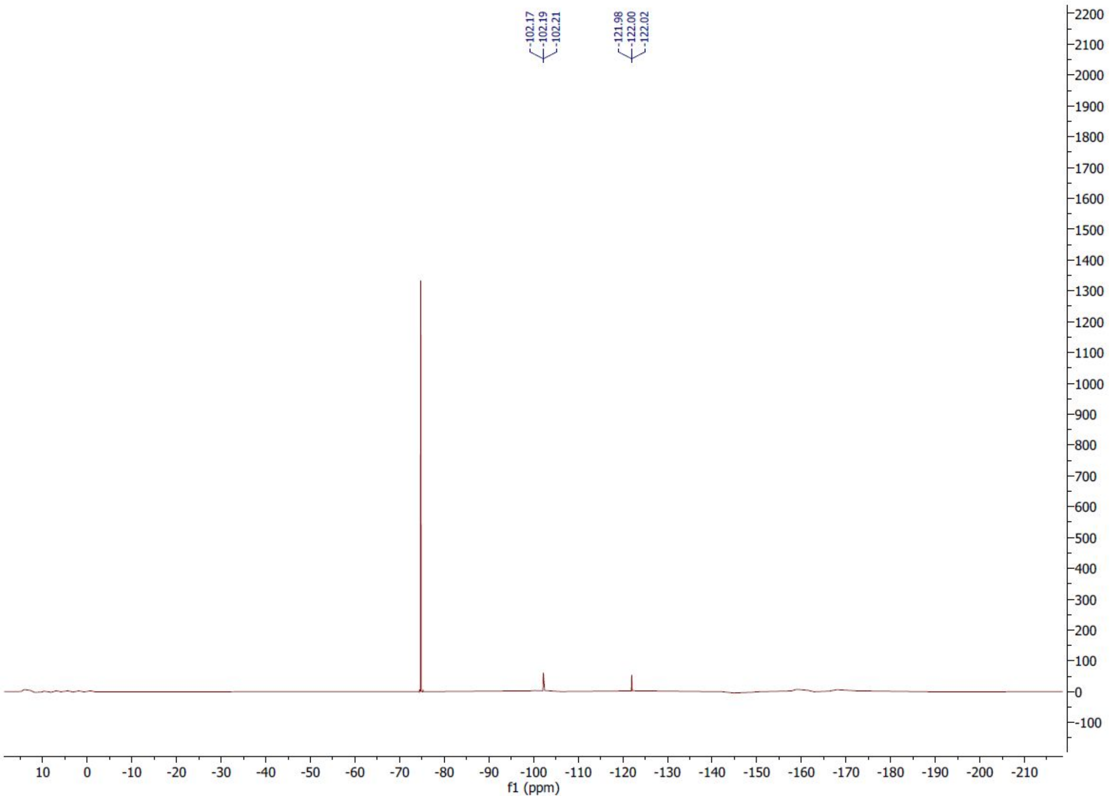
-102.17
-102.19
-102.21
-121.98
-122.00
-122.02
f1 (ppm)

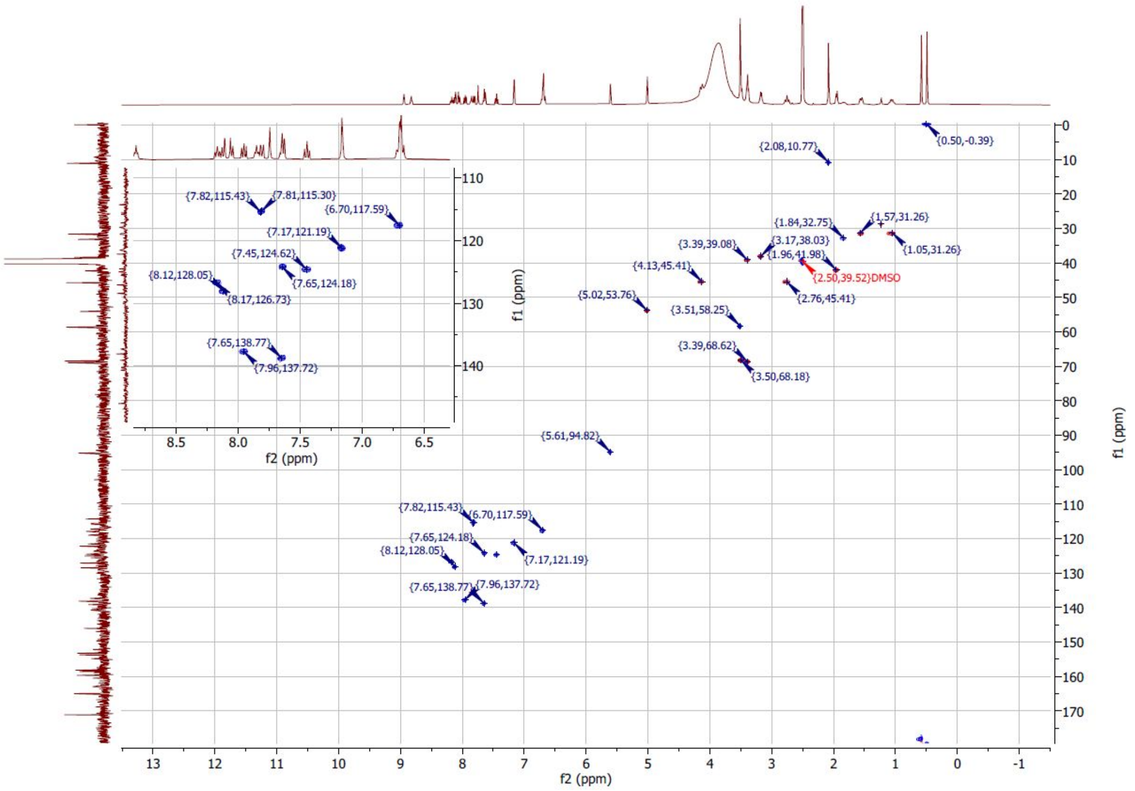
{7.82,115.43}
{7.81,115.30}
{6.70,117.59}
{7.17,121.19}
{7.45,124.62}
{7.65,124.18}
{8.12,128.05}
{8.17,126.73}
{7.65,138.77}
{7.96,137.72}
{0.50,-0.39}
{2.08,10.77}
{1.84,32.75}
{1.57,31.26}
{3.17,38.03}
{3.39,39.08}
{1.96,41.98}
{1.05,31.26}
{4.13,45.41}
{2.50,39.52}DMSO
{2.76,45.41}
{5.02,53.76}
{3.51,58.25}
{3.39,68.62}
{3.50,68.18}
{5.61,94.82}
f2 (ppm)
f1 (ppm)

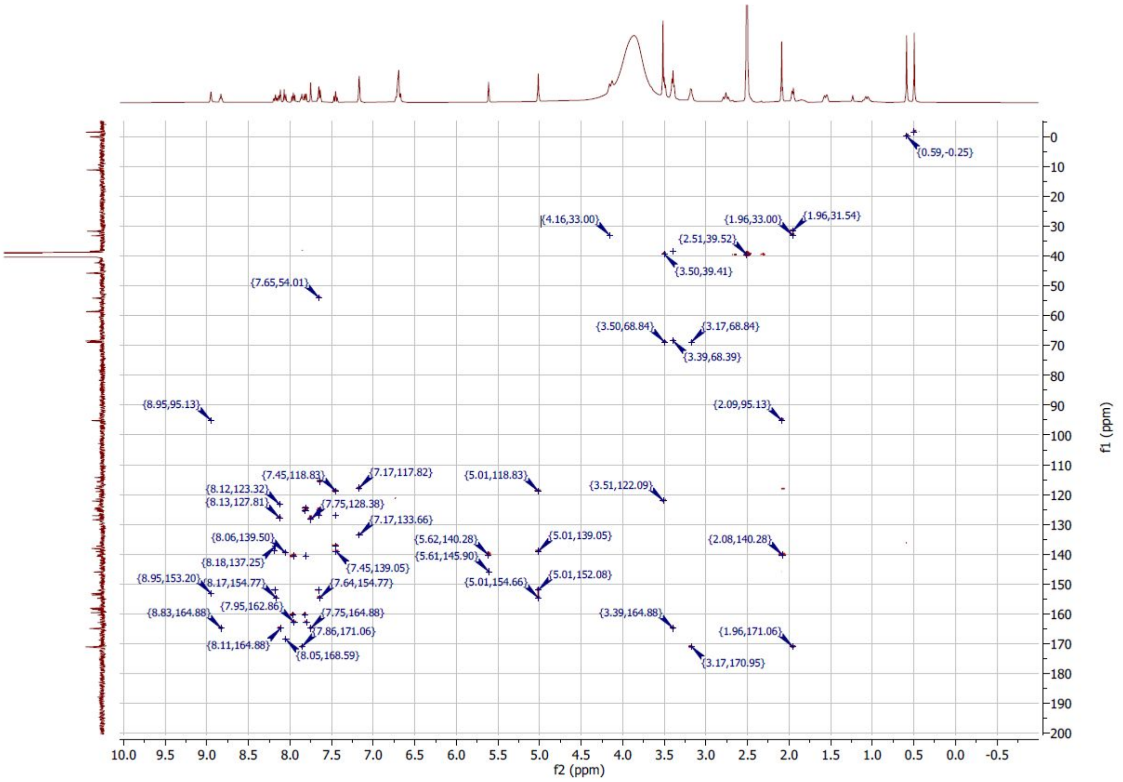

2c – SiR595-C4-C-Cen

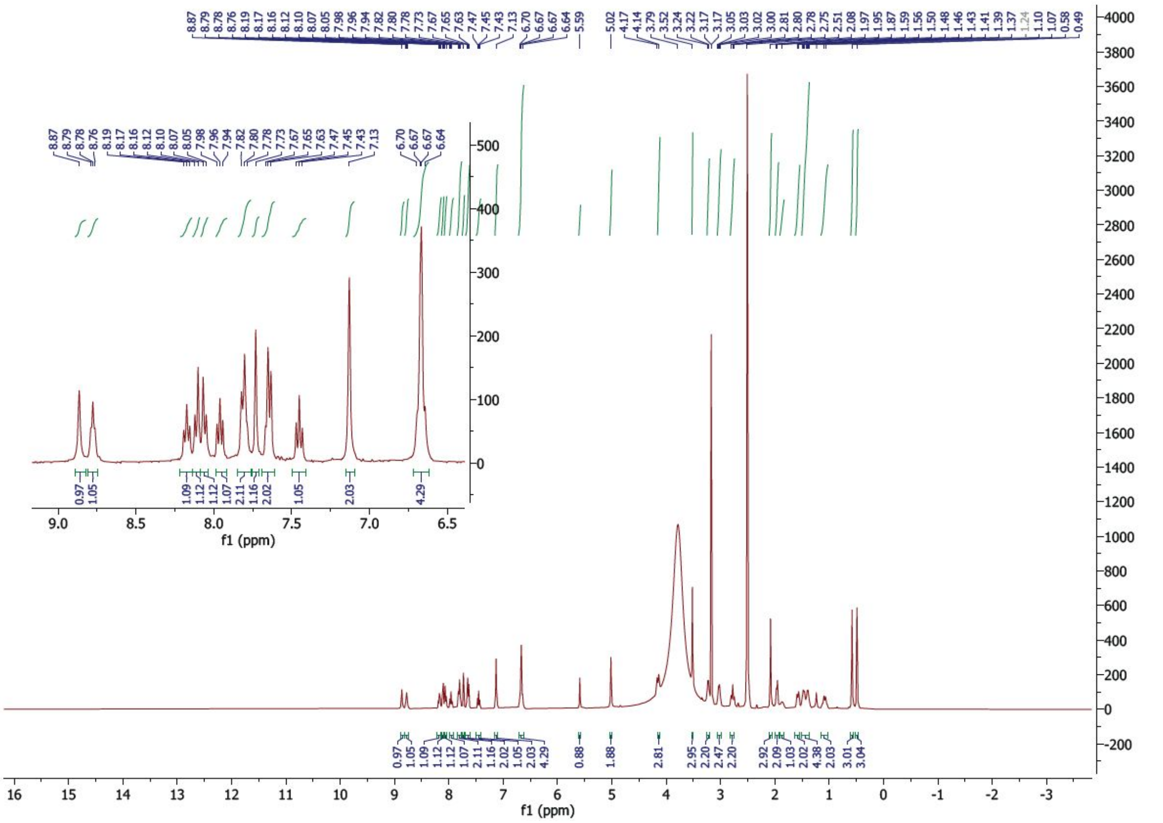

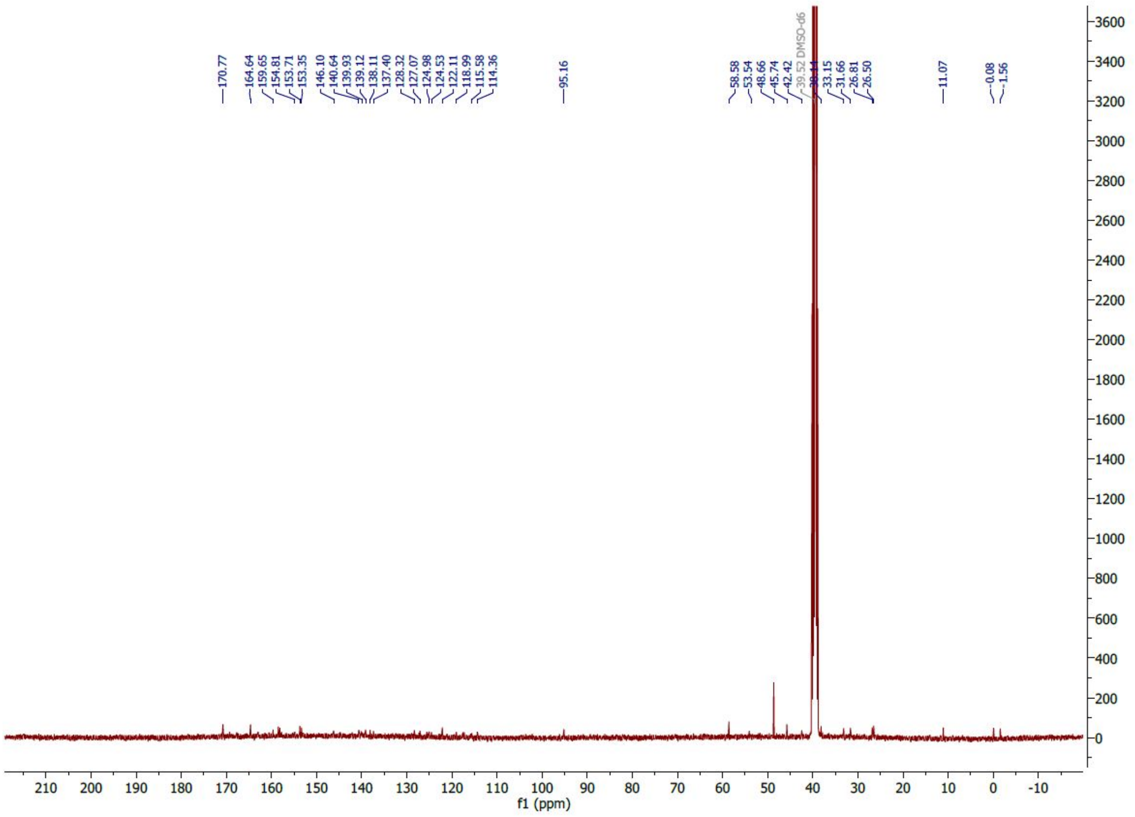
170.77
164.64
159.65
154.81
153.71
153.35
146.10
140.64
139.93
139.12
138.11
137.40
128.32
127.07
124.98
124.53
122.11
118.99
115.58
114.36
95.16
58.58
53.54
48.66
45.74
42.42
39.52 DMSO-d6
33.15
31.66
26.81
26.50
11.07
0.08
-1.56
f1 (ppm)

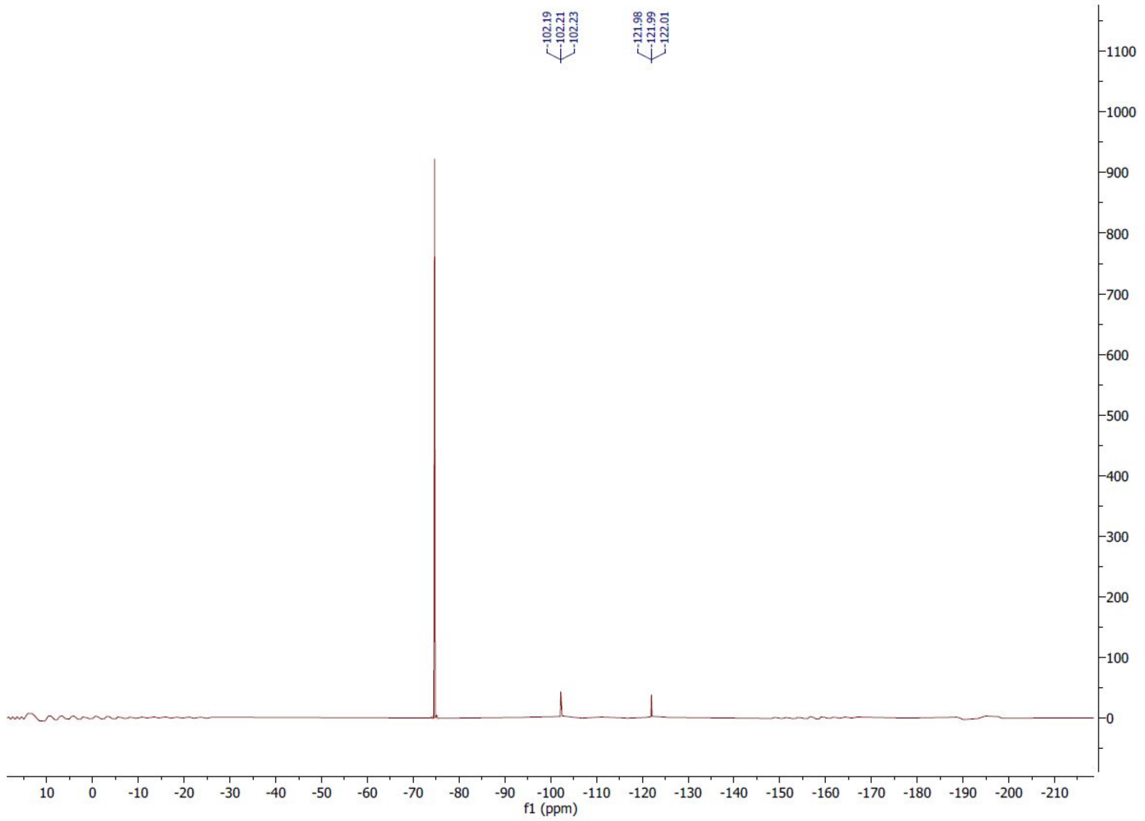
-102.19
-102.21
-102.23
-121.98
-121.99
-122.01
f1 (ppm)

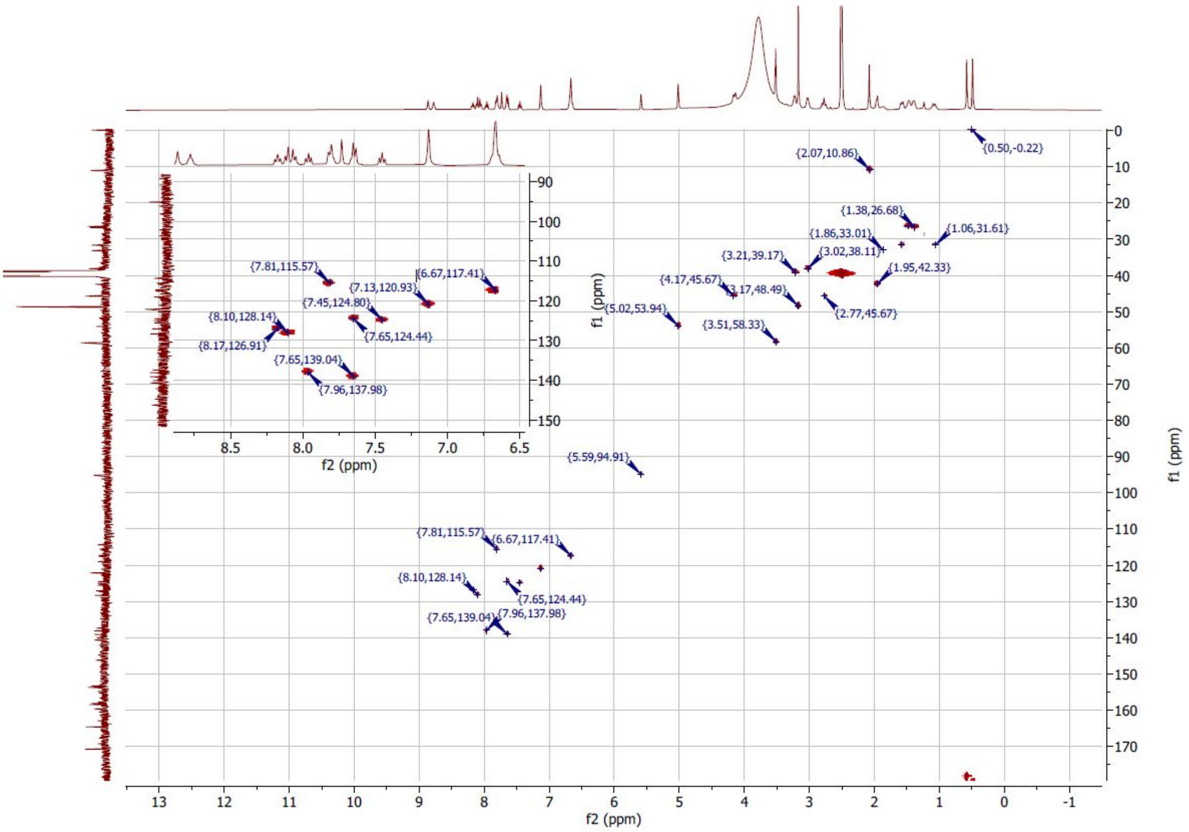
{0.50,-0.22}
{2.07,10.86}
{1.38,26.68}
{1.86,33.01}
{1.06,31.61}
{3.21,39.17}
{3.02,38.11}
{1.95,42.33}
{4.17,45.67}
{3.17,48.49}
{2.77,45.67}
{5.02,53.94}
{3.51,58.33}
{5.59,94.91}
{7.81,115.57}
{6.67,117.41}
{8.10,128.14}
{7.65,124.44}
{7.65,139.04}
{7.96,137.98}
{7.13,120.93}
{7.45,124.80}
{8.17,126.91}
f1 (ppm)
f2 (ppm)

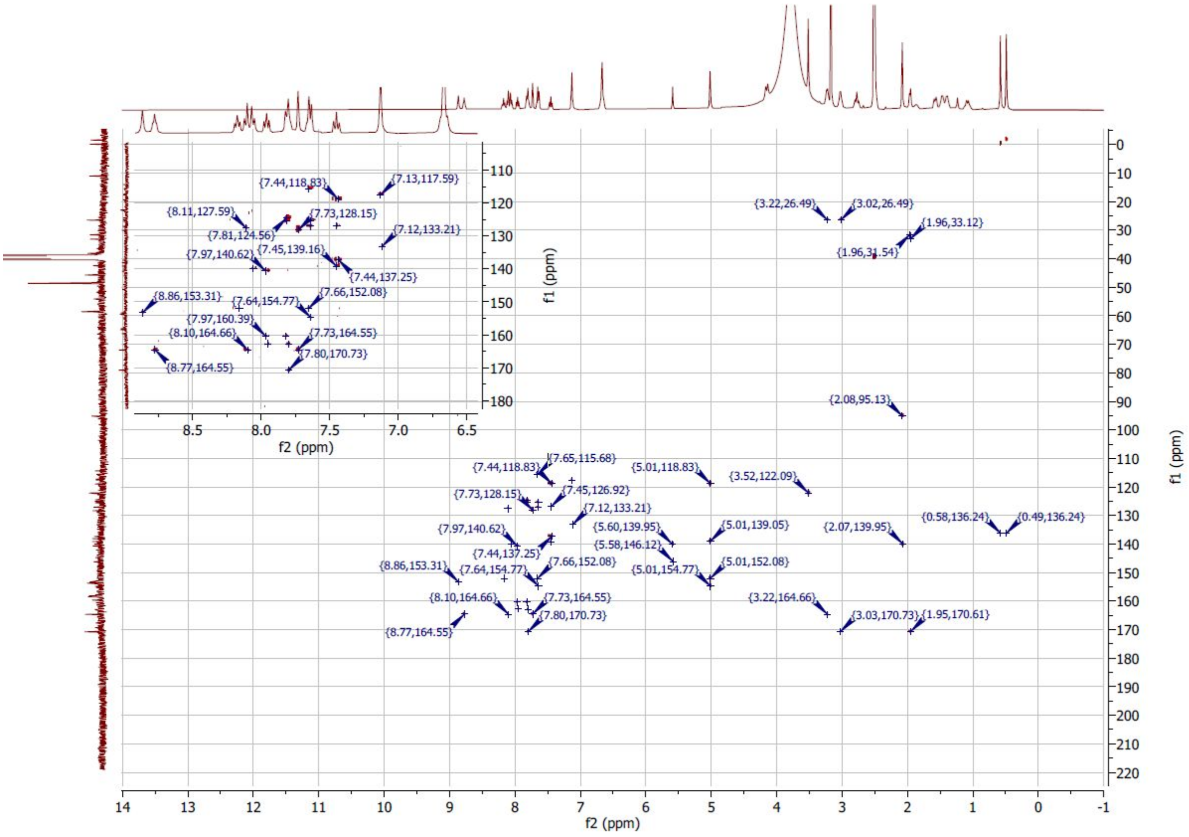
{7.44,118.83}
{7.13,117.59}
{8.11,127.59}
{7.73,128.15}
{7.81,124.56}
{7.12,133.21}
{7.97,140.62}
{7.45,139.16}
{7.44,137.25}
{8.86,153.31}
{7.64,154.77}
{7.66,152.08}
{7.97,160.39}
{8.10,164.66}
{7.73,164.55}
{7.80,170.73}
{8.77,164.55}
{3.22,26.49}
{3.02,26.49}
{1.96,33.12}
{1.96,31.54}
{2.08,95.13}
{7.65,115.68}
{5.01,118.83}
{3.52,122.09}
{7.45,126.92}
{0.58,136.24}
{0.49,136.24}
{5.60,139.95}
{5.01,139.05}
{2.07,139.95}
{5.58,146.12}
{5.01,152.08}
{5.01,154.77}
{3.22,164.66}
{3.03,170.73}
{1.95,170.61}
f1 (ppm)
f2 (ppm)

2d – SiR595-C2-C-Cen

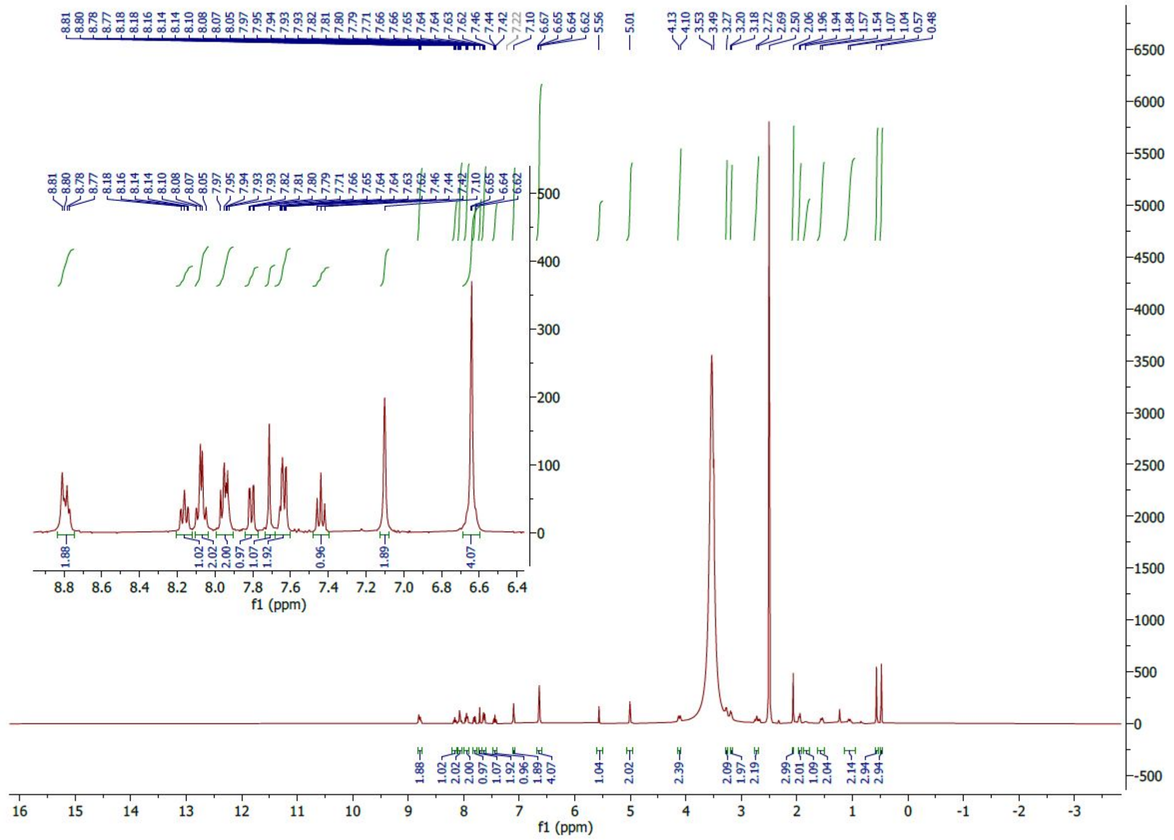

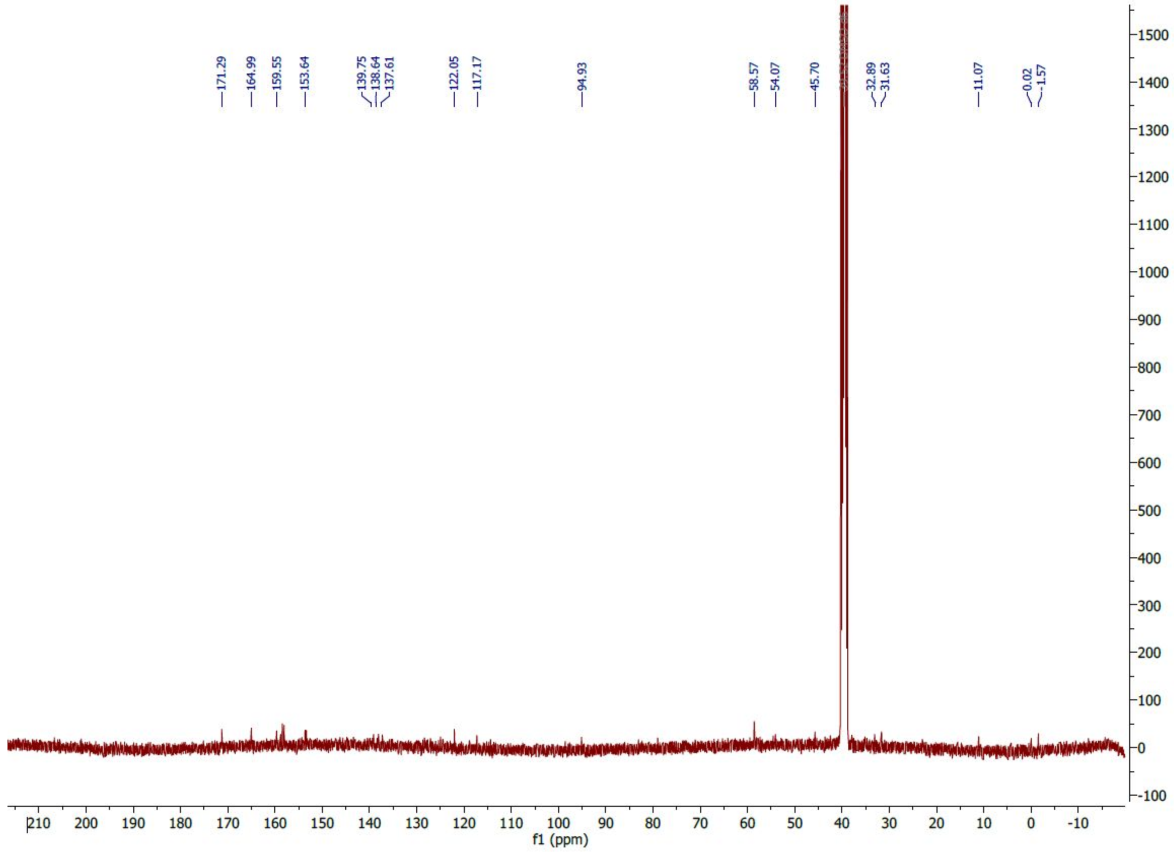

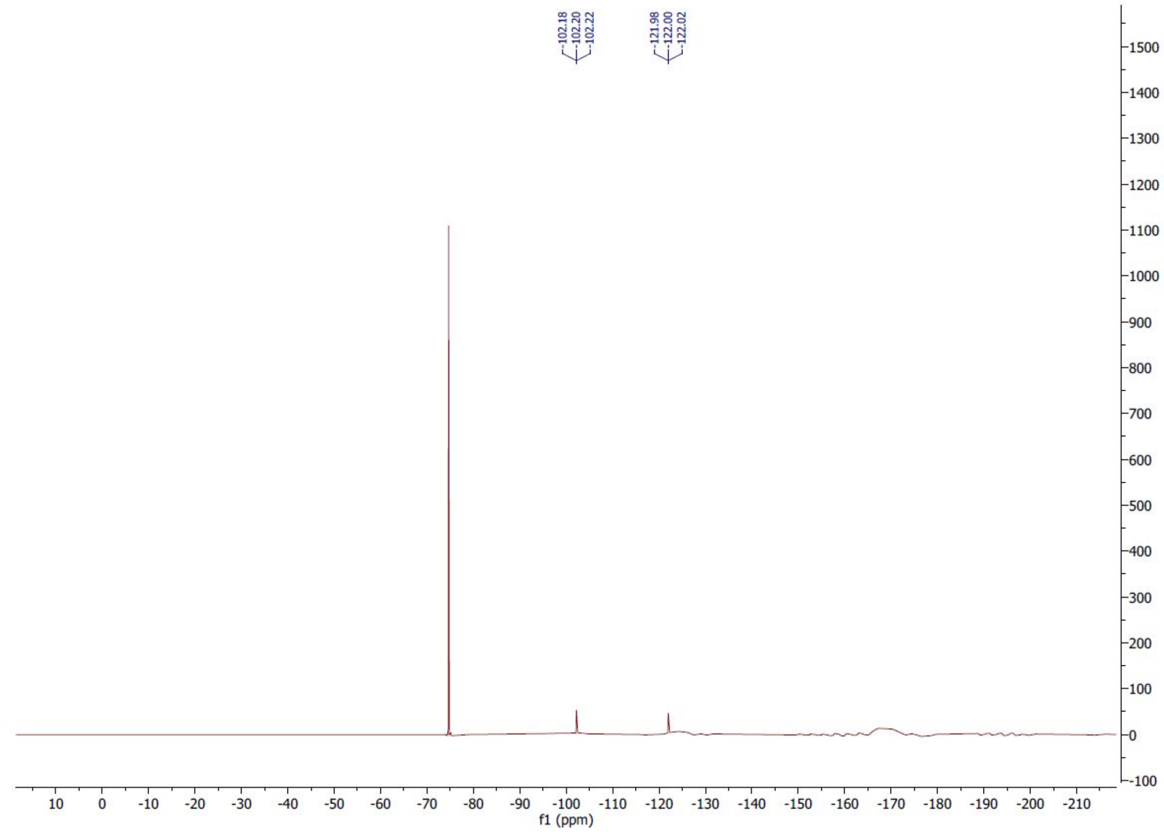
-102.18
-102.20
-102.22
-121.98
-122.00
-122.02
f1 (ppm)

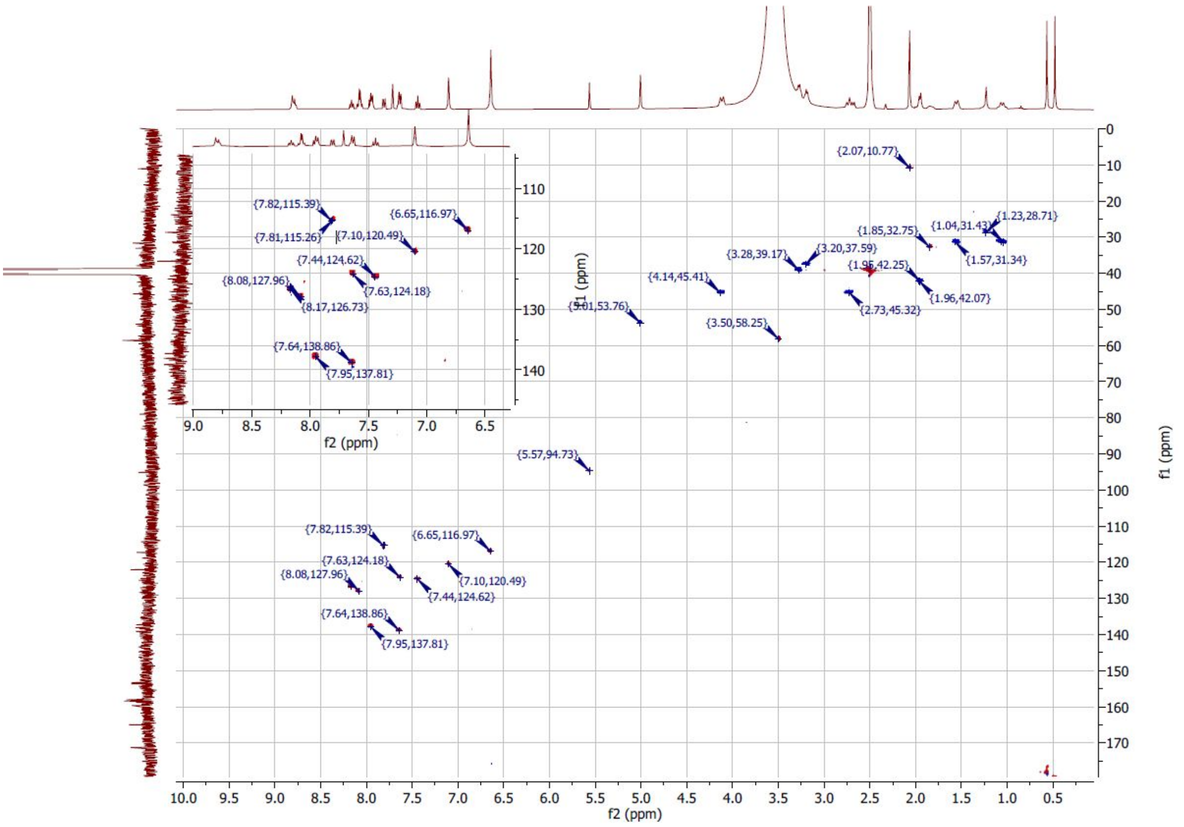
f2 (ppm)
f1 (ppm)

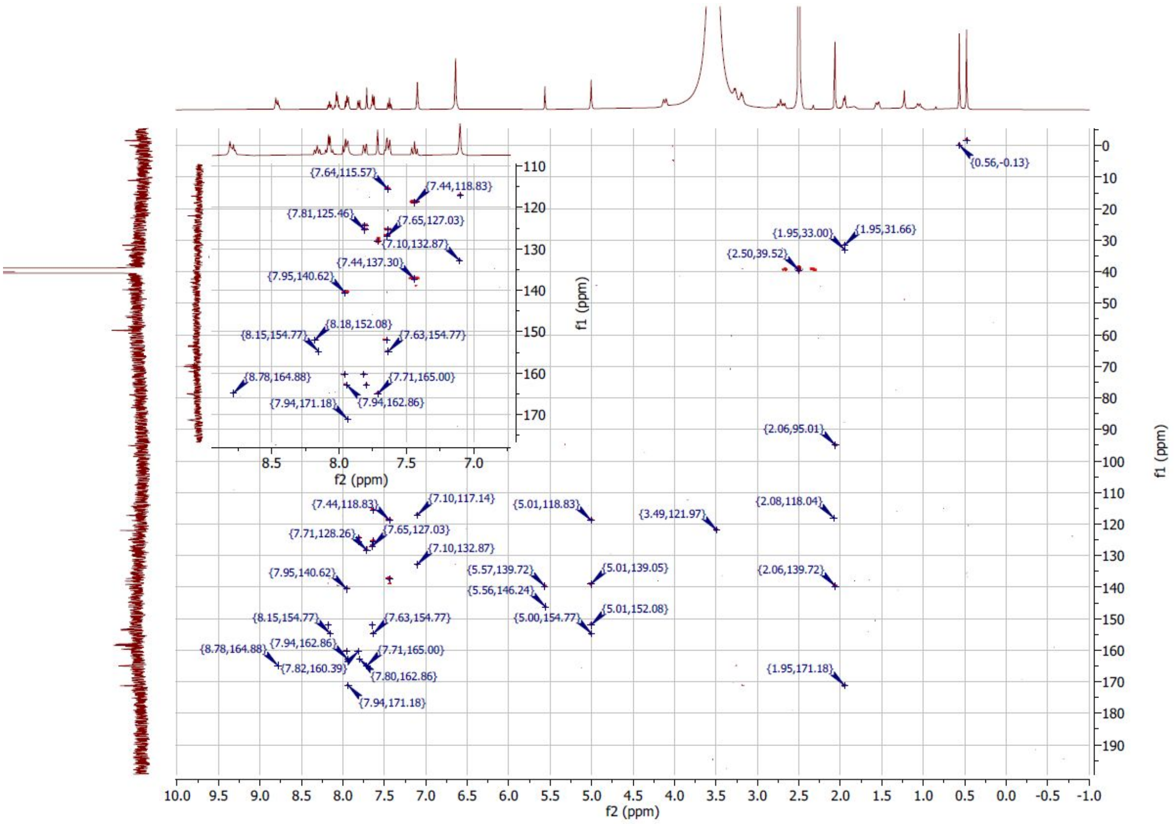

1.6.14 *tert*-butyl (2-(2-(1-(2-((2-fluoro-4-((2-fluoro-3-nitrobenzyl) sulfonyl) phenyl) thio) - 5-methoxy-6-((5-methyl-1H-pyrazol-3-yl) amino) pyrimidin-4-yl) piperidin- 4-yl) acetamido) ethyl) carbamate **27**

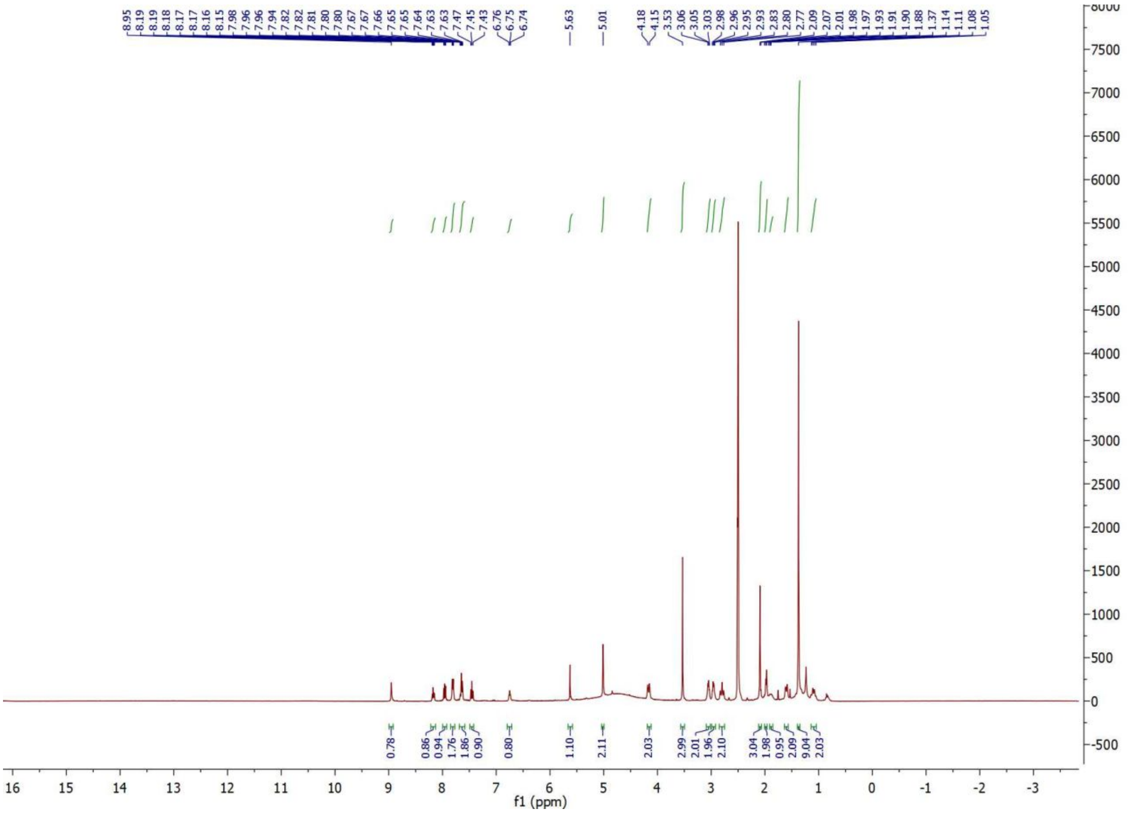

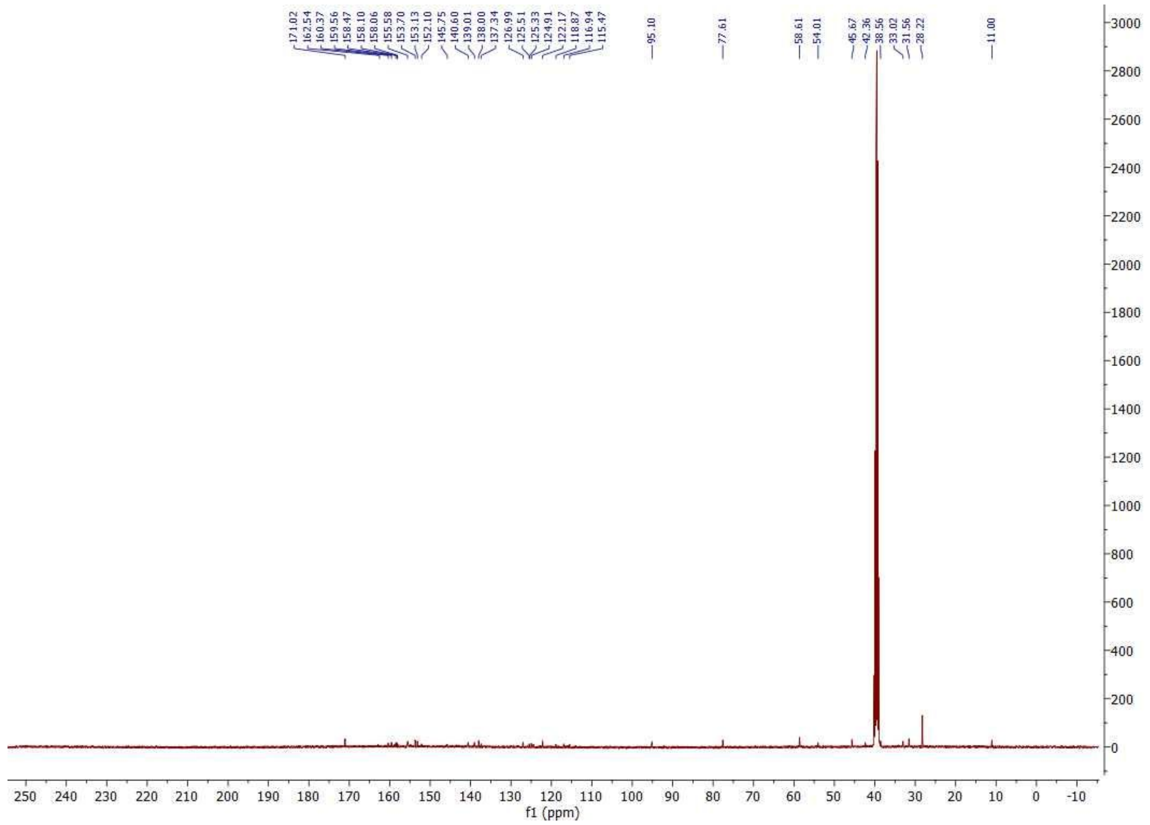
171.02
162.54
160.37
159.56
158.47
158.10
158.06
155.58
153.70
153.13
152.10
145.75
140.60
139.01
138.00
137.34
126.99
125.51
125.33
124.91
122.17
118.87
116.94
115.47
95.10
77.61
58.61
54.01
45.67
42.36
38.56
33.02
31.56
28.22
11.00
f1 (ppm)

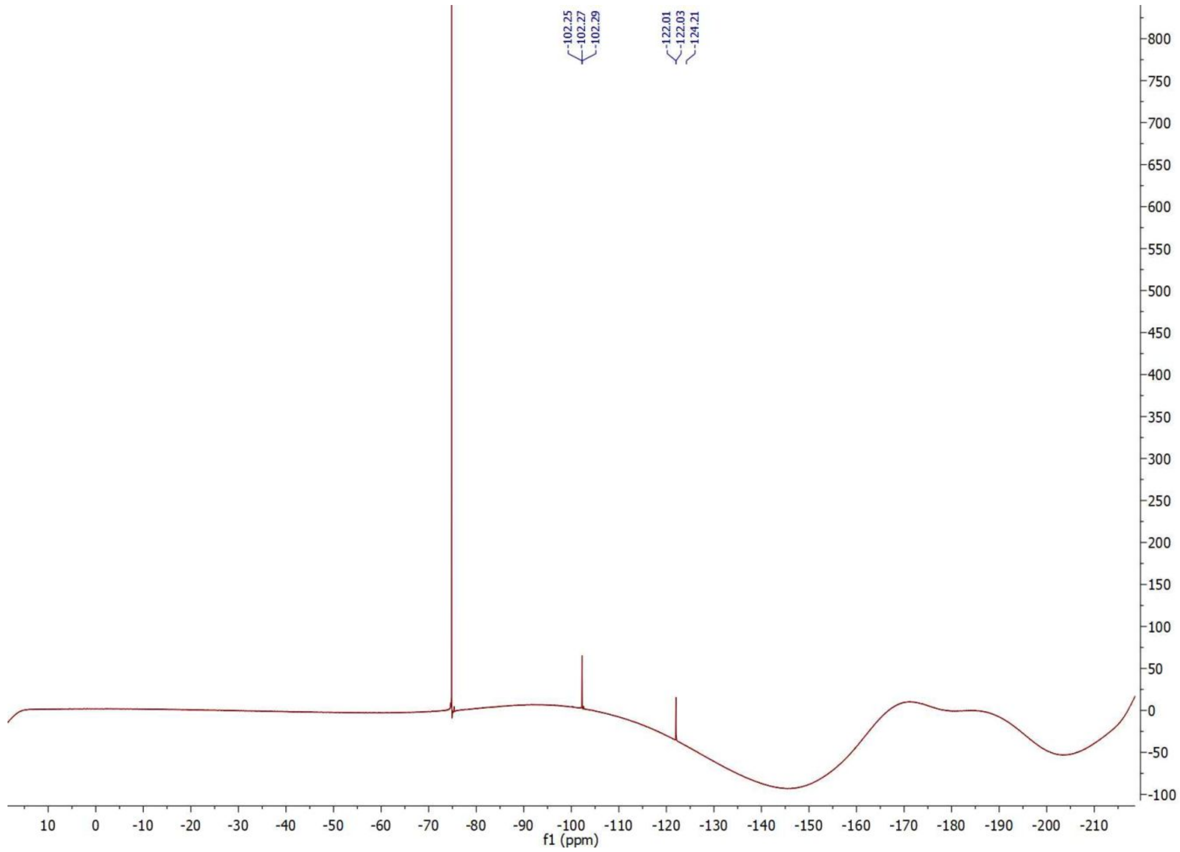
-102.25
-102.27
-102.29
-122.01
-122.03
-124.21
f1 (ppm)

1.6.15 (E)-cyclooct-4-en-1-yl (2-(2-(1-(2-((2-fluoro-4-((2-fluoro-3-nitrobenzyl) sulfonyl) phenyl) thio) -5-methoxy-6-((5-methyl-1H-pyrazol-3-yl)amino) pyrimidin-4-yl)piperidin-4-yl) acetamido)ethyl)carbamate **3**

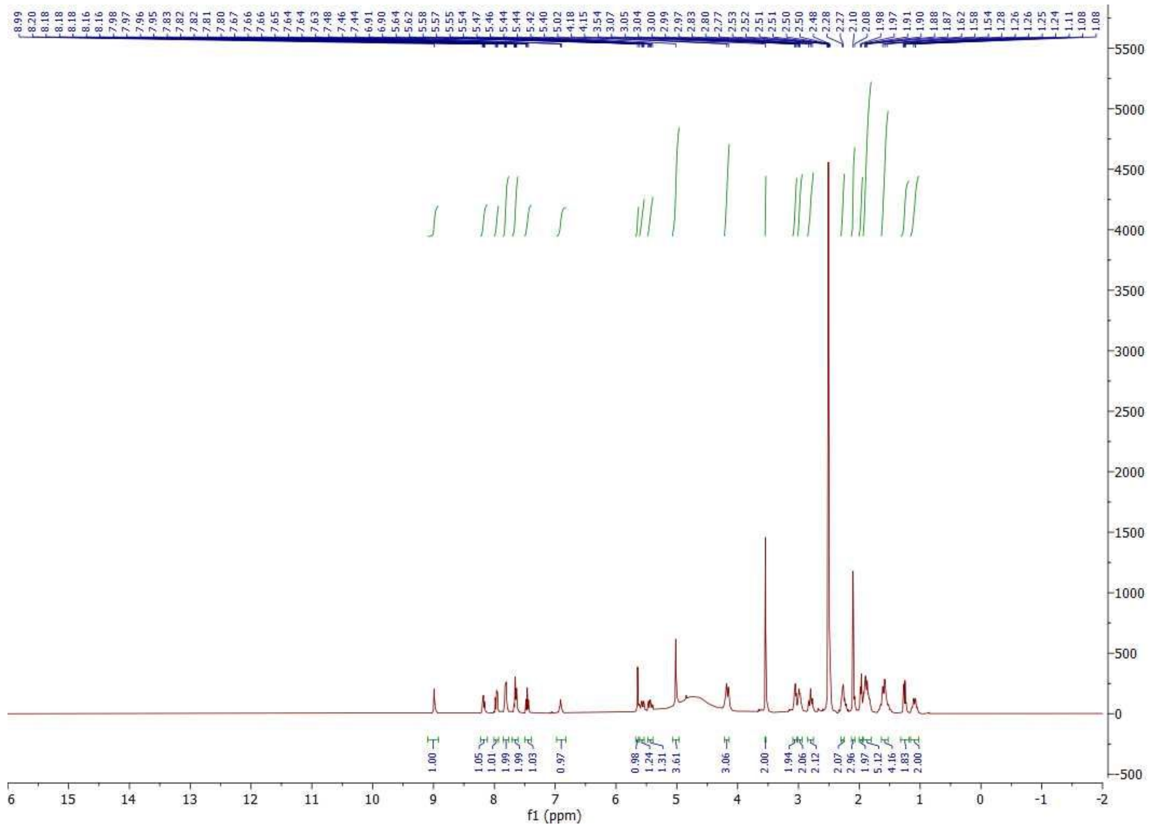

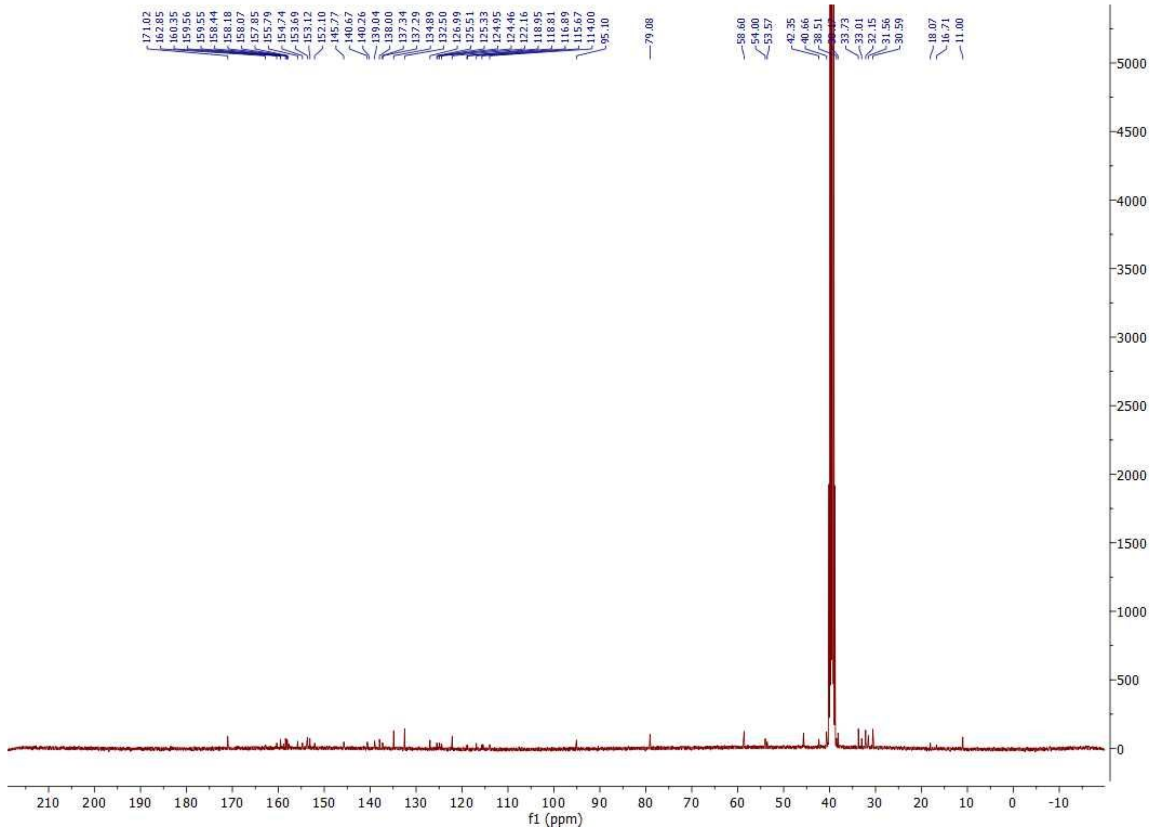

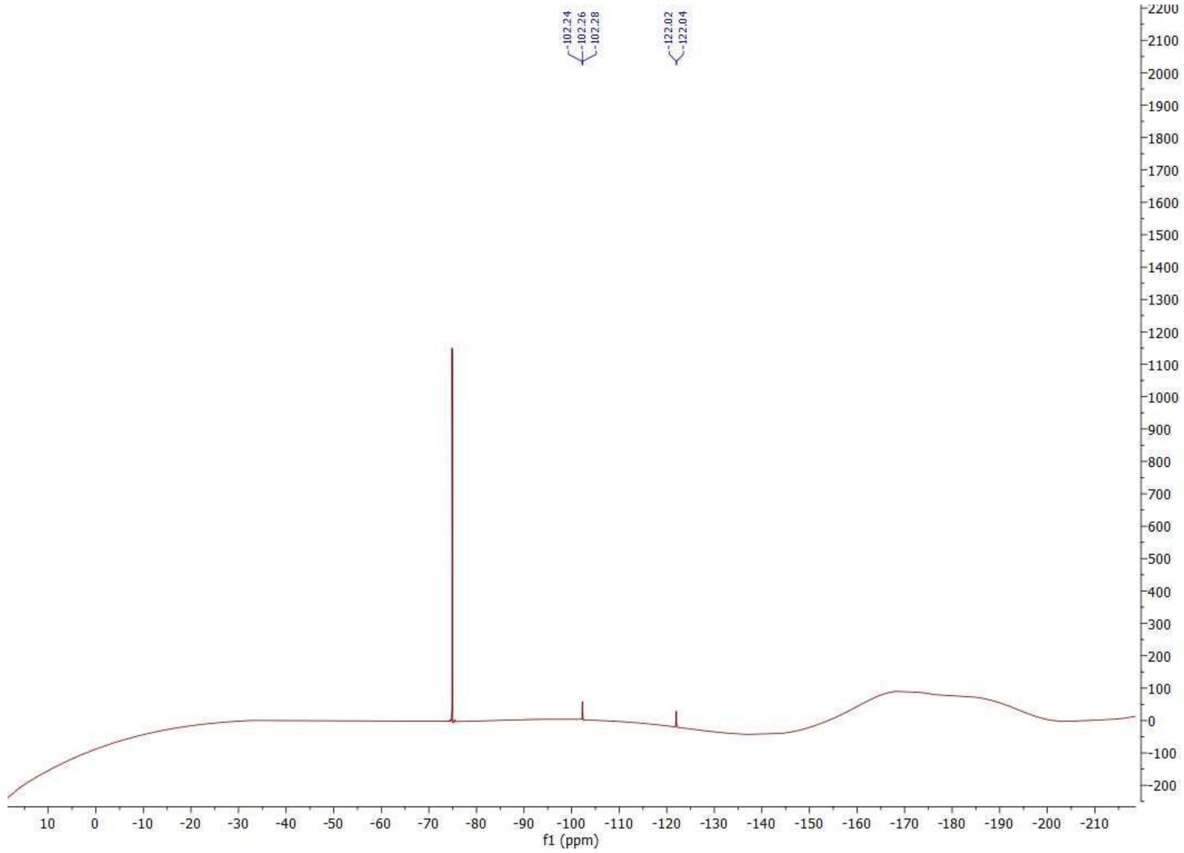

102.24
102.26
102.28
122.02
122.04
f1 (ppm)

1.6.16 SiR595-TetH **30b**

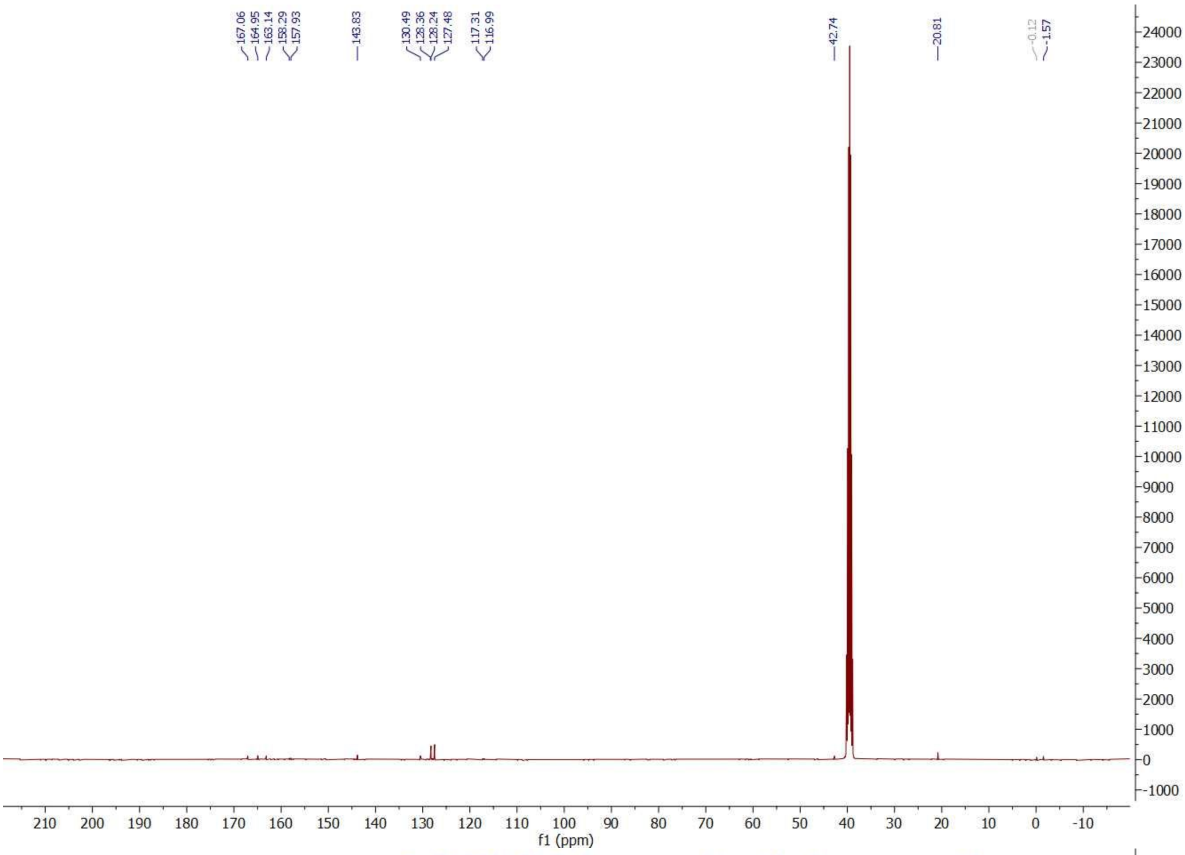

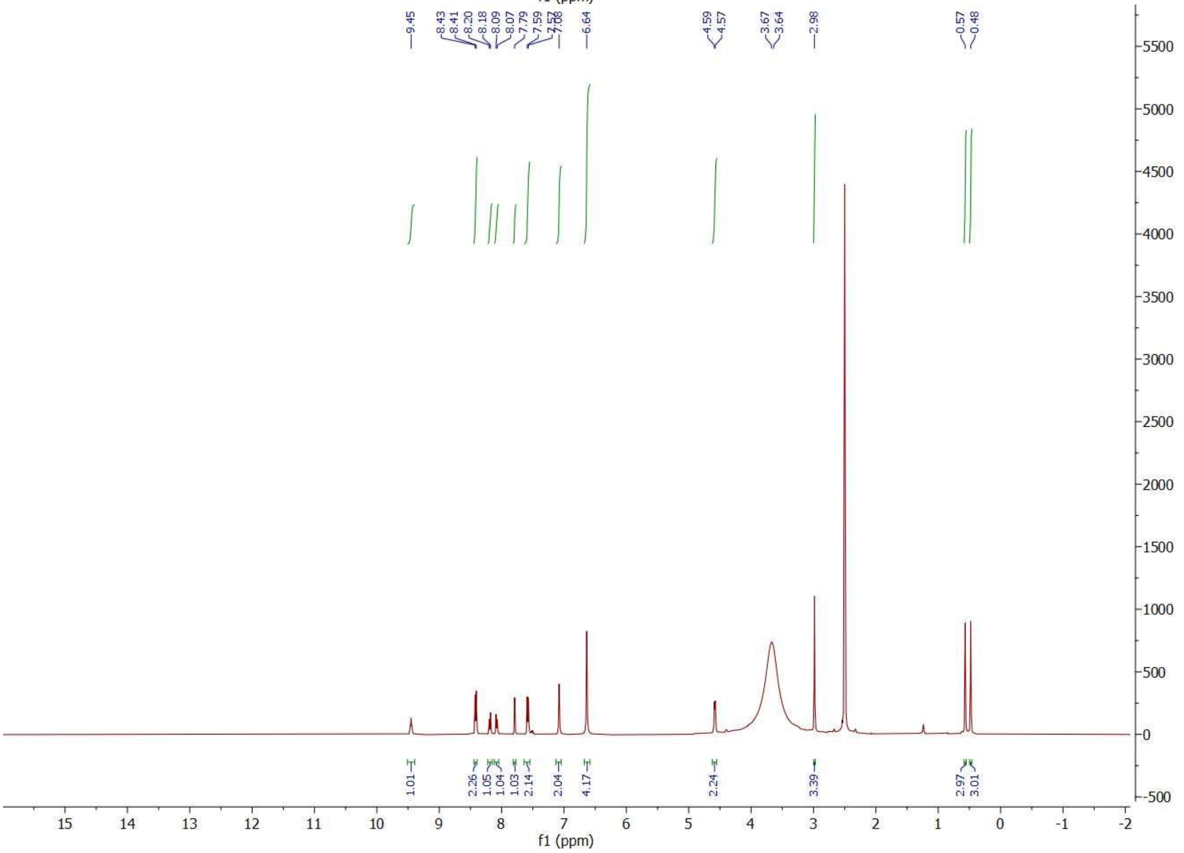

1.7 References

1. Lukinavicius, G.; Reymond, L.; D'Este, E.; Masharina, A.; Gottfert, F.; Ta, H.; Guther, A.; Fournier, M.; Rizzo, S.; Waldmann, H.; Blaukopf, C.; Sommer, C.; Gerlich, D. W.; Arndt, H. D.; Hell, S. W.; Johnsson, K., Fluorogenic probes for live-cell imaging of the cytoskeleton. *Nat Methods* **2014,** *11* (7), 731-3.

2. Rossi, A. M.; Taylor, C. W., Analysis of protein-ligand interactions by fluorescence polarization. *Nature Protocols* **2011,** *6* (3), 365-387.

3. Farrants, H.; Hiblot, J.; Griss, R.; Johnsson, K., Rational Design and Applications of Semisynthetic Modular Biosensors: SNIFITs and LUCIDs. In *Synthetic Protein Switches: Methods and Protocols*, Stein, V., Ed. Springer New York: New York, NY, 2017; pp 101-117.

4. Lukinavicius, G.; Umezawa, K.; Olivier, N.; Honigmann, A.; Yang, G.; Plass, T.; Mueller, V.; Reymond, L.; Correa, I. R., Jr.; Luo, Z. G.; Schultz, C.; Lemke, E. A.; Heppenstall, P.; Eggeling, C.; Manley, S.; Johnsson, K., A near-infrared fluorophore for live-cell super-resolution microscopy of cellular proteins. *Nat Chem* **2013,** *5* (2), 132-9.

5. Wang, L.; Tran, M.; D'Este, E.; Roberti, J.; Koch, B.; Xue, L.; Johnsson, K., A general strategy to develop cell permeable and fluorogenic probes for multicolour nanoscopy. *Nat Chem* **2020,** *12* (2), 165-172.

6. Karch, S.; Broichhagen, J.; Schneider, J.; Böning, D.; Hartmann, S.; Schmid, B.; Tripal, P.; Palmisano, R.; Alzheimer, C.; Johnsson, K.; Huth, T., A New Fluorogenic Small-Molecule Labeling Tool for Surface Diffusion Analysis and Advanced Fluorescence Imaging of β-Site Amyloid Precursor Protein-Cleaving Enzyme 1 Based on Silicone Rhodamine: SiR-BACE1. *Journal of Medicinal Chemistry* **2018,** *61* (14), 6121-6139.

7. Werther, P.; Yserentant, K.; Braun, F.; Grußmayer, K.; Navikas, V.; Yu, M.; Zhang, Z.; Ziegler, M. J.; Mayer, C.; Gralak, A. J.; Busch, M.; Chi, W.; Rominger, F.; Radenovic, A.; Liu, X.; Lemke, E. A.; Buckup, T.; Herten, D.-P.; Wombacher, R., Bio-orthogonal Red and Far-Red Fluorogenic Probes for Wash-Free Live-Cell and Super-resolution Microscopy. *ACS Central Science* **2021**.
